# Integrating Genetic and Genomic Analyses of Combined Health Data Across Ecotypes to Improve Disease Resistance in Indigenous African Chickens

**DOI:** 10.3389/fgene.2020.543890

**Published:** 2020-10-09

**Authors:** Georgios Banos, Victoria Lindsay, Takele T. Desta, Judy Bettridge, Enrique Sanchez-Molano, Adriana Vallejo-Trujillo, Oswald Matika, Tadelle Dessie, Paul Wigley, Robert M. Christley, Peter Kaiser, Olivier Hanotte, Androniki Psifidi

**Affiliations:** ^1^The Roslin Institute, The University of Edinburgh, Edinburgh, United Kingdom; ^2^Scotland’s Rural College, Edinburgh, United Kingdom; ^3^Centre for Tropical Livestock Genetics and Health, Edinburgh, United Kingdom; ^4^Royal Veterinary College, University of London, London, United Kingdom; ^5^School of Life Sciences, University of Nottingham, Nottingham, United Kingdom; ^6^Institute of Infection and Global Health, University of Liverpool, Liverpool, United Kingdom; ^7^LiveGene – Centre for Tropical Livestock Genetics and Health, International Livestock Research Institute, Addis Ababa, Ethiopia; ^8^Natural Resources Institute, University of Greenwich, London, United Kingdom

**Keywords:** GWAS, GEBV, WGS, indigenous chickens, body weight, infectious diseases, antibody responses, Ethiopia

## Abstract

Poultry play an important role in the agriculture of many African countries. The majority of chickens in sub-Saharan Africa are indigenous, raised in villages under semi-scavenging conditions. Vaccinations and biosecurity measures rarely apply, and infectious diseases remain a major cause of mortality and reduced productivity. Genomic selection for disease resistance offers a potentially sustainable solution but this requires sufficient numbers of individual birds with genomic and phenotypic data, which is often a challenge to collect in the small populations of indigenous chicken ecotypes. The use of information across-ecotypes presents an attractive possibility to increase the relevant numbers and the accuracy of genomic selection. In this study, we performed a joint analysis of two distinct Ethiopian indigenous chicken ecotypes to investigate the genomic architecture of important health and productivity traits and explore the feasibility of conducting genomic selection across-ecotype. Phenotypic traits considered were antibody response to Infectious Bursal Disease (IBDV), Marek’s Disease (MDV), Fowl Cholera (PM) and Fowl Typhoid (SG), resistance to *Eimeria* and cestode parasitism, and productivity [body weight and body condition score (BCS)]. Combined data from the two chicken ecotypes, Horro (*n* = 384) and Jarso (*n* = 376), were jointly analyzed for genetic parameter estimation, genome-wide association studies (GWAS), genomic breeding value (GEBVs) calculation, genomic predictions, whole-genome sequencing (WGS), and pathways analyses. Estimates of across-ecotype heritability were significant and moderate in magnitude (0.22–0.47) for all traits except for SG and BCS. GWAS identified several significant genomic associations with health and productivity traits. The WGS analysis revealed putative candidate genes and mutations for IBDV (*TOLLIP, ANGPTL5, BCL9, THEMIS2*), MDV (*GRM7*), SG (*MAP3K21*), *Eimeria* (*TOM1L1*) and cestodes (*TNFAIP1, ATG9A, NOS2*) parasitism, which warrant further investigation. Reliability of GEBVs increased compared to within-ecotype calculations but accuracy of genomic prediction did not, probably because the genetic distance between the two ecotypes offset the benefit from increased sample size. However, for some traits genomic prediction was only feasible in across-ecotype analysis. Our results generally underpin the potential of genomic selection to enhance health and productivity across-ecotypes. Future studies should establish the required minimum sample size and genetic similarity between ecotypes to ensure accurate joint genomic selection.

## Introduction

Village poultry are important in low- and middle-income countries around the world (Hassan et al., 2004; Khobondo et al., 2015) and, therefore, are the focus of many development programs (Dana et al., 2010, 2011; Dinka et al., 2010; Ngeno, 2011; Magothe et al., 2012; Khobondo et al., 2015). However, such programs have often been unsustainable (Dinka et al., 2010; Dana et al., 2011). For example, in Ethiopia, previous poultry development programs concentrated on ways to increase the productivity of chickens by introducing commercial (exotic) breeds that perform highly under (semi) intensive management conditions. Whilst this has been a relatively successful approach in peri-urban areas (FAO, 2008), it has not translated well into rural areas and smallholder farmers. In the latter, system productivity is usually low and constrained by disease, predation and scarcity of feed (Pica-Ciamarra and Dhawan, 2009), among other factors. However, village chickens in these settings possess the advantages of being widely accessible and well-adapted to the local environmental conditions, while requiring lower inputs compared to commercial chickens (Psifidi et al., 2016; Bettridge et al., 2018). Moreover, consumers exhibit a strong preference for indigenous chicken meat and eggs compared to commercial types (Dana et al., 2010), which suggests that there is a viable market to be supplied. Besides the often unsustainable attempts to introduce exotic commercial birds (Dinka et al., 2010; Dana et al., 2011) and/or implement cross-breeding programs (Magothe et al., 2012; Khobondo et al., 2015), genetic improvement of the indigenous African village chickens presents a promising avenue. In fact, a breeding program is already in place aiming to improve both bird growth and egg production of local Ethiopian chickens (Wondmeneh et al., 2014). Moreover, in addition to focusing on increasing productivity, a focus toward raising the efficiency of the production system has also been considered beneficial (Bettridge et al., 2018). This is especially true for rural smallholders who may be able to make significant gains in income and nutrition, but of necessity can afford little in the way of economic or opportunity costs that some of the previous chicken development programs in Ethiopia have required.

We previously used a high-density genome-wide array to genotype two distinct unselected Ethiopian indigenous chicken ecotypes (village chickens from the Jarso and Horro geographic regions) and performed genomic studies on each ecotype separately to investigate the genetic architecture of six major infectious diseases (Marek’s Disease, Infectious Bursal Disease, Fowl Cholera, Fowl Typhoid, and *Eimeria* and cestode parasitism) and two production traits (live body weight and body condition score) (Psifidi et al., 2016). The outcomes of that study suggested that concomitant genetic improvement for enhanced disease resistance and productivity in each indigenous chicken ecotype is feasible, although small population size would challenge the accuracy of selection. Indeed, successful genomic selection programs require sufficient numbers of individual birds with genotypic and phenotypic data, which may be a challenge within most indigenous chicken ecotypes. Therefore, the use of information across multiple ecotype populations presents, in principle, an attractive alternative to increase the relevant numbers and the accuracy of genomic selection. This has not been investigated in chickens before although there is evidence of benefit in across-breed genomic selection in other species such as cattle (Hozé et al., 2014; Iheshiulor et al., 2016).

In the present study, we extended the previous work of Psifidi et al. (2016) described above and jointly analyzed the same individual bird data from the Horro and Jarso indigenous ecotypes in order to increase the sample size and identify common genomic regions controlling the traits of interest. Moreover, we generated and analyzed whole-genome sequencing data of the two ecotypes to identify candidate genes and mutations within the relevant genomic regions, and performed pathway and network analysis in order to increase our understanding of the genomic architecture of the traits under investigation. We also examined the feasibility of joint across-ecotype genomic selection aiming to enhance antibody response, resistance to parasitic infectious diseases and productivity.

## Materials and Methods

### Ethics Statement

All work was conducted with the approval of the University of Liverpool Research Ethics Committee (reference RETH000410).

### Animals, Sampling, and Phenotyping

Details of the bird populations used in the present study, sampling strategy implemented and phenotyping of individual birds have been described in detail in Psifidi et al. (2016). Briefly, the two indigenous ecotypes were located in the Jarso geographic region in arid eastern Ethiopia and in the Horro region in sub-humid western Ethiopia (Desta et al., 2013). These two regions are about 900 km far away from each other. Multistage cross-sectional sampling was performed considering two market sheds per geographic region, each represented by two villages; within each village, two chickens, over 6 months of age, were randomly sampled from each of 25 households. A total of 760 individual bird samples, 376 from Jarso and 384 from Horro, were collected in four rounds over 2 years at 6 month intervals, covering the pre- and post-rainy seasons of data collection (Desta et al., 2013; Bettridge et al., 2014).

Fresh feces were collected for parasite egg measurements, and brachial blood collected into tubes with sodium citrate for serology and on FTA cards for DNA extraction from each of the birds. *Eimeria* spp. oocysts and cestode spp. eggs were counted with a modified version of the concentration McMaster technique as described in Permin and Hansen (1998). Antibody titers for Infectious Bursal Disease (IBDV) were measured using a Flockscreen antibody ELISA kit (x-OvO, Dunfermline, United Kingdom). Antibody titers for Marek’s Disease (MDV), Fowl Cholera (*Pasteurella multocida*, henceforth PM) and Fowl Typhoid (*Salmonella enterica serovar Gallinarum*, henceforth SG) were measured using in-house developed ELISAs, as described in Bettridge et al. (2014). Live bodyweight (BW, g) and body condition score (BCS) on a 0–3 scale (Gregory and Robins, 1998) were also recorded on each bird. All data except for BCS were log_10_-transformed in order to normalize the respective distributions.

All birds were genotyped using the high density single nucleotide polymorphism (SNP) genome-wide DNA array (Affymetrix^®^ Axiom <^®^ HD) consisting of 580,954 SNPs uniformly distributed across the genome (Kranis et al., 2013). These data were subjected to the following quality control thresholds using PLINK v1.09 (Purcell et al., 2007): minor allele frequency < 0.05, call rate < 95%, and Hardy-Weinberg equilibrium (*P* < 10^–6^). After quality control, 359,470 SNP markers were kept for further analyses, distributed across all chromosomes.

### Genetic Parameter Estimation

Variance components and genetic parameters were estimated for the combined population of the two ecotypes as described previously in detail in Psifidi et al. (2016) for all traits, using a mixed linear univariate model that included the fixed effects of geographic region, village, calendar season, sex and age of bird, and ELISA plate (for antibody titers only), and the random additive genetic effect of the individual bird. Genomic relationships among birds were calculated using a combined kinship matrix obtained with the Genome-wide Efficient Mixed Model Association (GEMMA) algorithm (Zhou and Stephens, 2014) and were included in the analyses. Estimates of variance components were obtained with the method of Restricted Maximum Likelihood and used to calculate the heritability of each trait as the ratio of the additive genetic to the total phenotypic variance. Bivariate analyses were subsequently conducted with the same model to estimate phenotypic and genetic correlations among the studied traits. All analyses were performed using the ASReml 3.0 software (Gilmour et al., 2009).

### Genome-Wide Association Studies

For each studied trait, a genome-wide association analysis (GWAS) was performed using combined data across the two ecotypes. The GEMMA algorithm (Zhou and Stephens, 2014) and the same linear mixed model as in the genetic parameter estimation step described above were used. After Bonferroni correction for multiple testing, significance thresholds were *P* ≤ 1.39E-07 and 2.78E-06 for genome-wide significant (*P* ≤ 0.05) and suggestive (one false positive per genome scan) levels, respectively, corresponding to −log_10_(P) of 6.85 and 5.55.

All significant and suggestive significant SNP markers identified in the GWAS were annotated using the galGal4 assembly according to the Affymetrix map file and their locations were then re-mapped from galGal4 to galGal6 using UCSC liftOver tool (Hinrichs et al., 2006). In addition, the genes located within 100 kb upstream and 100 kb downstream of these markers were also annotated using the BioMart (Smedley et al., 2015) data mining tool^1^ within the Ensembl database and the galGal6 assembly. We chose these 200-kb windows based on the average Linkage Disequilibrium (LD) calculated previously for the Horro and Jarso chicken populations (Desta, 2015); mild LD (*r*^2^ ∼ 0.2) rarely exceeds 100 kb. This allowed us to catalog and create lists of all genes located around the identified significant markers that are potentially associated with antibody response, resistance to parasitic infection and production traits.

### Whole Genome Sequencing Analysis

Six Horro and 14 Jarso birds were whole-genome sequenced (WGS; paired-end with a read length of 150 bp and average coverage of 40 X) on an Illumina HiSeqX platform. The sequencing reads were mapped to galGal6 using BWA-MEM (Li, 2013) and variant calling was performed using GATK tools according to Best Practices (DePristo et al., 2011; Poplin et al., 2018). Variant filtration was performed using GATK VQSR with 1 million validated SNPs and more than 20 million known chicken SNPs (from dbSNP).

Based on the GWAS results, the candidate regions (200 kb windows around each of the significant SNP) for each of the studied traits were extracted from the WGS data for both the Horro and Jarso chickens. The identified variants in these regions were annotated and their predicted effects on the encoding protein were assessed using the Ensembl Variant Effect Predictor tool (McLaren et al., 2016). The variants with a predicted high (stop gained/lost, start lost, frameshift variant, or splice acceptor/donor variants) and moderate (in-frame insertion/deletion, missense, or protein altering variants) impact were filtered out and further analyzed to highlight putative candidate genes and variants.

### Pathways and Network Analysis

Pathways and network analyses were performed in the combined population based on the GWAS results. We reasoned that the corresponding quantitative trait loci (QTL) regions would contain genes contributing to common pathways associated with each of the studied traits. Therefore, initially the lists of annotated genes located within the QTL regions for each bird phenotype were analyzed using the Ingenuity Pathway Analysis (IPA) program (Krämer et al., 2014). We sought evidence of gene set enrichment in order to identify potential underlying canonical pathways and networks associated with the studied traits. IPA constructs multiple possible upstream regulators, pathways and networks serving as hypotheses for the biological mechanism underlying the data based on a large-scale causal network derived from the Ingenuity Knowledge Base. Subsequently, IPA infers the most suitable pathways and networks based on their statistical significance (*P*-values obtained using Fisher’s exact test), after correcting for a baseline threshold (Krämer et al., 2014).

### Estimation of Genomic Breeding Values and Genomic Predictions

Genomic breeding values (GEBVs) were simultaneously estimated for all birds of the two ecotypes using a genomic best linear unbiased prediction model including the same effects as the genetic parameter estimation model [fixed effects of geographic region, village, calendar season, sex and age of bird, and ELISA plate (for antibody titers only], and the random additive genetic effect of the individual bird). Reliability of the GEBV of each bird and trait was calculated as:

$$R\,e\,l=1-\frac{P\,E\,V}{\sigma_{A}^{2}}$$

where PEV is the prediction error variance of the GEBV and σ^2^_*A*_ is the additive genetic variance of the trait.

A threefold cross-validation was then performed to assess the accuracy of genomic predictions for the combined population of Jarso and Horro ecotypes. Briefly, we divided the dataset into three subsets, each consisting of approximately equal proportions of birds from each ecotype, and predicted the GEBVs in each subset (validation subset) based on the analysis of genotypic and phenotypic data from the other two (reference subsets). This was repeated three times for each studied trait, with different random sample division in each repeat to reduce the effect produced by specific random combinations of animals within subsets on prediction. Accuracy of genomic prediction was defined as the Pearson correlation coefficient between the GEBVs in the validation subset and the corresponding adjusted phenotypic values. The latter were the residuals from a fixed-effect model analysis including all fixed effects described in the genetic parameter estimation step. A second accuracy measurement was derived by dividing the above-mentioned correlation by the square root of the trait heritability.

Derivation of GEBVs and cross-validation of genomic predictions were also performed separately within each ecotype for comparison with the across-ecotype results.

## Results

### Descriptive Statistics and Genetic Parameters of Studied Traits

Means and standard deviations of the studied traits across the two ecotypes are presented in Table 1. Heritability estimates of these traits for the combined population of the two ecotypes are summarized in Table 2. Moderately high estimates were derived for MDV and IBDV antibody responses, and for BW. A moderately low heritability was estimated for PM antibody responses, and cestodes and *Eimeria* parasitism, while a low heritability was estimated for SG antibody responses and BCS. All trait heritability estimates were significantly (*P* < 0.05) greater than zero with the exception of SG and BCS.

**TABLE 1:** Means and standard deviations (STD) of antibody responses, resistance to parasitic infections and production traits across Horro and Jarso ecotypes.

**Trait**	**Mean**	***STD***
IBDV	0.04	0.15
MDV	0.21	0.58
SG	1.31	1.40
PM	0.96	0.77
*Eimeria*	38.83	202.16
Cestode	2.91	21.57
Body weight	1.33	0.31
BCS	1.55	0.60

*IBDV log transformed antibody titers measured for Infectious Bursal Disease virus, *MDV* log-transformed antibody titers measured for Marek’s Disease virus, *SG* log-transformed antibody titers measured for *Salmonella enterica serovar Gallinarum*, *PM* log-transformed antibody titers measured for *Pasteurella multocida*; *Eimeria* resistance to *Eimeria* parasitism (log-transformed egg counts/gr of fecal), *Cestodes* resistance to cestodes parasitism (log-transformed egg counts/gr of fecal), *BW* live body weight (log-transformed g), *BCS* body condition score (scale 0 to 3).*

**TABLE 2:** Heritability estimates (h^2^) for antibody responses, resistance to parasitic infections and production traits across Horro and Jarso ecotypes.

	**IBDV**	**MDV**	**SG**	**PM**	***Eimeria***	**Cestodes**	**BW**	**BCS**
*h*^2^	**0.47**	**0.42**	0.07	**0.29**	**0.22**	**0.28**	**0.43**	0.16
SE	**0.10**	**0.11**	0.10	**0.10**	**0.10**	**0.11**	**0.09**	0.10

*Heritability estimates in bold attained statistical significance (*P* < 0.05). *IBDV* log transformed antibody titers measured for Infectious Bursal Disease virus, *MDV* log-transformed antibody titers measured for Marek’s Disease virus, *SG* log-transformed antibody titers measured for *Salmonella enterica serovar Gallinarum*, *PM* log-transformed antibody titers measured for *Pasteurella multocida*; *Eimeria* resistance to *Eimeria* parasitism (log-transformed egg counts/gr of fecal), *Cestodes* resistance to cestodes parasitism (log-transformed egg counts/gr of fecal), *BW* live body weight (log-transformed g), *BCS* body condition score (scale 0 to 3); *SE*, standard error.*

Significantly (*P* < 0.05) different from zero phenotypic and genetic correlations were identified pairwise between PM and SG antibody responses, IBDV and SG antibody responses, and BW and BCS (Table 3). Moreover, a significant negative phenotypic and genetic correlation was estimated between *Eimeria* and cestodes parasitism. There were no significant genetic correlations of either production trait with any of the antibody response or parasitic infection traits.

**TABLE 3:** Genetic (above diagonal) and phenotypic (below diagonal) correlations between traits studied across Horro and Jarso ecotypes (standard errors in parentheses).

	**IBDV**	**MDV**	**PM**	**SG**	**Cestodes**	***Eimeria***	**BW**	**BCS**
IBDV		0.03 (0.18)	0.16 (0.23)	**0.81** **(0.41)**	0.13 (0.23)	0.05 (0.25)	−0.07 (0.16)	−0.30 (0.30)
MDV	0.05 (0.04)		0.14 (0.25)	0.19 (0.45)	−0.26 (0.25)	−0.05 (0.27)	0.01 (0.18)	−0.12 (0.31)
PM	0.05 (0.04)	−0.00 (0.04)		**1.36 (0.43)**	−0.47 (0.32)	−0.55 (0.34)	0.25 (0.23)	0.23 (0.39)
SG	**0.13 (0.04)**	−0.03 (0.04)	**0.40 (0.02)**		−0.67 (0.75)	−0.06 (0.60)	0.41 (0.51)	0.47 (0.72)
Cestodes	−0.02 (0.04)	0.02 (0.04)	0.01 (0.04)	0.01 (0.04)		−**0.63 (0.27)**	0.14 (0.21)	0.32 (0.38)
*Eimeria*	−0.03 (0.04)	0.04 (0.04)	−**0.11 (0.04)**	−**0.10 (0.04)**	−**0.17 (0.04)**		−0.03 (0.24)	1.23 (0.79)
BW	−0.01 (0.04)	0.01 (0.04)	**0.11 (0.04)**	**0.09 (0.04)**	0.01 (0.04)	−0.02 (0.04)		**0.54 (0.21)**
BCS	0.06 (0.04)	0.02 (0.04)	0.04 (0.04)	−0.01 (0.04)	−0.01 (0.04)	0.05 (0.04)	**0.30 (0.04)**	

*Estimates in bold attained statistical significance (*P* < 0.05). *IBDV* antibody titers measured for Infectious Bursal Disease virus, *MDV* antibody titers measured for Marek’s Disease virus, *SG* antibody titers measured for *Salmonella enterica serovar Gallinarum*, *PM* antibody titers measured for *Pasteurella multocida*; *Eimeria* resistance to *Eimeria* parasitism, *Cestodes* resistance to cestodes parasitism, *BW* live body weight, *BCS* body condition score.*

### Genome-Wide Association Studies

**FIGURE 1:**
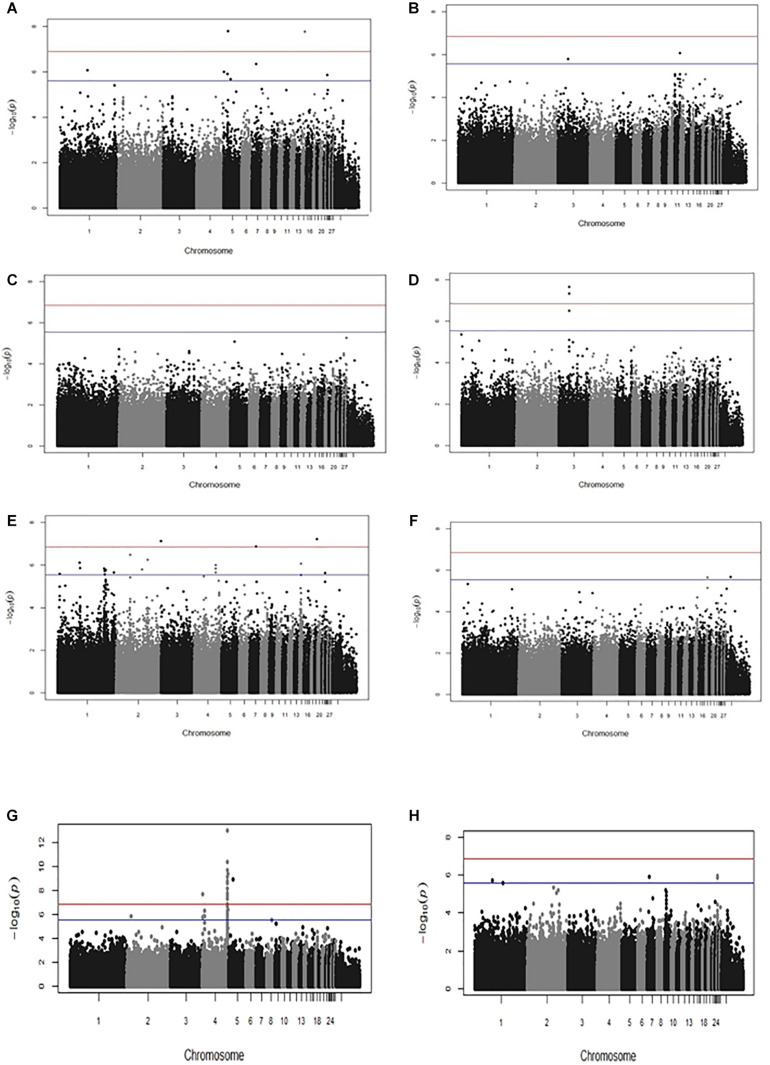
Manhattan plots displaying the results of the genome-wide association analyses performed across Horro and Jarso chicken ecotypes. Genomic location (horizontal axis) is plotted against –log_10_(*P*-value); significant and suggestive significant genome-wide thresholds are shown as red and blue lines, respectively. Manhattan plots are for: **(A)** Infectious Bursal Disease virus (IBDV) antibody titer, **(B)** Marek’s Disease virus (MDV) antibody titer, **(C)** *Pasteurella multocida* (PM) antibody titer, **(D)** *Salmonella enterica* serovar *Gallinarum* (SG) antibody titer, **(E)** Cestode parasitism, **(F)** *Eimeria* parasitism, **(G)** Live body weight (BW), **(H)** Body condition score (BCS).

Genome-wide association studies outcomes revealed several new significant and suggestive genome-wide SNP associations for all the traits with the exception of PM antibody response (Figure 1 and Supplementary Figure S1, Supplementary Table S1). We identified novel significant SNPs for IBDV (*P*-values 1.73 × 10^–8^ to 1.63 × 10^–8^) and SG (4.68 × 10^–8^ to 2.23 × 10^–8^) antibody titers as well as cestodes parasitism (1.35 × 10^–7^ to 5.88 × 10^–8^). Moreover, suggestive genome-wide associations were identified for MDV antibody response and *Eimeria* parasitic resistance. A very strong genome-wide significant association was identified for BW (5.32 × 10^–8^ to 9.87 × 10^–14^) with SNP markers on chromosome 4. The same genomic association with BW had been previously reported separately within the Horro and Jarso ecotypes (Psifidi et al., 2016). Moreover, 22% of the SNPs identified in the present across-ecotype analyses had previously been identified in within-ecotype analyses of the same traits (Psifidi et al., 2016). Manhattan plots displaying the significant association results are shown in Figure 1; the corresponding Q-Q plots, which corroborate the genomic association results, are shown in Supplementary Figure S1. The list of all SNP markers with a significant or suggestive genome-wide association with the studied traits is presented in Supplementary Table S1.

The candidate regions identified by the GWAS for each of these traits contained a limited number of protein-coding genes (*n* = 180) and non-coding RNAs (*n* = 14), summarized in Supplementary Table S2.

### Whole Genome Sequencing Analysis

Whole-genome sequencing analysis revealed the presence of high and/or moderate impact genetic variants located within the candidate regions for all studied traits. The majority of the identified genetic variants were common to both Horro and Jarso ecotypes (Table 4). Nevertheless, some unique genetic variants for each of the two ecotypes were also identified (Table 4). Genetic variants of interest identified in several immune related genes for all the antibody response and parasitic infection traits: IBDV (*TOLLIP, ANGPTL5, BCL9, THEMIS2*), MDV (*GRM7*), SG (*MAP3K21*), *Eimeria* (*TOM1L1*), and cestodes (*TNFAIP1, ATG9A, NOS2*). Details of the genetic variants with a high and moderate predicted impact on the encoded protein identified in the candidate regions of the studied traits are presented in the Supplementary Table S3.

**TABLE 4:** WGS analysis results.

**Genetic variants**	**Traits**							
**Population**	**IBDV**	**MDV**	**PM**	**SG**	**Cestodes**	***Eimeria***	**BW**	**BCS**
**Unique to Horro birds**								
High impact variants	0	0	0	0	0	0	0	0
Moderate impact variants	46	21	13	5	25	0	0	8
Affected genes	18	6	4	2	14	0	0	4
**Unique to Jarso birds**								
High impact variants	0	0	0	0	1	0	12	3
Moderate impact variants	99	1	16	4	38	16	0	28
Affected genes	40	1	7	2	23	2	2	11
**Present in both Horro and Jarso birds**								
High impact variants	5	0	1	0	2	0	0	1
Moderate impact variants	290	2	87	0	169	31	107	116
Affected genes	57	2	9	0	30	3	16	22

*Genetic variants with a predicted high and moderate impact identified in the candidate regions for each of the studied traits in Horro and Jarso chickens. IBDV, titres measured for Infectious Bursal Disease virus; MDV, antibody titres measured for Marek’s Disease virus; SG, antibody titres measured for Salmonella enterica serovar Gallinarum; PM, antibody titres measured for Pasteurella multocida; Eimeria, resistance to Eimeria parasitism; Cestodes, resistance to cestodes parasitism; BW, live body weight; BCS, body condition score. High impact: predicted high impact variant by Ensembl Variant Effect Predictor tool; Moderate impact: predicted moderate impact variant by Ensembl Variant Effect Predictor tool; Affected genes: number of genes containing one or more predicted high or moderate impact variant within the 200 kb window around the significant SNP marker identified by the GWAS analyses.*

### Pathway and Network Analysis

**FIGURE 2:**
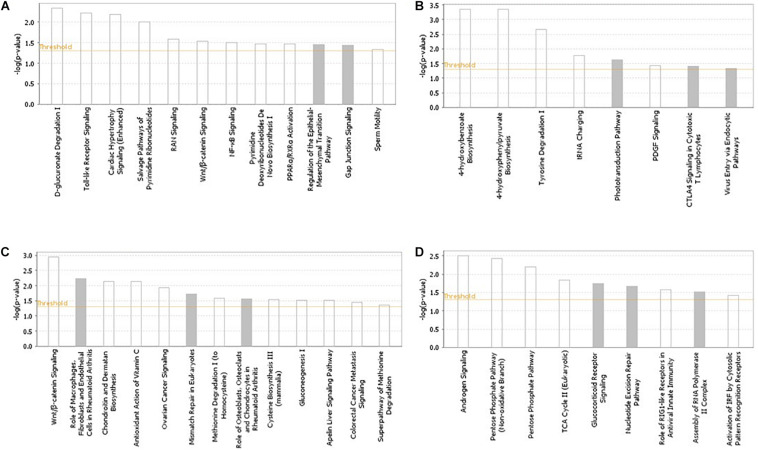
Pathways analysis results using the IPA software across Horro and Jarso chicken ecotypes. The most highly represented canonical pathways of genes located in the candidate genomic regions for **(A)** Infectious Bursal Disease virus (IBDV) antibody titer, **(B)** Marek’s Disease virus (MDV) antibody titer, **(C)** Cestodes parasitism resistance, **(D)** Live body weight. The solid yellow line represents the significance threshold. The line with squares represents the ratio of the genes within each pathway to the total number of genes in the pathway.

The pathways and networks constructed from the gene products located in the candidate regions (based on the across-ecotype GWAS analyses) for each of the studied traits are presented in Figure 2 and Supplementary Figure S2, respectively. Enriched pathways for IBDV and MDV antibody responses, and for cestodes parasitic resistance were mainly related to innate and adaptive immune responses (Figure 2). Examples include NF-Kb, toll-like receptor, interferon, B-cell response, T-cell, and Wnt/β-catenin signaling (Figure 2). In accordance with the previous within-ecotype analysis (Psifidi et al., 2016), the enriched pathways for BW were related to androgen signaling and pentose phosphate (Figure 2), which are associated with gluconeogenesis processes.

Moreover, several networks of molecular interactions were constructed using the list of genes in the candidate regions for antibody response to IBDV, MDV and SG, as well as resistance to parasitic infections (Supplementary Figure S2). The networks associated with resistance to parasitic infection were mostly related to cell death and survival, cell to cell signaling and interaction, immune trafficking, and hematological and immunological diseases (Supplementary Figure S2). For BCS, a network related to cellular function and maintenance as well as skeletal and muscular system development and function was constructed (Supplementary Figure S2).

### Estimation of Genomic Breeding Values and Genomic Predictions

Reliability estimates of GEBVs and accuracy of genomic predictions from the across- and within-ecotype analyses are summarized in Table 5. Across-ecotype analysis resulted in a moderate to high GEBV reliability (0.37–0.80) for all traits with a statistically significant heritability (Table 1). The joint analysis of the two ecotypes improved the GEBV reliability of all traits considerably, compared to within-ecotype analysis (Table 5). On the other hand, genomic prediction accuracies from the across-ecotype analyses were not higher compared to within-ecotype. In the latter case, predictions were more accurate within the Horro ecotype, probably due to the higher heritability of the traits compared to Jarso. Interestingly, for some traits, such as IBDV antibody titer and parasitic infection resistance, within-ecotype predictions were not attainable and an across-ecotype analysis was the only option.

**TABLE 5:** Reliability of genomic breeding values (GEBV) and cross-validation accuracy of genomic predictions from within- and across-ecotype analyses.

	**IBDV**	**MDV**	**SG**	**PM**	***Eimeria***	**Cestodes**	**BW**	**BCS**
**Within-ecotype Horro**								
GEBV Reliability	Non-estimable	0.54	Non-estimable	0.39	Non-estimable	Non-estimable	Not estimable	Non-estimable
Accuracy	Non-estimable	0.40	Non-estimable	0.36	Non-estimable	Non-estimable	0.39	Non-estimable
Relative Accuracy	Non-estimable	0.66	Non-estimable	0.57	Non-estimable	Non-estimable	0.53	Non-estimable
**Within-ecotype Jarso**								
GEBV Reliability	Non-estimable	0.21	Non-estimable	Non-estimable	0.16	0.58	0.07	0.25
Accuracy	0.13	0.27	Non-estimable	Non-estimable	0.18	0.20	0.29	0.17
Relative Accuracy	0.24	0.39	Non-estimable	Non-estimable	0.26	0.31	0.44	0.26
**Across-ecotype**								
GEBV Reliability	0.37	0.55	Non-estimable	0.80	0.76	0.80	0.45	Non-estimable
Accuracy	0.17	0.20	Non-estimable	0.16	0.10	0.16	0.23	Non-estimable
Relative Accuracy	0.24	0.32	Non-estimable	0.34	0.22	0.37	0.34	Non-estimable

*IBDV, antibody titers to Infectious Bursal Disease virus; MDV, antibody titers to Marek’s Disease virus; SG, antibody titers to *Salmonella enterica serovar Gallinarum*; PM, antibody titers to *Pasteurella multocida*; *Eimeria*, resistance to *Eimeria* parasitism; Cestodes, resistance to cestodes parasitism; BW, live bodyweight; BCS, body condition score; Relative Accuracy, Accuracy corrected by the square root of heritability. Non-estimable results signified failure of the calculating algorithm to converge.*

## Discussion

The present study set out to investigate the possibility of combining across-ecotype chicken data to study the genomic architecture of health and productivity traits, and examine the possibility of genomic selection in Ethiopian indigenous chickens. The GWAS analyses of the combined bird ecotype data revealed the presence of multiple novel genomic regions significantly associated with the traits of interest. This may be an indication that the two populations may have shared a common genetic profile in the recent history and confirmed that a larger sample size increases the power of genomic detection. Genomic and WGS information were integrated in order to detect and prioritize candidate genes, genetic variants and underlying pathways and networks for antibody response to bacterial and viral infections, resistance to parasitic infections and production traits. Moreover, the current study explored, for the first time to our knowledge, the possibility of performing genomic selection across distinct indigenous African chicken ecotypes and demonstrated the scope for merging such data in genetic improvement programs.

The across-ecotype population heritability estimates fell within the range of those previously calculated separately for the Horro and Jarso ecotypes (Psifidi et al., 2016). Most studied traits, except for BCS and SG, were heritable and amenable to improvement with genetic selection. Importantly, BCS and SG were found to be genetically correlated with BW and PM, respectively, which are highly heritable traits and, therefore, the former would indirectly benefit from genetic selection and improvement in the latter. Moreover, the *Eimeria* resistance heritability in Horro birds, which was not previously estimable within-ecotype (Psifidi et al., 2016), was estimable and significant when calculated in the across-ecotype analysis in the present study. This suggests that, despite the genetically distinct profiles of the two ecotypes (Psifidi et al., 2016), there is sufficient shared genetic material to allow for an across-ecotype analysis of Horro and Jarso indigenous birds. These findings are in accordance with a previous study comparing WGS data from Horro and Jarso birds to Saudi Arabian (AlQurin, Goliggah, and Al Oyoun districts) and Sri Lankan (Puttalam district) indigenous chickens and red junglefowl (Yunnan and Hainan provinces, China) aiming to explore genetic differentiation between the populations (Lawal et al., 2018). According to the latter, both Ethiopian ecotypes clustered together distinctly from the other indigenous birds and the junglefowl, indicating closer genetic relationships between these ecotypes. Whilst there was some separation between Horro and Jarso within the Ethiopian cluster, these results supported analyses of an across-ecotypes population, particularly in combination with selective sweep results indicating different selection pressures in Ethiopian birds from the other indigenous birds and red junglefowl (Lawal et al., 2018).

Phenotypic and genetic correlations between traits were generally consistent with previous reports from within-ecotype analyses (Psifidi et al., 2016). However, the genetic correlation between resistance to the two parasitic infections (*Eimeria* and cestodes) was strongly negative (−0.63) in the combined population compared to a positive estimate in Horro (0.68) and a zero estimate in Jarso in previous within-ecotype analyses (Psifidi et al., 2016). *Eimeria* co-infection responses are complex and dependent on the nature of the co-infection. Co-infection of chickens with *Eimeria* and *Toxoplasma gondii* in a previous experimental study (Hiob et al., 2017) demonstrated no significant difference in pathology or immune response to *Eimeria* infection alone; however, *Eimeria* oocyst excretion was actually lower in the co-infected birds than mono-infected (Hiob et al., 2017). This could potentially explain the negative phenotypic and genetic correlation between *Eimeria* and cestode oocysts burden estimated in the present study. However, new studies based on controlled challenge experiments are needed in order to confirm these findings. The other phenotypic and genetic correlation results support previous observations of PM and SG co-infections (Biswas et al., 2005) as well as IBDV and SG co-infections (Chaka et al., 2012) in village poultry. In accordance with previous studies (Psifidi et al., 2016), *Eimeria* parasitism displayed negative phenotypic correlations with PM and SG antibody responses that might be suggestive of parasitic immunosuppression.

Importantly, there were no significant genetic correlations of production traits with antibody response and resistance to parasitic infection, in agreement with the previous within-ecotype analyses (Psifidi et al., 2016). This result would greatly facilitate the development of concomitant breeding programs aiming to improve simultaneously production and disease resistance traits, which would have been hampered by potentially antagonistic correlations between traits.

Genomic analyses based on joined data of the two ecotypes combined with whole genome sequencing analysis identified several novel significant SNP markers, genes of interest and genetic variants, which are suitable candidates for further investigation to determine if they can be exploited in future breeding programs. About one fifth (22%) of the SNPs identified in the across-ecotype analysis here had previously been identified in the separate analyses of the two ecotypes (Psifidi et al., 2016), attesting to a partially common genetic basis of these traits in the two populations. Interestingly, some of the SNP associations previously identified in the within-ecotype analyses (Psifidi et al., 2016) now attained an even higher significance, suggesting that the bigger sample size in the joint analysis increased the GWAS power to not only identify novel associations but also confirm previously identified ones.

Similarly, pathway analysis not only confirmed the presence of previously identified pathways but also identified enrichment for several immune pathways, which had not been detectable in the previous within-ecotype analyses (Psifidi et al., 2016). For the IBDV antibody responses among the novel identified pathways was the toll-like receptor (TLR) signaling, which has been previously linked to IBDV. *TLR3* has been found to be up-regulated in IBDV-infected chickens (Ou et al., 2017), and the function of this TLR has been considered vital to innate responses to viral infection (Yang et al., 2016). We also identified two missense variants in the toll-interacting protein (*TOLLIP*) gene, which is a component of the TLR signaling pathway that acts to inhibit cell activation, and five missense variants in the single immunoglobulin IL-1R-related molecule (*SIGIRR*), which is a TLR/IL-1 receptor system antagonist. One novel pathway found here to be associated with MDV antibody titer was cytotoxic T lymphocyte (CTL) signaling. MDV is a lymphotropic virus and has previously been associated with lowered ratio of CTLs to CD4^+^ T cells in infected chicken skin (Heidari et al., 2016) due to immunosuppression. A comparison of MDV resistant and susceptible chicken lines demonstrated that baseline levels of specifically CD8αα T cells were different between the two lines, indicating that these cells play an important role in effective immune response to MDV infection (Perumbakkam et al., 2016).

Furthermore, cestode resistance was associated with Wnt/β-catenin signaling and with networks linked to digestive system development and function, and with cellular morphology and abnormalities. Interestingly, cestode infections can be treated with anthelmintic drugs such as niclosamide (Katz, 1977), mebendazole or pyrvinium (Hamilton and Rath, 2017), which are also used to treat Wnt-dependent cancers (Lu et al., 2011; Osada et al., 2011; Zhang et al., 2017) due to their Wnt inhibitory action (Chen et al., 2009). These results suggest a potential involvement of the Wnt signaling pathway to cestode parasitism resistance.

Live body weight was unsurprisingly associated with androgen signaling, which has long been known to affect body growth (Li et al., 2020). In addition, two missense variants were identified in the adipose triglyceride lipase (*ATPL/PNPLA2*) gene that has been previously associated with chicken growth and fat deposition traits (Fennell and Scanes, 1992; Fennell et al., 1996; Nie et al., 2010). Moreover, previously identified polymorphisms in this gene have been associated with myopathic phenotypes (Akiyama et al., 2007; Fischer et al., 2007). The majority of high and moderate predicted impact variants were present in both ecotypes and all studied traits, with the notable exception of BW where 12 predicted high impact variants, all of them located in two novel long non-coding RNAs (lncRNAs), were found uniquely in Jarso chickens, with no shared high impact variants found in Horro chickens. There was a significant difference in BW between the two ecotypes previously (Psifidi et al., 2016) [unpaired two-tailed *t*-test; *t*(758) = 5.747; *P* < 0.0001], with Jarso animals being the smaller ones, which may explain the number of predicted high impact variants found only in Jarso birds in candidate regions linked to live body weight and growth. This suggestion is in accordance with previous studies of humans, mice and cattle, which have reported that many lncRNAs play crucial roles in the development of skeletal muscle and cardiac lineages (Sweta et al., 2019). Furthermore, the QTLs identified for BW in the Ethiopian ecotypes are quite unique. A search in the Chicken QTL database^2^ identified 337 genomic associations across chicken chromosomes 2, 4 and 5 for BW, from a total of 69 different studies. However, none of these were within 1 Mb of genomic markers identified in the present study. All the above findings warrant further investigation in order to validate and fully dissect the underlying molecular mechanisms connecting genotypes with disease and productivity phenotypes, and determine how to best inform future breeding programs.

An advantage of using genomic predictions for selection to improve traits of interest compared to using more targeted approaches is that the effects of all genetic markers across the genome are considered simultaneously. This allows potentially modest additive effects of individual SNPs that may not meet stringent statistical tests to be modeled into phenotypic predictions of complex traits. Furthermore, the identification of causative genes and mutations could increase the accuracy of the estimated genomic predictions (van Binsbergen et al., 2015; Edwards et al., 2016). In the present study, genomic analyses across-ecotype increased the reliability of individual bird GEBVs compared to within-ecotype analyses, likely due to the larger sample size. Moreover, the across-ecotype analyses allowed relatively accurate genomic predictions for traits that could not be attained within-ecotype for this dataset. However, for most traits the accuracy of predictions did not improve across-ecotype compared to within-ecotype suggesting that the linkage disequilibrium between SNPs and QTL does not always persist across the two populations. These findings corroborate the GWAS results showing a partial overlap of genomic associations within and across ecotypes. Moreover, the cross-validation method considered in the present study required approximately equal proportions of Jarso and Horro birds within each subset in order to improve the balance in prediction across ecotypes. Nevertheless, methods of genomic predictions should be consistent with the actual aims of genomic selection; for example, if the cross-region movement of birds is not an option, then genomic predictions should be weighed toward the ecotypes that are more common in the specific geographic region. An alternative approach to cross-validation in assessing the accuracy of genomic predictions would be forward validation, which makes use of data from earlier years to train the model and predict performance in the recent years. Since it uses all available data, forward validation might expedite genomic selection response. This approach has previously been successfully applied in studies of indigenous cattle breeds (Neves et al., 2014; Silva et al., 2016; Boison et al., 2017) but not of chicken.

Results obtained in the present study apply to the two Ethiopian ecotypes but the principle of across-ecotype genomic selection may be expanded to other chicken populations raised in sub-Saharan Africa provided that the phenotypes of interest and the associated markers are to some extend common in different populations. Size of available phenotypic and genomic data, and the genetic similarity of the ecotypes would be the key determinants of genomic selection success in each case. Therefore, further studies aiming to genetically and phenotypically characterize other indigenous chicken ecotypes are required to inform and underpin future breeding programs. In addition, independent studies should establish the required minimum sample size to derive reliable GEBVs and accurate genomic predictions in these settings.

## Data Availability Statement

The Horro and Jarso whole genome sequences used in the present study are deposited in the NCBI Sequence Read Archive (SRA) under accession number SRP142580.

## Ethics Statement

All work was conducted with the approval of the University of Liverpool Research Ethics Committee (reference RETH000410).

## Author Contributions

RC, OH, PW, PK, and TD conceived the experimental design and secured funding. AP, OH, and GB designed the genetic studies. TTD and JB performed the collection of samples and the phenotyping. GB, VL, and AP with input from ESM and OM performed the genetic parameter and the genomic prediction analyses. GB and AP collated and edited the genotyping data and performed the GWAS analysis. AVT and OH generated the WGS data and VL with input from AP analyzed the genetic variants. AP performed the pathway analysis. AP, OH, RC, PW, TD, GB, VL, and ESM interpreted these results. GB and VL wrote the manuscript with input from AP. All other co-authors provided manuscript editing and feedback and read and approved the final manuscript.

## Conflict of Interest

The authors declare that the research was conducted in the absence of any commercial or financial relationships that could be construed as a potential conflict of interest.

## Footnotes

1 http://www.ensembl.org/biomart/martview/

2 https://www.animalgenome.org/cgi-bin/QTLdb/GG/
