# Supplementary material for: Integrating Genetic and Genomic Analyses of Combined Health Data Across Ecotypes to Improve Disease Resistance in Indigenous African Chickens
Source: Front Genet. 2020 Oct 9;11:543890. doi: 10.3389/fgene.2020.543890 (PMC7581896; doi:10.3389/fgene.2020.543890)
Supplement: Supplementary file 1 [file Image_1.pdf]

Supplementary Figure S1. Q–Q plots displaying the GWAS results across-ecotype.

Observed P-values are plotted against the expected P-values for **a)** Infectious bursal disease virus (IBDV) antibody titre; **b)** Marek's disease virus (MDV) antibody titre; **c)** *Salmonella enterica* serovan Galinarum (SG) antibody titre; **d)** *Eimeria* parasitism resistance; **e)** cestodes parasitism resistance; **f)** body condition score (BCS); **g)** live body weight (BW)

a) IBDV

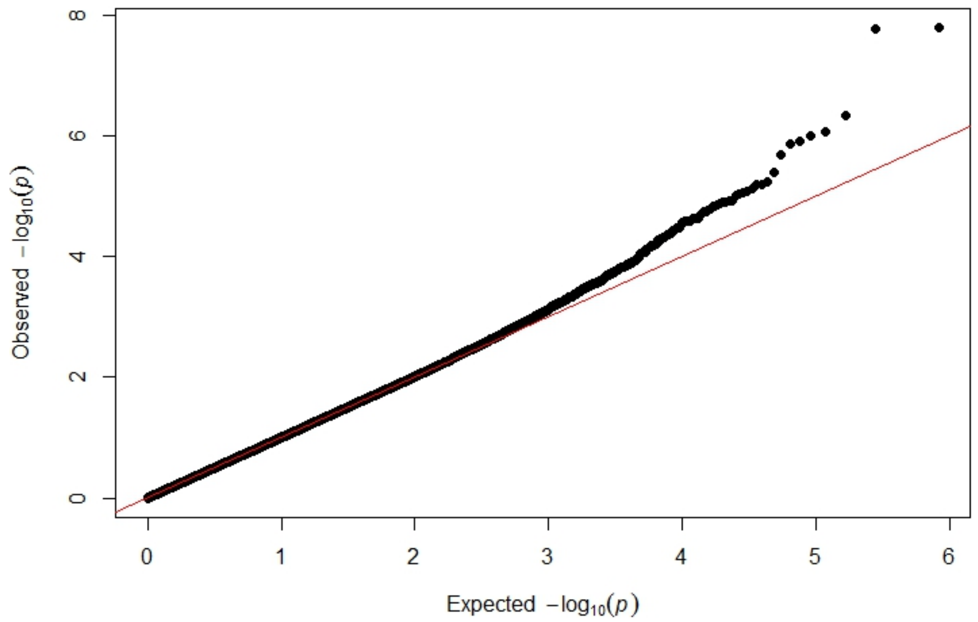

b) MDV

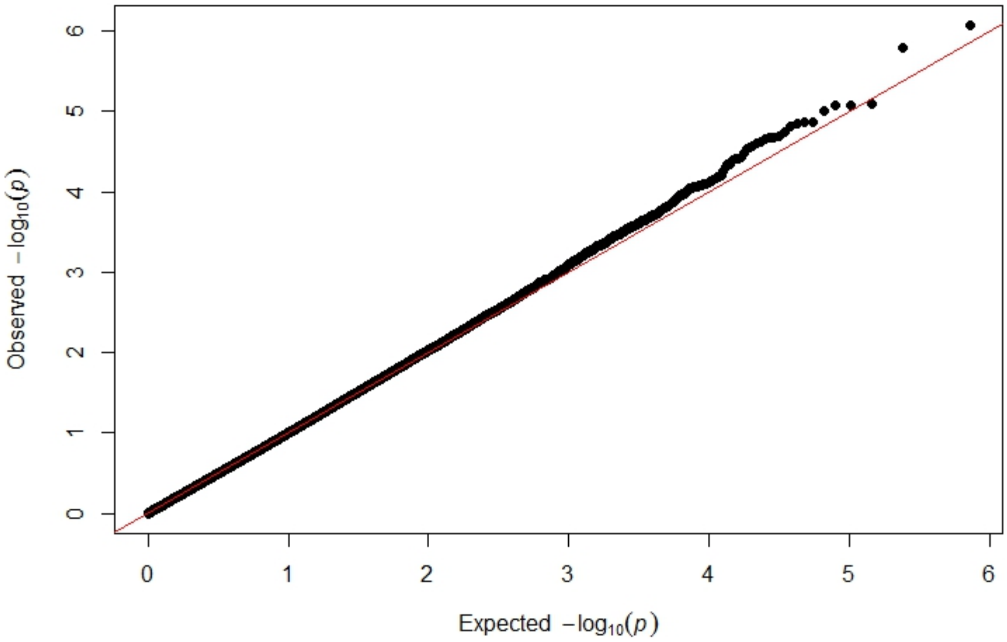

Observed −log10(p)
Expected −log10(p)

c) SG

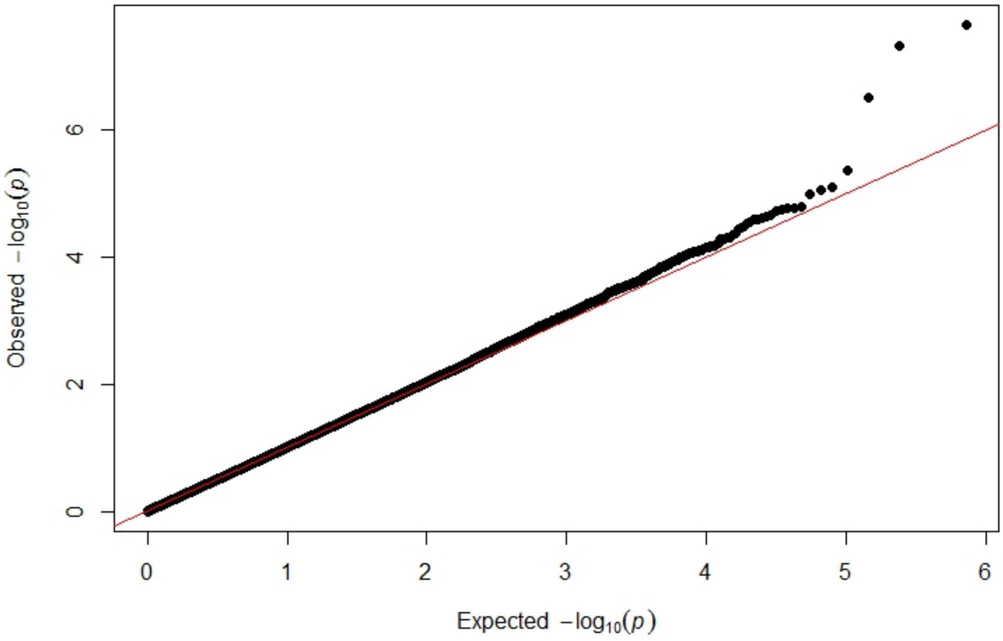

Observed −log10(p)
Expected −log10(p)

d) *Eimeria*

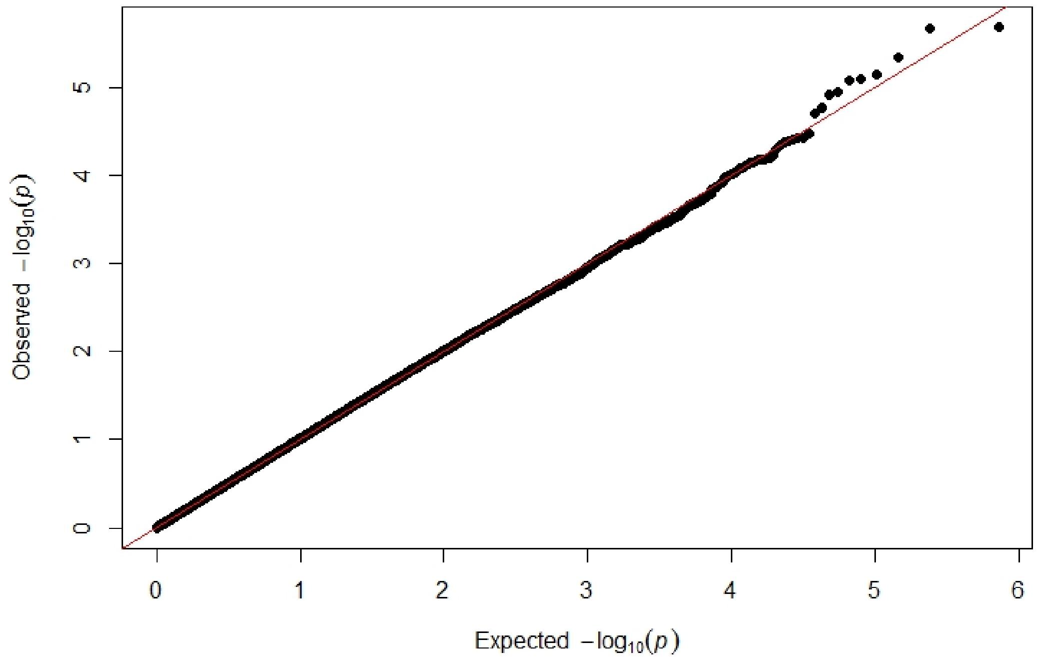

e) Cestodes

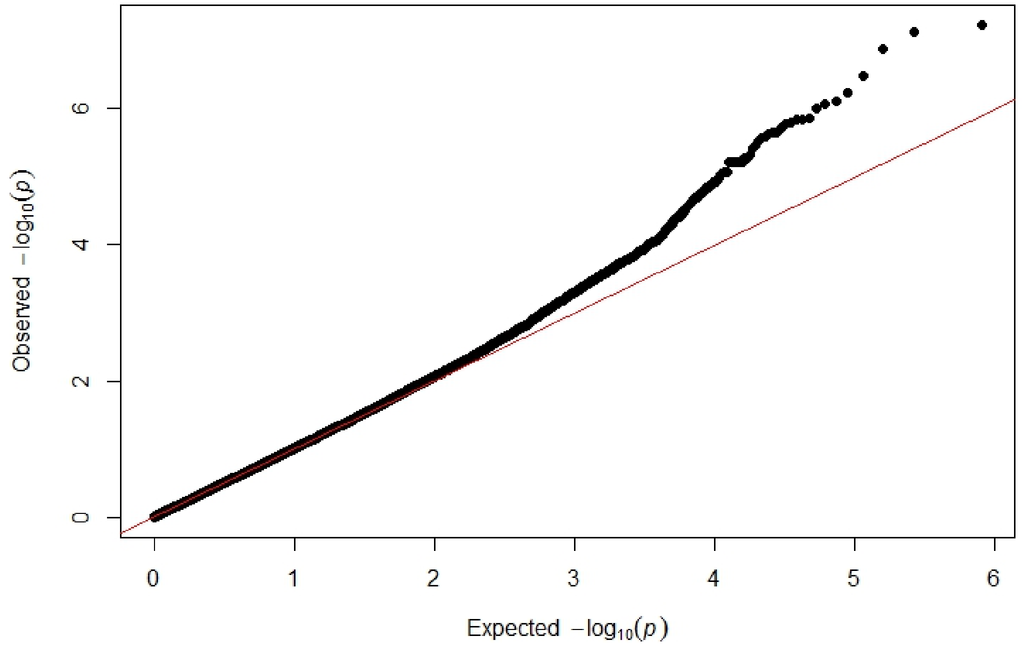

f) BCS

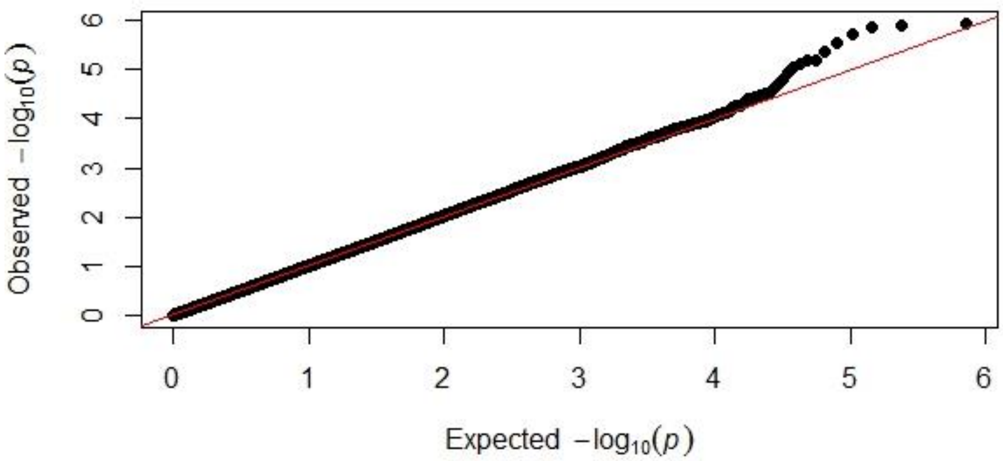

Observed $-\log_{10}(p)$
Expected $-\log_{10}(p)$
0
1
2
3
4
5
6

g) BW

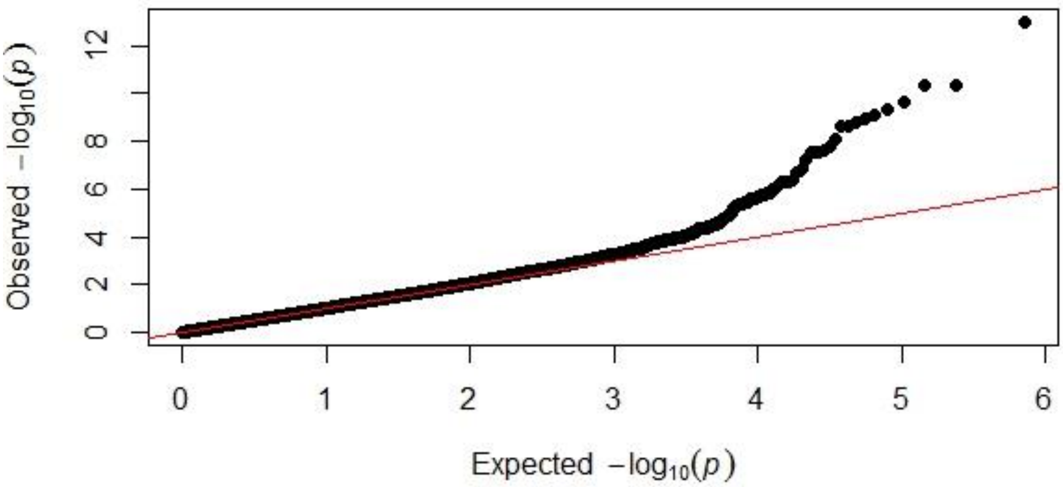

Observed $-\log_{10}(p)$
Expected $-\log_{10}(p)$
0
2
4
6
8
12
0
1
2
3
4
5
6
