# Supplementary material for: Integrating Genetic and Genomic Analyses of Combined Health Data Across Ecotypes to Improve Disease Resistance in Indigenous African Chickens
Source: Front Genet. 2020 Oct 9;11:543890. doi: 10.3389/fgene.2020.543890 (PMC7581896; doi:10.3389/fgene.2020.543890)

Supplementary Figure S2. Network analysis results using IPA software across ecotypes. The networks illustrate the molecular interactions between products of genes selected from candidate genomic regions for Infectious bursal disease virus (IBDV) antibody titre (Network A and B); Mareks' disease virus (MDV) antibody titre (C and D); *Salmonella enterica* serovar Galinarum (SG) antibody titre (E); *Eimeria* parasitism resistance (F and G); cestodes parasitism resistance (H and I). Solid and dashed arrows represent direct and indirect interactions, respectively. The white colour indicates gene products added to the IPA analysis because of their interaction with the target gene products.

A) Network related to cell to cell signalling and interactions, hematological system develoment and interaction and immune cell trafficking illustrates molecular interactions between products of candidate genes selected from the candidate regions for antibody titres to Infectious Bursal Disease Virus (IBDV)

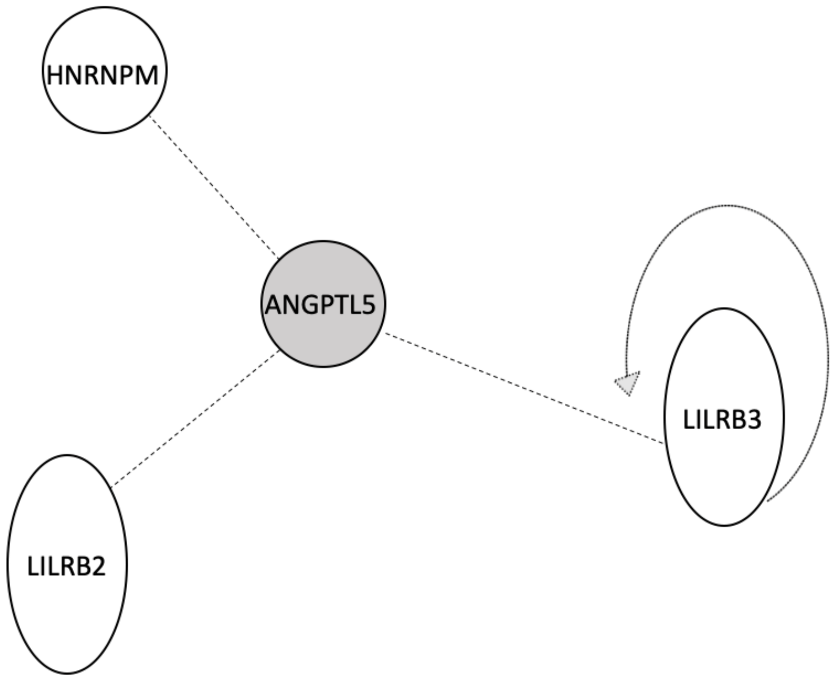

B) Network related to cellular assembly and maintenance, cellular functions, molecular transport and cell signalling and interaction illustrates molecular interactions between products of candidate genes selected from the candidate regions for antibody titres to Infectious Bursal Disease Virus (IBDV)

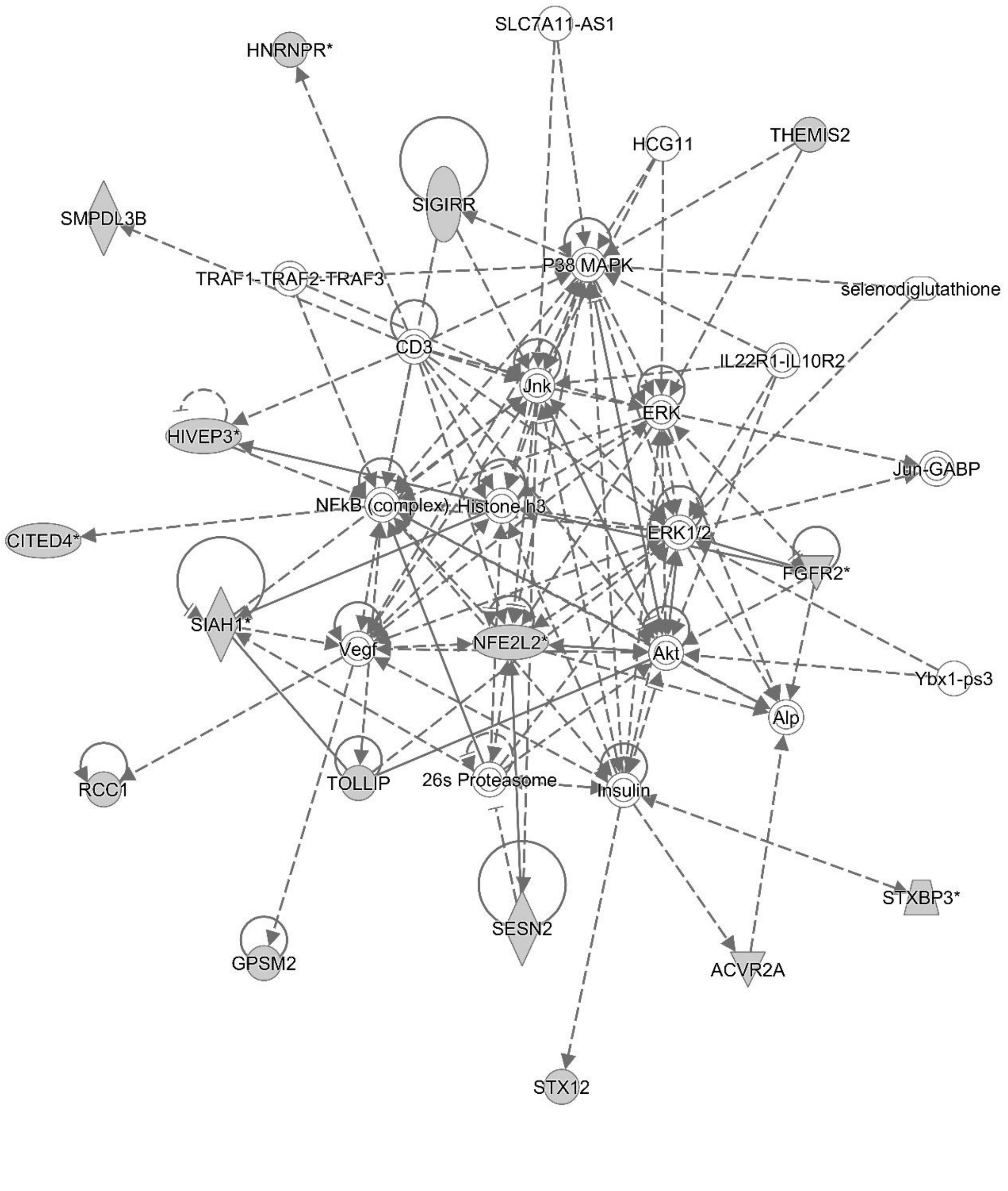

C) Network related to antimicrobial response, cell death and survival and cell signalling illustrates molecular interactions between products of candidate genes selected from the candidate regions for antibody titres to Marek's disease virus (MDV)

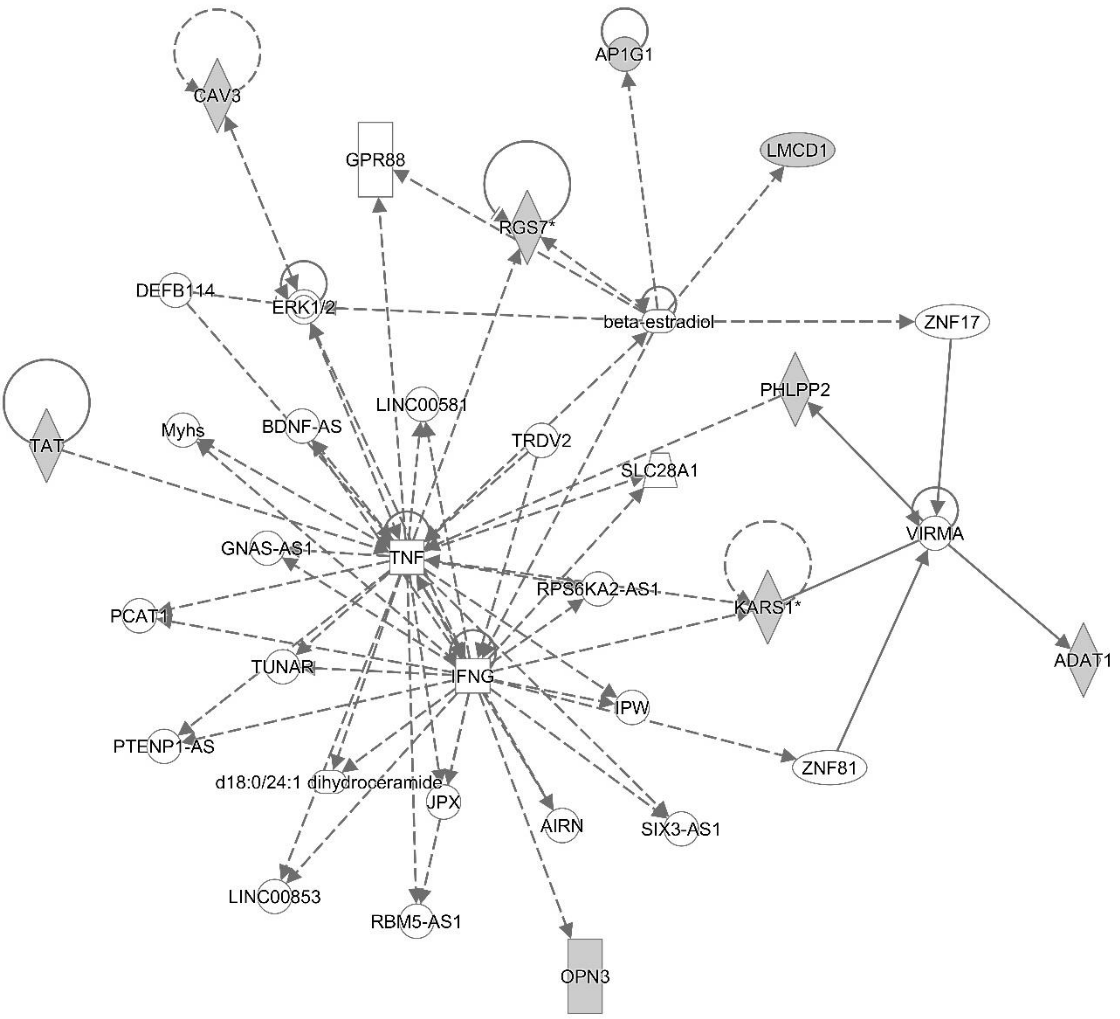

D) Network related to cancer, hematological disease and immunological disease illustrates molecular interactions between products of candidate genes selected from the candidate regions for antibody titres to Marek's disease virus (MDV)

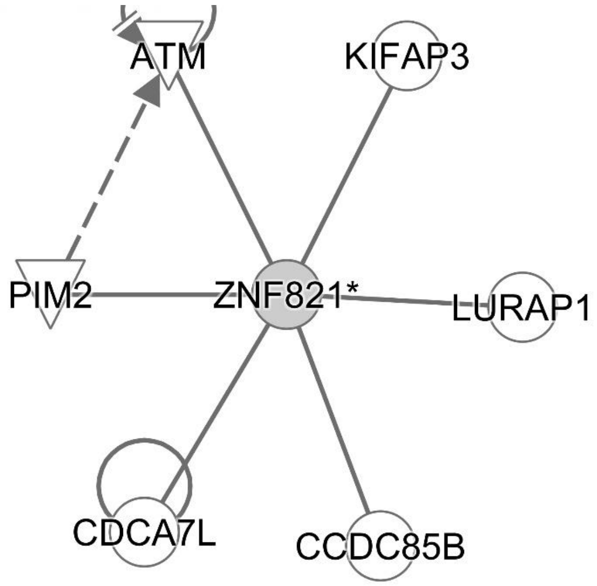

E) Network related to cell cycle, DNA replication, recombination and repair illustrates molecular interactions between products of candidate genes selected from the candidate regions for *Salmonella enterica* serovar Galinarum (SG) antibody titre

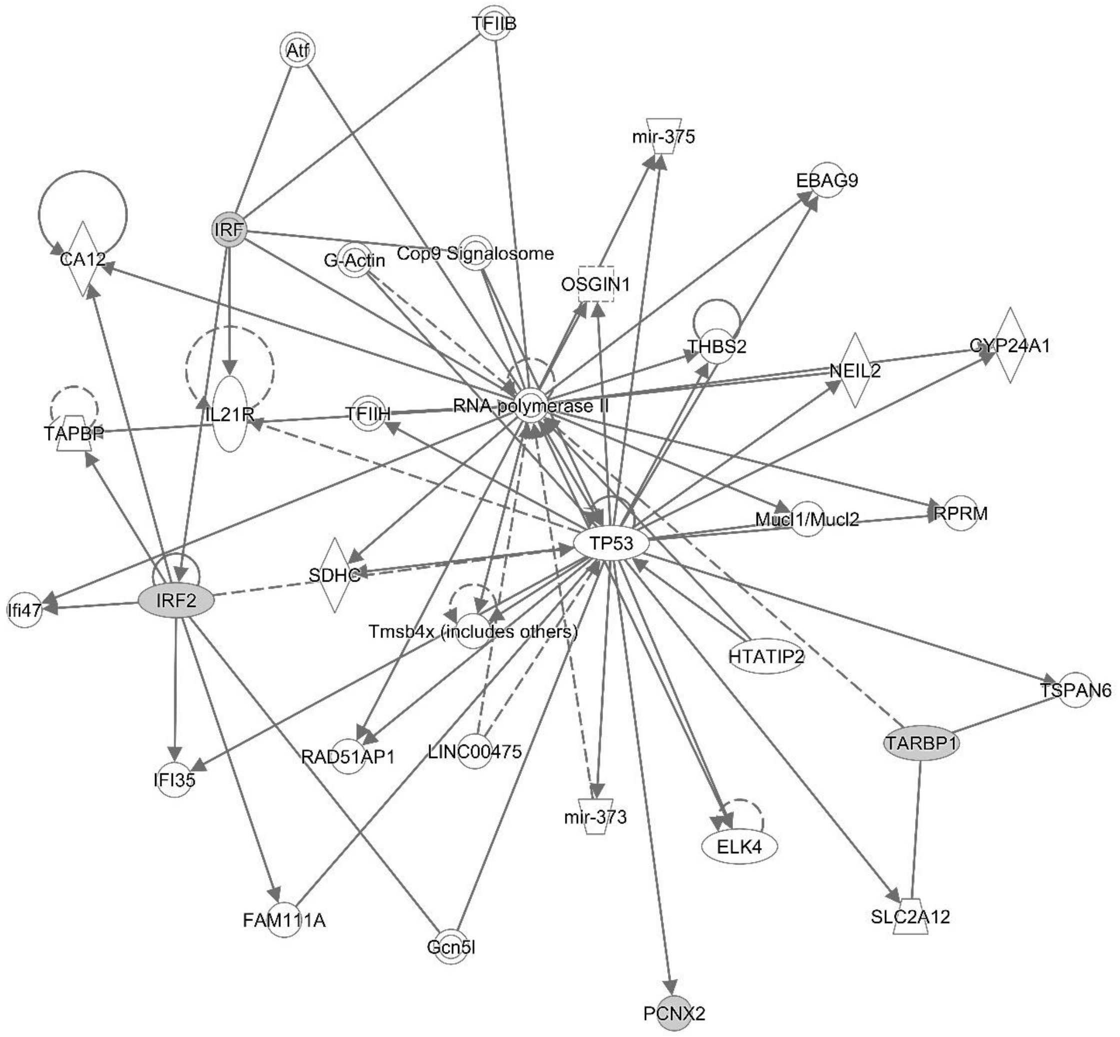

F) Network related to infectious diseases, cellular morphology and organismal development illustrates molecular interactions between products of candidate genes selected from the candidate regions for *Eimeria* parasitism resistance

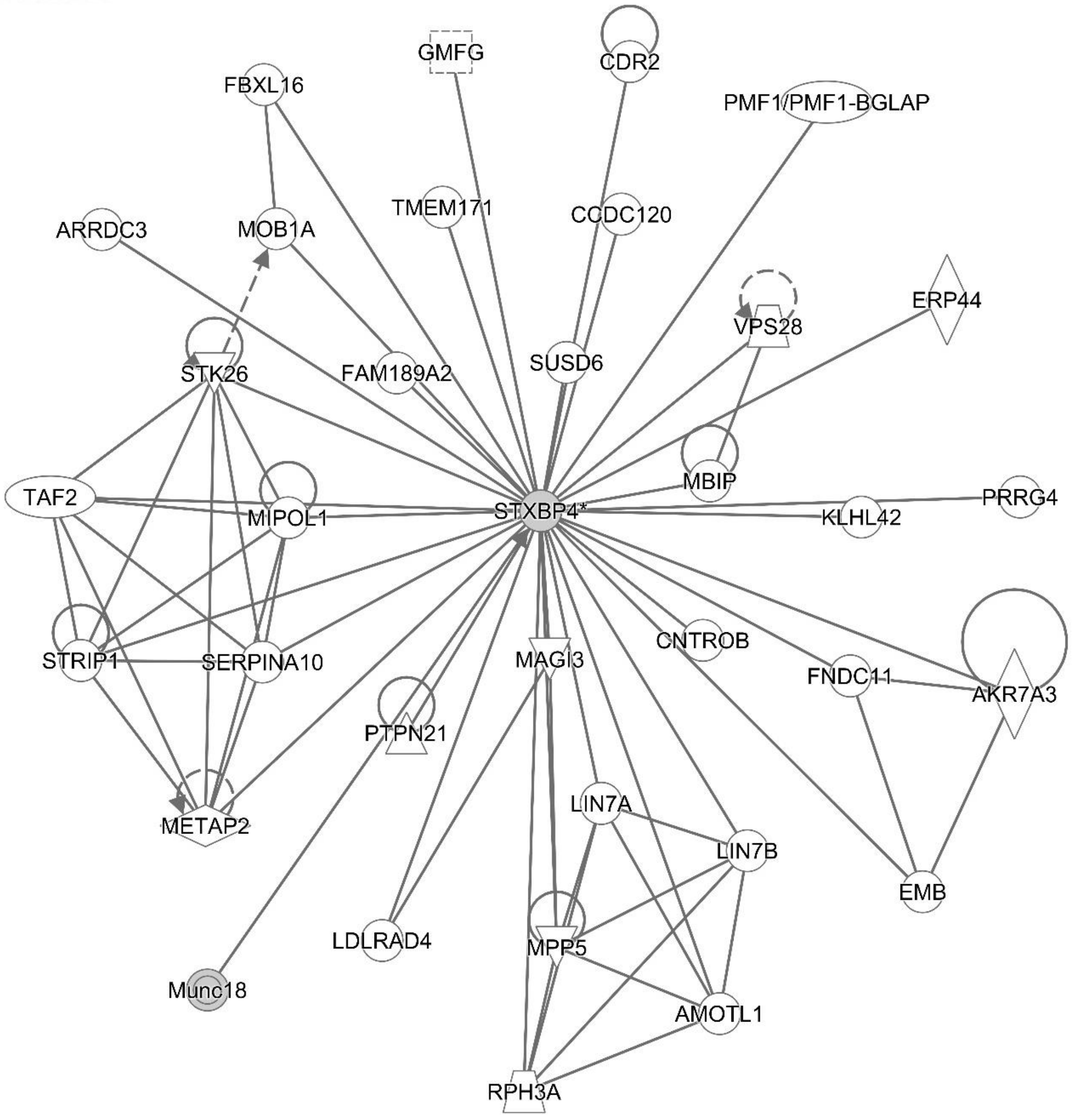

G) Network related to haematological system development and function, lymphoid tissue structure and development and cellular development illustrates molecular interactions between products of candidate genes selected from the candidate regions for *Eimeria* parasitism resistance

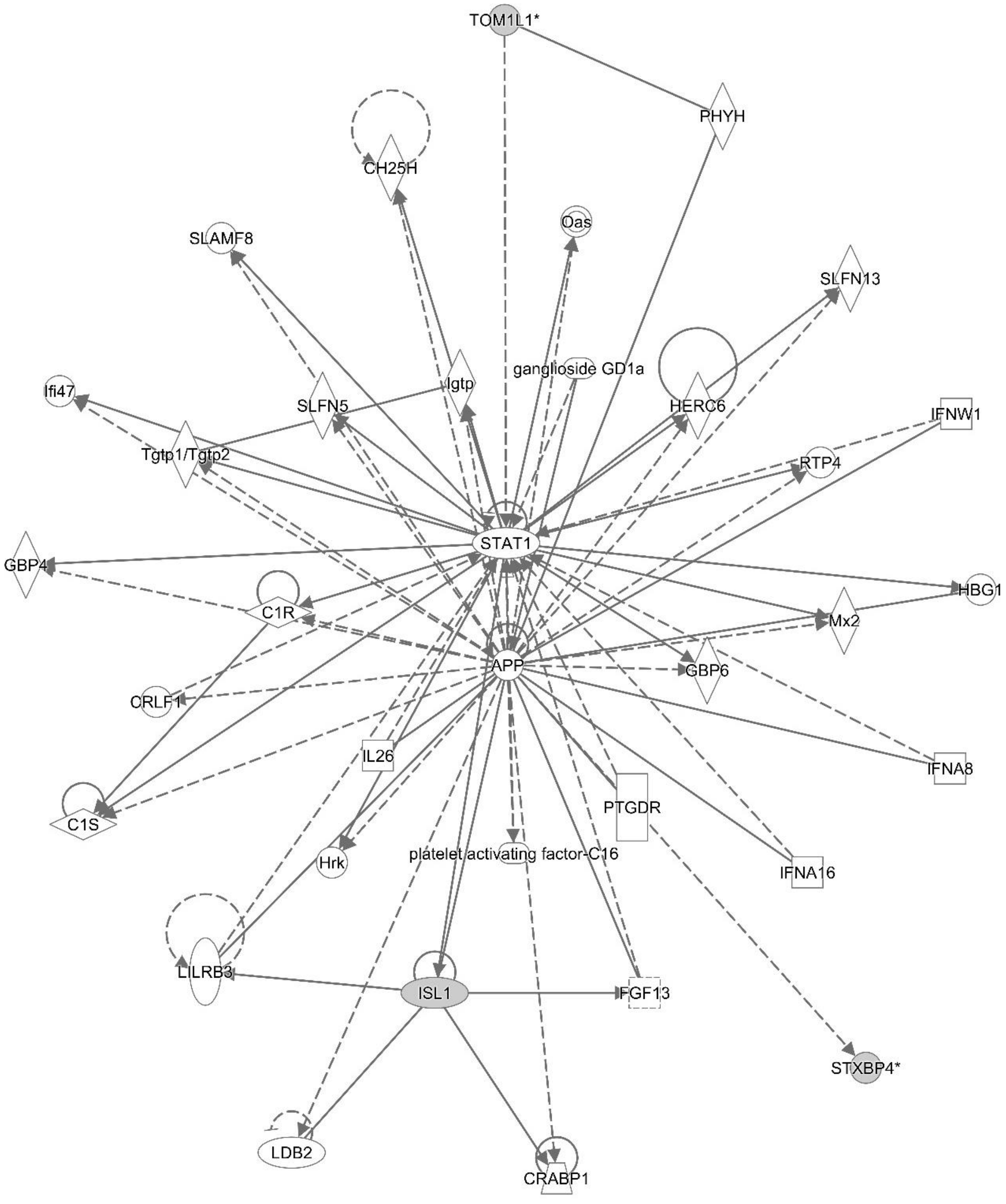

H) Network related to digestive system development and function, cellular growth and cellular growth and proliferation illustrates molecular interactions between products of candidate genes selected from the candidate regions for cestodes parasitism resistance

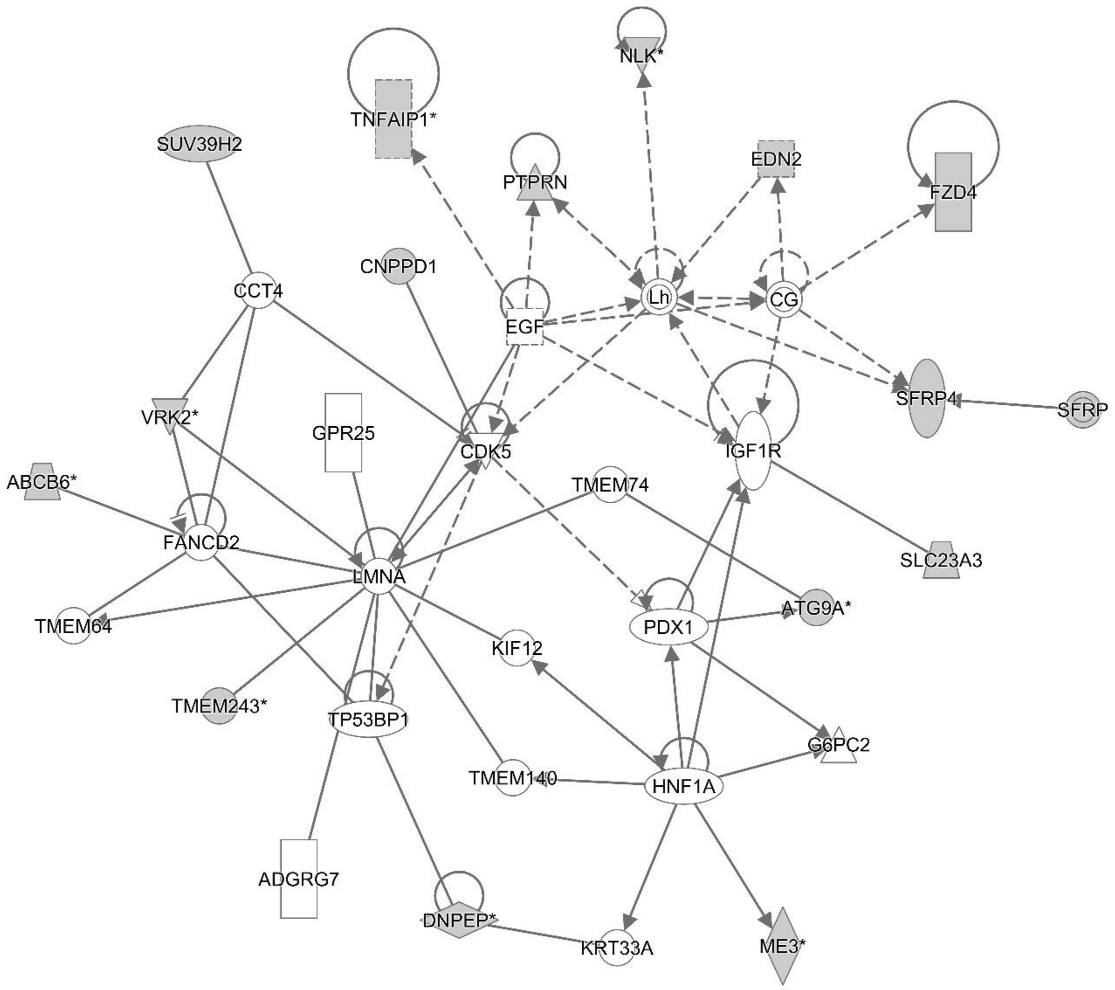

I) Network related to cellular morphology, organismal injury and abnormalities illustrates molecular interactions between products of candidate genes selected from the candidate regions for cestodes parasitism resistance

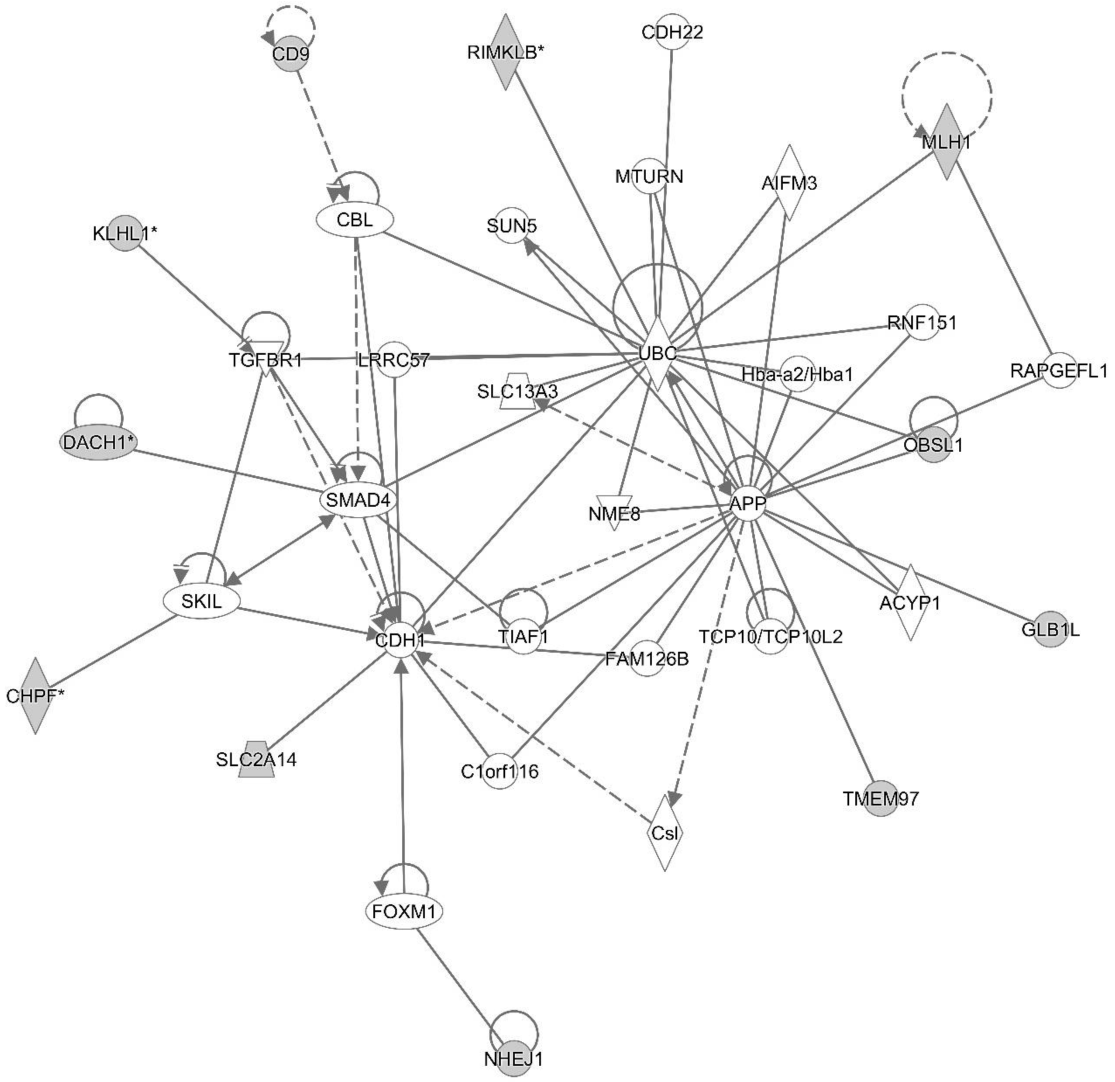

Supplement: Supplementary file 2 [file Image_2.pdf]
