# Supplementary material for: Integrating Genetic and Genomic Analyses of Combined Health Data Across Ecotypes to Improve Disease Resistance in Indigenous African Chickens
Source: Front Genet. 2020 Oct 9;11:543890. doi: 10.3389/fgene.2020.543890 (PMC7581896; doi:10.3389/fgene.2020.543890)
Supplement: Supplementary file 3 [file Table_1.docx]

| **Supplementary Table S1: Significant SNP markers identified for traits across Horro and Jarso ecotype** | | | |
| --- | --- | --- | --- |
| **Traits** | **SNP marker ID** | **Location (Chr:bp)** | **GWAS –log (P-value)** |
|  |  |  |  |
| BW | **Affx-51502208** | **4:87162290** | **13.00559** |
|  | **Affx-51502179** | **4:87149557** | **10.38316** |
|  | **Affx-51502172** | **4:87146841** | **10.3416** |
|  | **Affx-51502191** | **4:87154473** | **9.657695** |
|  | **Affx-51502955** | **4:87546243** | **9.360255** |
|  | **Affx-51502383** | **4:87254896** | **9.137412** |
|  | Affx-51525825 | 5:15144036 | 8.926296 |
|  | **Affx-51502311** | **4:87215318** | **8.765596** |
|  | Affx-51502569 | 4:87355520 | 8.638194 |
|  | **Affx-51502405** | **4:87266160** | **8.637241** |
|  | **Affx-51502367** | **4:87244290** | **8.124582** |
|  | Affx-51501683 | 4:86889310 | 7.764747 |
|  | **Affx-51501414** | **4:784066** | **7.653704** |
|  | Affx-51501703 | 4:86901258 | 7.535384 |
|  | **Affx-51502298** | **4:87208267** | **7.509008** |
|  | **Affx-51502304** | **4:87211838** | **7.509008** |
|  | Affx-51502424 | 4:87276646 | 7.273327 |
| IBDV | Affx-51526157 | 5:15315358 | 7.787077 |
|  | Affx-50644388 | 14:11383191 | 7.762727 |
| Cestodes | **Affx-50796263** | **19:9284997** | **7.230329** |
|  | Affx-51348792 | 3:890431 | 7.124505 |
|  | **Affx-51718143** | **7:21664924** | **6.868485** |
| SG | Affx-51254490 | 3:38077280 | 7.650007 |
|  | Affx-51254527 | 3:38096622 | 7.329413 |
| IBDV, antibody titres to Infectious bursal disease virus; SG, antibody titres to *Salmonella enterica* serovar Gallinarum; BW, bodyweight; Cestodes, cestode parasitism. SNPs in bold were also identified as significant in a previous within-ecotype study (Psifidi et al, 2016). | | | |

| **Suggestive SNPs markers identified for traits across Horro and Jarso ecotype.** | | | |
| --- | --- | --- | --- |
| **Traits** | **SNP marker ID** | **Location (Chr:bp)** | **GWAS (-log P-value)** |
| BW | Affx-51501616 | 4:86854546 | 6.817845 |
|  | Affx-51502637 | 4:87389529 | 6.762614 |
|  | Affx-51500067 | 4:86080600 | 6.655927 |
|  | Affx-51503046 | 4:87597134 | 6.367258 |
|  | Affx-51501341 | 4:86710298 | 6.291819 |
|  | Affx-51500918 | 4:86507874 | 6.285076 |
|  | Affx-51501486 | 4:8593412 | 6.283151 |
|  | Affx-51500157 | 4:86131947 | 6.281041 |
|  | Affx-51501218 | 4:86656487 | 6.259496 |
|  | Affx-51501705 | 4:86902187 | 6.095953 |
|  | Affx-51501010 | 4:86553873 | 5.9576 |
|  | Affx-51500839 | 4:86472816 | 5.952637 |
|  | Affx-51502185 | 4:8629823 | 5.870165 |
|  | Affx-50860117 | 2:13719378 | 5.830792 |
|  | Affx-51449189 | 4:5686346 | 5.803264 |
|  | Affx-51501290 | 4:86690723 | 5.793883 |
|  | **Affx-51501208** | **4:783086** | **5.784751** |
|  | Affx-51502102 | 4:87111718 | 5.755692 |
|  | Affx-51501478 | 4:86781891 | 5.650377 |
|  | Affx-51502153 | 4:87138054 | 5.643502 |
|  | **Affx-51502246** | **4:87182268** | **5.597306** |
|  | Affx-51502336 | 4:87228376 | 5.589318 |
|  | Affx-51501854 | 4:86980173 | 5.587502 |
|  | Affx-51500714 | 4:86409290 | 5.575334 |
| BCS | Affx-51149404 | 26:697126 | 5.937897 |
|  | Affx-51762898 | 7:9166101 | 5.916167 |
|  | Affx-51149428 | 26:702040 | 5.879687 |
|  | Affx-50402535 | 1:66051156 | 5.738318 |
|  | Affx-50144617 | 1:105786983 | 5.559386 |
| IBDV | Affx-51703234 | 7:15222973 | 6.339694 |
|  | Affx-50448664 | 1:91814922 | 6.058056 |
|  | Affx-51526500 | 5:1506111 | 6.006319 |
|  | Affx-51523671 | 5:14065282 | 5.905856 |
|  | Affx-51084536 | 23:1467133 | 5.86444 |
|  | Affx-51544394 | 5:24800714 | 5.686428 |
| MDV | Affx-50538850 | 11:19244318 | 6.066809 |
|  | Affx-51249205 | 3:35228422 | 5.792535 |
| *Eimeria* | Affx-51884160 | Z:15274611 | 5.680608 |
|  | **Affx-50757437** | **18:5763355** | **5.66337** |
| Cestodes | Affx-50928110 | 2:46673466 | 6.487665 |
|  | **Affx-50805630** | **2:104948558** | **6.242323** |
|  | Affx-50417758 | 1:74555017 | 6.107253 |
|  | Affx-50666950 | 14:5736929 | 6.069271 |
|  | Affx-51474646 | 4:72203050 | 6.010217 |
|  | Affx-50418819 | 1:75375440 | 5.861815 |
|  | **Affx-51474665** | **4:72218113** | **5.841174** |
|  | Affx-50236960 | 1:155966210 | 5.837024 |
|  | Affx-50991416 | 2:86897663 | 5.797374 |
|  | Affx-50245141 | 1:161217415 | 5.777454 |
|  | Affx-50238406 | 1:156762695 | 5.716982 |
|  | Affx-51473685 | 4:71586812 | 5.652562 |
|  | Affx-50301505 | 1:188411915 | 5.649784 |
|  | Affx-51082842 | 23:1028171 | 5.638841 |
|  | **Affx-50417651** | **1:7326327** | **5.57994** |
| SG | Affx-51254563 | 3:38119360 | 6.51556 |
| IBDV, antibody titres to Infectious bursal disease virus; MDV, antibody titres to Mareks’ disease virus; SG, antibody titres to *Salmonella enterica* serovar Gallinarum; BCS, body condition score. SNPs in bold were also identified as significant in a previous within-ecotype analysis (Psifidi et al, 2016). | | | |
