# Supplementary material for: Integrating Genetic and Genomic Analyses of Combined Health Data Across Ecotypes to Improve Disease Resistance in Indigenous African Chickens
Source: Front Genet. 2020 Oct 9;11:543890. doi: 10.3389/fgene.2020.543890 (PMC7581896; doi:10.3389/fgene.2020.543890)
Supplement: Supplementary file 4 [file Table_2.docx]

| **Supplementary Table S2: Genes annotated in Galgal6 assembly located within the 200kb candidate regions around each SNP marker identified by GWAS. Genes in bold were also located within candidate regions associated with these traits in a previous within-ecotype analyses (Psifidi et al, 2016).** | | | |
| --- | --- | --- | --- |
| **Trait** | **Gene ID** | **Gene name** | **Gene description** |
| BCS | ENSGALG00000032768 | SOX5 | SRY-box 5 |
|  | ENSGALG00000049237 | gga-mir-6608-1 | gga-mir-6608-2 |
|  | ENSGALG00000049909 | gga-mir-6608-1 | gga-mir-6608-2 |
|  | ENSGALG00000049744 | gga-mir-6608-1 | gga-mir-6608-2 |
|  | ENSGALG00000016027 | CBR3 | carbonyl reductase 3 |
|  | ENSGALG00000016036 | DOP1B | DOP1 leucine zipper like protein B |
|  | ENSGALG00000035329 | gga-mir-7452 | gga-mir-7452 |
|  | ENSGALG00000036556 | MORC4 | MORC family CW-type zinc finger 4 |
|  | ENSGALG00000016042 | CHAF1B | chromatin assembly factor 1 subunit B |
|  | ENSGALG00000049895 |  |  |
|  | ENSGALG00000054431 |  |  |
|  | ENSGALG00000039553 | SULF1 | sulfatase 1 |
|  | ENSGALG00000055117 |  |  |
|  | ENSGALG00000031175 | LZTR1 | leucine zipper like transcription regulator 1 |
|  | ENSGALG00000037370 | NAV1 | neuron navigator 1 |
|  | ENSGALG00000026033 | SYT2 | synaptotagmin 2 |
|  | ENSGALG00000000318 | CSRP1 | cysteine and glycine rich protein 1 |
|  | ENSGALG00000019802 | PHLDA3 | pleckstrin homology like domain family A member 3 |
|  | ENSGALG00000000313 | TNNI1 | troponin I type 1 (skeletal, slow) |
|  | ENSGALG00000000309 |  | ladinin 1 |
|  | ENSGALG00000000302 | TNNT2 | troponin T2, cardiac type |
|  | ENSGALG00000007777 | SLC39A10 | solute carrier family 39 member 10 |
|  | ENSGALG00000037678 |  | baculoviral IAP repeat-containing protein 5.1-like |
|  | ENSGALG00000038543 | KCNH7 | potassium voltage-gated channel subfamily H member 7 |
|  | ENSGALG00000041192 | IFIH1 | interferon induced with helicase C domain 1 |
|  | ENSGALG00000011099 | FAP | fibroblast activation protein alpha |
|  | ENSGALG00000005367 | MRPS22 | mitochondrial ribosomal protein S22 |
|  | ENSGALG00000050790 |  |  |
|  | ENSGALG00000053777 |  |  |
|  | ENSGALG00000047491 |  |  |
|  | ENSGALG00000037387 | CLSTN2 | calsyntenin 2 |
| BW | ENSGALG00000007140 | NRP1 | neuropilin 1 |
|  | ENSGALG00000006906 | CHM | CHM, Rab escort protein 1 |
|  | ENSGALG00000028553 | gga-mir-6704 | gga-mir-6704 |
|  | ENSGALG00000006919 | POF1B | premature ovarian failure, 1B |
|  | ENSGALG00000047808 |  |  |
|  | **ENSGALG00000015967** | **LRRTM1** | **leucine rich repeat transmembrane neuronal 1** |
|  | ENSGALG00000049617 |  |  |
|  | ENSGALG00000040729 |  |  |
|  | ENSGALG00000054561 |  |  |
|  | ENSGALG00000039335 | SLC25A22 | solute carrier family 25 member 22 |
|  | ENSGALG00000025149 | gga-mir-1663 | gga-mir-1663 |
|  | **ENSGALG00000014297** | **IRF7** | **interferon regulatory factor 7** |
|  | ENSGALG00000008890 | ADGRL4 | adhesion G protein-coupled receptor L4 |
|  | ENSGALG00000025158 | gga-mir-1620 | gga-mir-1620 |
|  | ENSGALG00000039354 | VTG1 | vitellogenin 1 |
| Cestodes | ENSGALG00000054077 |  |  |
|  | ENSGALG00000035201 |  | heat shock protein family A (Hsp70) member 14 |
|  | **ENSGALG00000013920** | **SUV39H2** | **suppressor of variegation 3-9 homolog 2** |
|  | **ENSGALG00000000451** | **MEIG1** | **meiosis/spermiogenesis associated 1** |
|  | **ENSGALG00000006633** | **TMEM243** | **transmembrane protein 243** |
|  | ENSGALG00000017274 | CD9 | CD9 molecule |
|  | ENSGALG00000014324 | SLC2A14 | solute carrier family 2 member 14 |
|  | ENSGALG00000053547 |  |  |
|  | ENSGALG00000040184 | RIMKLB | ribosomal modification protein rimK like family member B |
|  | ENSGALG00000037015 | DACH1 | dachshund family transcription factor 1 |
|  | ENSGALG00000016907 | KLHL1 | kelch like family member 1 |
|  | ENSGALG00000017242 | FZD4 | frizzled class receptor 4 |
|  | ENSGALG00000051921 |  |  |
|  | ENSGALG00000017246 | ME3 | malic enzyme 3 |
|  | ENSGALG00000054086 |  |  |
|  | ENSGALG00000044187 |  | lipase maturation factor 1 |
|  | ENSGALG00000025535 | gga-mir-1554 | gga-mir-1554 |
|  | **ENSGALG00000005699** | **NLK** | **nemo like kinase** |
|  | **ENSGALG00000005702** | **TMEM97** | **transmembrane protein 97** |
|  | **ENSGALG00000005737** | **TNFAIP1** | **TNF alpha induced protein 1** |
|  | ENSGALG00000039169 | MLH1 | mutL homolog 1 |
|  | ENSGALG00000046957 |  |  |
|  | ENSGALG00000049164 |  |  |
|  | ENSGALG00000031997 | SFRP4 | secreted frizzled related protein 4 |
|  | ENSGALG00000053440 |  |  |
|  | ENSGALG00000053958 |  |  |
|  | ENSGALG00000000667 | EDN2 | endothelin 2 |
|  | ENSGALG00000024933 | 5S_rRNA | 5S ribosomal RNA |
|  | ENSGALG00000035241 |  |  |
|  | ENSGALG00000032770 | VRK2 | vaccinia related kinase 2 |
|  | ENSGALG00000047366 |  |  |
|  | ENSGALG00000049908 |  |  |
|  | ENSGALG00000047629 |  |  |
|  | ENSGALG00000053828 |  |  |
|  | **ENSGALG00000011242** | **OBSL1** | **obscurin like 1** |
|  | ENSGALG00000032915 | CHPF | chondroitin polymerizing factor |
|  | **ENSGALG00000011252** |  | **rac GTPase-activating protein 1-like** |
|  | **ENSGALG00000011318** | **DNPEP** | **aspartyl aminopeptidase** |
|  | ENSGALG00000033811 | PTPRN | protein tyrosine phosphatase, receptor type N |
|  | ENSGALG00000034139 |  |  |
|  | **ENSGALG00000011324** |  | **tubulin alpha 4b** |
|  | **ENSGALG00000000433** |  | **tubulin alpha 4a** |
|  | **ENSGALG00000011328** | **GLB1L** | **galactosidase beta 1 like** |
|  | **ENSGALG00000011649** | **ATG9A** | **autophagy related 9A** |
|  | ENSGALG00000029672 | ABCB6 | ATP binding cassette subfamily B member 6 (Langereis blood group) |
|  | **ENSGALG00000011343** | **CNPPD1** | **cyclin Pas1/PHO80 domain containing 1** |
|  | ENSGALG00000052271 | SLC23A3 | solute carrier family 23 member 3 |
|  | ENSGALG00000011335 | NHEJ1 | non-homologous end joining factor 1 |
|  | ENSGALG00000048969 |  |  |
|  | ENSGALG00000048432 |  |  |
| *Eimeria* | ENSGALG00000048193 |  |  |
|  | **ENSGALG00000003011** | **TOM1L1** | **target of myb1 like 1 membrane trafficking protein** |
|  | **ENSGALG00000003033** | **STXBP4** | **syntaxin binding protein 4** |
|  | ENSGALG00000025478 | gga-mir-1765 | gga-mir-1765 |
| IBDV | ENSGALG00000014186 | MPPED1 | metallophosphoesterase domain containing 1 |
|  | ENSGALG00000025782 | gga-mir-6616 | gga-mir-6616 |
|  | ENSGALG00000037655 | BCL9 | B-cell CLL/lymphoma 9 |
|  | ENSGALG00000015488 | GJA8 | gap junction protein alpha 8 |
|  | ENSGALG00000015491 | GPR89A | G protein-coupled receptor 89A |
|  | ENSGALG00000051948 |  |  |
|  | ENSGALG00000017191 | ANGPTL5 | angiopoietin like 5 |
|  | ENSGALG00000052779 |  | G2/M phase-specific E3 ubiquitin-protein ligase-like |
|  | ENSGALG00000050036 |  |  |
|  | ENSGALG00000003916 | SIAH1 | siah E3 ubiquitin protein ligase 1 |
|  | ENSGALG00000035675 |  | coiled-coil domain containing 57 |
|  | ENSGALG00000040896 | FASN | fatty acid synthase |
|  | ENSGALG00000034115 | DUS1L | dihydrouridine synthase 1 like |
|  | ENSGALG00000002841 | RFNG | RFNG O-fucosylpeptide 3-beta-N-acetylglucosaminyltransferase |
|  | ENSGALG00000002849 | DCXR | dicarbonyl and L-xylulose reductase |
|  | ENSGALG00000043607 | CENPX | centromere protein X |
|  | ENSGALG00000002900 | NOTUM | NOTUM, palmitoleoyl-protein carboxylesterase |
|  | ENSGALG00000029882 | MYADML2 | myeloid associated differentiation marker like 2 |
|  | ENSGALG00000002932 | NME2 | NME/NM23 nucleoside diphosphate kinase 2 |
|  | ENSGALG00000055093 |  |  |
|  | ENSGALG00000047846 |  |  |
|  | ENSGALG00000027365 | HIVEP3 | human immunodeficiency virus type I enhancer binding protein 3 |
|  | ENSGALG00000000678 | CITED4 | Cbp/p300 interacting transactivator with Glu/Asp rich carboxy-terminal domain 4 |
|  | ENSGALG00000000720 |  | guanylate binding protein 1 |
|  | **ENSGALG00000000723** | **STX12** | **syntaxin 12** |
|  | **ENSGALG00000000729** | **PPP1R8** | **protein phosphatase 1 regulatory subunit 8** |
|  | ENSGALG00000025671 | SCARNA1 | small Cajal body-specific RNA 1 |
|  | **ENSGALG00000000731** | **THEMIS2** | **thymocyte selection associated family member 2** |
|  | **ENSGALG00000000737** | **SMPDL3B** | **sphingomyelin phosphodiesterase acid like 3B** |
|  | ENSGALG00000030305 | XKR8 | XK related 8 |
|  | ENSGALG00000038672 |  | ATPase inhibitory factor 1 |
|  | **ENSGALG00000000814** | **HNRNPR** | **heterogeneous nuclear ribonucleoprotein R** |
|  | ENSGALG00000013409 |  |  |
|  | ENSGALG00000047941 |  |  |
|  | ENSGALG00000040986 | FAM76A | family with sequence similarity 76 member A |
|  | ENSGALG00000037603 | SESN2 | sestrin-2-like |
|  | ENSGALG00000055135 |  |  |
|  | ENSGALG00000042485 |  |  |
|  | ENSGALG00000054143 |  |  |
|  | ENSGALG00000021931 | SNORA73 | Small nucleolar RNA SNORA73 family |
|  | ENSGALG00000030097 | RCC1 | regulator of chromosome condensation 1 |
|  | ENSGALG00000038218 |  |  |
|  | ENSGALG00000054395 |  |  |
|  | ENSGALG00000021935 | gga-mir-124b | gga-mir-124b |
|  | ENSGALG00000029066 |  | discoidin, CUB and LCCL domain-containing protein 1-like |
|  | ENSGALG00000004328 | IGHMBP2 | immunoglobulin mu binding protein 2 |
|  | ENSGALG00000004280 |  |  |
|  | ENSGALG00000004267 | SIGIRR | single Ig and TIR domain containing |
|  | ENSGALG00000026970 |  | interferon-induced transmembrane protein 1-like |
|  | ENSGALG00000004243 |  | interferon-induced transmembrane protein 3-like |
|  | ENSGALG00000045199 |  | dispanin subfamily A member 2b-like |
|  | ENSGALG00000004239 | IFITM5 | interferon induced transmembrane protein 5 |
|  | ENSGALG00000053957 |  |  |
|  | ENSGALG00000052099 |  |  |
|  | ENSGALG00000006697 | TOLLIP | toll interacting protein |
|  | ENSGALG00000050455 | gga-mir-210a | gga-mir-210a |
|  | ENSGALG00000033498 |  |  |
|  | ENSGALG00000029260 | H-RAS | HRas proto-oncogene, GTPase |
|  | ENSGALG00000010703 | DGLUCY | chromosome 5 C14orf159 homolog |
|  | ENSGALG00000042374 |  | phosphodiesterase 11A |
|  | ENSGALG00000009224 | TTC30B | tetratricopeptide repeat domain 30B |
|  | ENSGALG00000009240 | NFE2L2 | nuclear factor, erythroid 2 like 2 |
|  | ENSGALG00000012444 | ACVR2A | activin A receptor type 2A |
|  | ENSGALG00000001951 |  | family with sequence similarity 102 member B |
|  | ENSGALG00000001972 |  | pre-mRNA processing factor 38B |
|  | ENSGALG00000002001 | STXBP3 | syntaxin binding protein 3 |
|  | ENSGALG00000002090 | GPSM2 | G-protein signaling modulator 2 |
| MDV | ENSGALG00000050622 |  |  |
|  | ENSGALG00000054901 |  |  |
|  | ENSGALG00000000817 | ZNF821 | zinc finger protein 821 |
|  | ENSGALG00000000848 | AP1G1 | adaptor related protein complex 1 gamma 1 subunit |
|  | ENSGALG00000025302 | SNORD71 | small nucleolar RNA, C/D box 71 |
|  | ENSGALG00000000875 | PHLPP2 | PH domain and leucine rich repeat protein phosphatase 2 |
|  | ENSGALG00000000893 | TAT | tyrosine aminotransferase |
|  | ENSGALG00000000901 |  |  |
|  | ENSGALG00000000907 | KARS | lysyl-tRNA synthetase |
|  | ENSGALG00000000908 | ADAT1 | adenosine deaminase, tRNA specific 1 |
|  | **ENSGALG00000008349** | **LMCD1** | **LIM and cysteine rich domains 1** |
|  | **ENSGALG00000008351** | **CAV3** | **caveolin 3** |
|  | ENSGALG00000010762 | OPN3 | opsin 3 |
|  | ENSGALG00000039772 | RGS7 | regulator of G-protein signaling 7 |
| PM | ENSGALG00000002170 |  | bile acid receptor-like |
|  | ENSGALG00000054224 |  | synaptonemal complex protein 1-like |
|  | ENSGALG00000002511 |  | thyroid stimulating hormone beta |
|  | ENSGALG00000002550 | TSHB | thyroid stimulating hormone beta |
|  | ENSGALG00000014589 |  | AN1-type zinc finger protein 5-like |
|  | ENSGALG00000041163 | ABHD17A | abhydrolase domain containing 17A |
|  | ENSGALG00000021777 | gga-mir-211 | gga-mir-211 |
|  | ENSGALG00000001043 | REXO1 | RNA exonuclease 1 homolog |
|  | ENSGALG00000025382 | gga-mir-1647 | gga-mir-1647 |
|  | ENSGALG00000001084 | ATP8B3 | ATPase phospholipid transporting 8B3 |
|  | ENSGALG00000001093 | TCF3 | transcription factor 3 |
| SG | **ENSGALG00000011013** | **PCNX2** | **pecanex homolog 2 (Drosophila)** |
