# Supplementary material for: Integrating Genetic and Genomic Analyses of Combined Health Data Across Ecotypes to Improve Disease Resistance in Indigenous African Chickens
Source: Front Genet. 2020 Oct 9;11:543890. doi: 10.3389/fgene.2020.543890 (PMC7581896; doi:10.3389/fgene.2020.543890)
Supplement: Supplementary file 5 [file Table_3.docx]

| **Supplementary Table S3: High and moderate predicted variants (identified from 200kb windows around significant and suggestive SNPs identified by GWAS for disease and production traits** | | | | | | |
| --- | --- | --- | --- | --- | --- | --- |
| **Trait** | **Population** | **Position (Chr:Start)** | **Consequence** | **Impact** | **Gene** | **Gene ID** |
| IBDV | Both | 23:2299219 | splice_acceptor_variant | HIGH | PHACTR4 | ENSGALG00000042485 |
|  | Both | 8:1605190 | start_lost | HIGH | ASPSCR1 |  |
|  | Both | 18:5043153 | stop_gained | HIGH | LONP2 |  |
|  | Both | 11:7379361 | stop_gained | HIGH | PDZK1 |  |
|  | Both | 1:93917610 | splice_donor_variant | HIGH | SLC25A24 |  |
|  | Both | 23:1516429 | missense_variant | MODERATE | EFCAB6 | ENSGALG00000052391 |
|  | Both | 23:1516429 | missense_variant | MODERATE | EFCAB6 | ENSGALG00000052391 |
|  | Both | 23:1516518 | missense_variant | MODERATE | EFCAB6 | ENSGALG00000052391 |
|  | Both | 23:1516518 | missense_variant | MODERATE | EFCAB6 | ENSGALG00000052391 |
|  | Both | 23:1518077 | missense_variant | MODERATE | muc5f | ENSGALG00000044418 |
|  | Both | 23:1518077 | missense_variant | MODERATE | Muc5f | ENSGALG00000034144 |
|  | Both | 23:1519073 | missense_variant | MODERATE |  | ENSGALG00000006873 |
|  | Both | 23:1519073 | missense_variant | MODERATE | Novel gene; orthologue of PHRF1 | ENSGALG00000006873 |
|  | Both | 23:1519876 | missense_variant | MODERATE | Novel gene; orthologue of PHRF1 | ENSGALG00000006873 |
|  | Both | 23:1519876 | missense_variant | MODERATE | Novel gene; orthologue of PHRF1 | ENSGALG00000006873 |
|  | Both | 23:1520154 | missense_variant | MODERATE | Novel gene; orthologue of PHRF1 | ENSGALG00000006873 |
|  | Both | 23:1520154 | missense_variant | MODERATE | Novel gene; orthologue of PHRF1 | ENSGALG00000006873 |
|  | Both | 23:1520253 | missense_variant | MODERATE | Novel gene; orthologue of PHRF1 | ENSGALG00000050582 |
|  | Both | 23:1520253 | missense_variant | MODERATE | Novel gene; SITS-binding protein-like | ENSGALG00000004393 |
|  | Both | 23:1520446 | missense_variant | MODERATE | Novel gene; SITS-binding protein-like | ENSGALG00000004393 |
|  | Both | 23:1520509 | missense_variant | MODERATE | Novel gene; SITS-binding protein-like | ENSGALG00000004393 |
|  | Both | 23:1520528 | missense_variant | MODERATE | Novel gene; phospholipase A2 group IVE-like 5 | ENSGALG00000044307 |
|  | Both | 23:1520536 | missense_variant | MODERATE | Novel gene; phospholipase A2 group IVE-like 5 | ENSGALG00000044307 |
|  | Both | 23:1520541 | missense_variant | MODERATE | Novel gene; orthologue of PLA2G4E | ENSGALG00000028402 |
|  | Jarso | 23:1520542 | missense_variant | MODERATE | Novel gene; phospholipase A2 group IVE-like 2 | ENSGALG00000026584 |
|  | Jarso | 23:1520602 | missense_variant | MODERATE | Novel gene; phospholipase A2 group IVE-like 2 | ENSGALG00000026584 |
|  | Jarso | 23:1520619 | missense_variant | MODERATE | Novel gene; family with sequence similarity 102 member B | ENSGALG00000001951 |
|  | Jarso | 23:1520622 | missense_variant | MODERATE | Novel gene; family with sequence similarity 102 member B | ENSGALG00000001951 |
|  | Both | 23:1520623 | missense_variant | MODERATE | Novel gene; family with sequence similarity 102 member B | ENSGALG00000001951 |
|  | Both | 23:1519822 | missense_variant | MODERATE | Novel gene; family with sequence similarity 102 member B | ENSGALG00000001951 |
|  | Both | 23:1519822 | missense_variant | MODERATE | Novel gene; family with sequence similarity 102 member B | ENSGALG00000001951 |
|  | Both | 23:1520093 | missense_variant | MODERATE | Novel gene; fibronectin type III domain containing 7 | ENSGALG00000001983 |
|  | Jarso | 23:1520093 | missense_variant | MODERATE | Novel gene; fibronectin type III domain containing 7 | ENSGALG00000001983 |
|  | Both | 23:1566285 | missense_variant | MODERATE | Novel gene; orthologue of FNDC7 | ENSGALG00000052265 |
|  | Both | 23:1566603 | missense_variant | MODERATE | Novel gene; orthologue of AKNAD1 | ENSGALG00000051777 |
|  | Both | 23:1566966 | missense_variant | MODERATE | Novel gene; orthologue of AKNAD1 | ENSGALG00000051777 |
|  | Jarso | 23:1567164 | missense_variant | MODERATE | Novel gene; orthologue of AKNAD1 | ENSGALG00000051777 |
|  | Jarso | 23:1566520 | missense_variant | MODERATE | Novel gene; orthologue of AKNAD1 | ENSGALG00000051777 |
|  | Jarso | 23:1575967 | missense_variant | MODERATE | Novel gene; orthologue of AKNAD1 | ENSGALG00000051777 |
|  | Jarso | 23:1578460 | missense_variant | MODERATE | Novel gene; orthologue of AKNAD1 | ENSGALG00000051777 |
|  | Jarso | 23:1578750 | missense_variant | MODERATE | Novel gene; orthologue of AKNAD1 | ENSGALG00000051777 |
|  | Jarso | 23:1578678 | missense_variant | MODERATE | Novel gene; orthologue of AKNAD1 | ENSGALG00000051777 |
|  | Jarso | 23:1594171 | missense_variant | MODERATE | Novel gene; orthologue of AKNAD1 | ENSGALG00000051777 |
|  | Jarso | 23:1594171 | missense_variant | MODERATE | Novel gene; orthologue of AKNAD1 | ENSGALG00000051777 |
|  | Horro | 23:1594171 | missense_variant | MODERATE | Novel gene; orthologue of AKNAD1 | ENSGALG00000051777 |
|  | Horro | 23:1631578 | missense_variant | MODERATE | Novel gene; orthologue of AKNAD1 | ENSGALG00000051777 |
|  | Both | 23:1631578 | missense_variant | MODERATE | Novel gene; orthologue of AKNAD1 | ENSGALG00000051777 |
|  | Both | 23:1648018 | missense_variant | MODERATE | Novel gene; orthologue of AKNAD1 | ENSGALG00000051777 |
|  | Both | 23:1648018 | missense_variant | MODERATE | Novel gene; orthologue of AKNAD1 | ENSGALG00000051777 |
|  | Both | 8:1605241 | missense_variant | MODERATE | Novel gene; orthologue of AKNAD1 | ENSGALG00000051777 |
|  | Both | 8:1605680 | missense_variant | MODERATE | Novel gene; orthologue of AKNAD1 | ENSGALG00000051777 |
|  | Horro | 8:1608139 | missense_variant | MODERATE | Novel gene; orthologue of AKNAD1 | ENSGALG00000051777 |
|  | Horro | 8:1618561 | missense_variant | MODERATE | Novel gene; coiled-coil domain containing 57 | ENSGALG00000035675 |
|  | Horro | 8:1618561 | missense_variant | MODERATE | Novel gene; coiled-coil domain containing 57 | ENSGALG00000035675 |
|  | Jarso | 8:1624574 | missense_variant | MODERATE | Novel gene; coiled-coil domain containing 57 | ENSGALG00000035675 |
|  | Jarso | 8:1624574 | missense_variant | MODERATE | Novel gene; coiled-coil domain containing 57 | ENSGALG00000035675 |
|  | Jarso | 8:1608197 | missense_variant | MODERATE | Novel gene; coiled-coil domain containing 57 | ENSGALG00000035675 |
|  | Both | 8:1608212 | missense_variant | MODERATE | Novel gene; coiled-coil domain containing 57 | ENSGALG00000035675 |
|  | Both | 8:1608223 | missense_variant | MODERATE | Novel gene; coiled-coil domain containing 57 | ENSGALG00000035675 |
|  | Both | 8:1608864 | missense_variant | MODERATE | Novel gene; coiled-coil domain containing 57 | ENSGALG00000035675 |
|  | Both | 8:1639448 | missense_variant | MODERATE | Novel gene; coiled-coil domain containing 57 | ENSGALG00000035675 |
|  | Both | 8:1639448 | missense_variant | MODERATE | Novel gene; coiled-coil domain containing 57 | ENSGALG00000035675 |
|  | Both | 8:1654687 | missense_variant | MODERATE | Novel gene; coiled-coil domain containing 57 | ENSGALG00000035675 |
|  | Both | 8:1654687 | missense_variant | MODERATE | Novel gene; coiled-coil domain containing 57 | ENSGALG00000035675 |
|  | Both | 8:1686634 | missense_variant | MODERATE | Novel gene; coiled-coil domain containing 57 | ENSGALG00000035675 |
|  | Both | 8:1686634 | missense_variant,  splice_region_variant | MODERATE | Novel gene; coiled-coil domain containing 57 | ENSGALG00000035675 |
|  | Both | 8:1686634 | missense_variant | MODERATE | Novel gene; guanylate binding protein 1 | ENSGALG00000000720 |
|  | Both | 8:1686634 | missense_variant | MODERATE | Novel gene; guanylate binding protein 1 | ENSGALG00000000720 |
|  | Both | 8:1722133 | missense_variant | MODERATE | Novel gene; guanylate binding protein 1 | ENSGALG00000000720 |
|  | Both | 8:1729715 | missense_variant | MODERATE | Novel gene; guanylate binding protein 1 | ENSGALG00000000720 |
|  | Horro | 8:1729715 | missense_variant | MODERATE | Novel gene; guanylate binding protein 1 | ENSGALG00000000720 |
|  | Horro | 8:1729715 | missense_variant | MODERATE | Novel gene; guanylate binding protein 1 | ENSGALG00000000720 |
|  | Horro | 8:1730596 | missense_variant | MODERATE | Novel gene; guanylate binding protein 1 | ENSGALG00000000720 |
|  | Both | 8:1730596 | missense_variant | MODERATE | Novel gene; guanylate binding protein 1 | ENSGALG00000000720 |
|  | Both | 8:1730596 | missense_variant | MODERATE | Novel gene; guanylate binding protein 1 | ENSGALG00000000720 |
|  | Horro | 8:1729846 | missense_variant | MODERATE | Novel gene; guanylate binding protein 1 | ENSGALG00000000720 |
|  | Horro | 8:1729846 | missense_variant | MODERATE | Novel gene; guanylate binding protein 1 | ENSGALG00000000720 |
|  | Both | 8:1729846 | missense_variant | MODERATE | Novel gene; guanylate binding protein 1 | ENSGALG00000000720 |
|  | Both | 8:1740875 | missense_variant | MODERATE | Novel gene; guanylate binding protein 1 | ENSGALG00000000720 |
|  | Both | 8:1740875 | missense_variant | MODERATE | Novel gene; guanylate binding protein 1 | ENSGALG00000000720 |
|  | Both | 8:1751901 | missense_variant | MODERATE | Novel gene; guanylate binding protein 1 | ENSGALG00000000720 |
|  | Horro | 8:1751901 | missense_variant | MODERATE | Novel gene; guanylate binding protein 1 | ENSGALG00000000720 |
|  | Jarso | 18:4985636 | missense_variant | MODERATE | Novel gene; guanylate binding protein 1 | ENSGALG00000000720 |
|  | Jarso | 18:4995376 | missense_variant | MODERATE | Novel gene; guanylate binding protein 1 | ENSGALG00000000720 |
|  | Jarso | 18:4995963 | missense_variant | MODERATE | Novel gene; guanylate binding protein 1 | ENSGALG00000000720 |
|  | Jarso | 18:4999012 | missense_variant | MODERATE | Novel gene; guanylate binding protein 1 | ENSGALG00000000720 |
|  | Jarso | 18:5002338 | missense_variant | MODERATE | Novel gene; guanylate binding protein 1 | ENSGALG00000000720 |
|  | Both | 18:4998693 | missense_variant | MODERATE | Novel gene; guanylate binding protein 1 | ENSGALG00000000720 |
|  | Both | 18:4999675 | missense_variant | MODERATE | Novel gene; guanylate binding protein 1 | ENSGALG00000000720 |
|  | Both | 18:5000466 | missense_variant | MODERATE | Novel gene; guanylate binding protein 1 | ENSGALG00000000720 |
|  | Both | 18:5002460 | missense_variant | MODERATE | Novel gene; orthologue of CTSL/CTSV/CTSF | ENSGALG00000013409 |
|  | Both | 18:5052672 | missense_variant | MODERATE | Novel gene; orthologue of CTSL/CTSV/CTSF | ENSGALG00000013409 |
|  | Both | 11:7379314 | missense_variant | MODERATE | Novel gene; orthologue of CTSL/CTSV/CTSF | ENSGALG00000013409 |
|  | Both | 11:7379364 | missense_variant | MODERATE | Novel gene; orthologue of CTSL/CTSV/CTSF | ENSGALG00000013409 |
|  | Both | 11:7379366 | missense_variant | MODERATE | Novel gene; orthologue of CTSL/CTSV/CTSF | ENSGALG00000047941 |
|  | Both | 11:7379938 | missense_variant | MODERATE | Novel gene; orthologue of CTSL/CTSV/CTSF | ENSGALG00000047941 |
|  | Horro | 5:1781053 | missense_variant | MODERATE | Novel gene; orthologue of CTSL/CTSV/CTSF | ENSGALG00000047941 |
|  | Horro | 5:1781241 | missense_variant | MODERATE | Novel gene; orthologue of CTSL/CTSV/CTSF | ENSGALG00000047941 |
|  | Both | 5:1781261 | missense_variant | MODERATE | Novel gene; orthologue of CTSL/CTSV/CTSF | ENSGALG00000013409 |
|  | Both | 5:1781319 | missense_variant | MODERATE | Novel gene; orthologue of CTSL/CTSV/CTSF | ENSGALG00000013409 |
|  | Both | 5:1712627 | missense_variant | MODERATE | Novel gene; orthologue of PHACTR4 | ENSGALG00000042485 |
|  | Both | 5:1713313 | missense_variant | MODERATE | Novel gene; orthologue of PHACTR4 | ENSGALG00000042485 |
|  | Both | 5:1713442 | missense_variant | MODERATE | Novel gene; orthologue of PHACTR4 | ENSGALG00000042485 |
|  | Both | 5:1698850 | missense_variant | MODERATE | Novel gene; peptide methionine sulfoxide reductase MsrA-like | ENSGALG00000041258 |
|  | Both | 5:1703896 | missense_variant | MODERATE | ACP6 | ENSGALG00000015485 |
|  | Both | 5:1706312 | missense_variant | MODERATE | ACP6 | ENSGALG00000015485 |
|  | Jarso | 5:1706313 | missense_variant | MODERATE | ACP6 | ENSGALG00000015485 |
|  | Jarso | 5:1706529 | missense_variant | MODERATE | ACP6 | ENSGALG00000015485 |
|  | Jarso | 5:1706605 | missense_variant | MODERATE | AGPS | ENSGALG00000009236 |
|  | Jarso | 5:1706745 | missense_variant | MODERATE | ASAP3 | ENSGALG00000036949 |
|  | Jarso | 5:1707144 | missense_variant | MODERATE | ASAP3 | ENSGALG00000036949 |
|  | Jarso | 5:1674945 | missense_variant | MODERATE | ASAP3 | ENSGALG00000036949 |
|  | Jarso | 5:1684754 | missense_variant | MODERATE | ASAP3 | ENSGALG00000036949 |
|  | Both | 5:1684829 | missense_variant | MODERATE | ASPSCR1 | ENSGALG00000039172 |
|  | Both | 5:1684849 | missense_variant | MODERATE | ASPSCR1 | ENSGALG00000039172 |
|  | Both | 5:1702474 | missense_variant | MODERATE | ASPSCR1 | ENSGALG00000039172 |
|  | Both | 5:1707230 | missense_variant | MODERATE | ASPSCR1 | ENSGALG00000039172 |
|  | Jarso | 5:1707269 | missense_variant | MODERATE | C1H11ORF70 | ENSGALG00000017189 |
|  | Both | 5:1667397 | missense_variant | MODERATE | CEP126 | ENSGALG00000017190 |
|  | Jarso | 5:1631877 | missense_variant | MODERATE | CEP126 | ENSGALG00000017190 |
|  | Both | 5:1633135 | missense_variant | MODERATE | CEP126 | ENSGALG00000017190 |
|  | Both | 5:1635767 | missense_variant | MODERATE | CEP126 | ENSGALG00000017190 |
|  | Both | 5:1636032 | missense_variant | MODERATE | CEP126 | ENSGALG00000017190 |
|  | Both | 5:14873367 | missense_variant | MODERATE | CEP126 | ENSGALG00000017190 |
|  | Both | 5:14882941 | missense_variant | MODERATE | CEP126 | ENSGALG00000017190 |
|  | Both | 5:16071938 | missense_variant | MODERATE | CEP126 | ENSGALG00000017190 |
|  | Jarso | 5:16072645 | missense_variant | MODERATE | CEP126 | ENSGALG00000017190 |
|  | Jarso | 5:16072835 | missense_variant | MODERATE | CEP126 | ENSGALG00000017190 |
|  | Both | 5:16073711 | missense_variant | MODERATE | CEP126 | ENSGALG00000017190 |
|  | Both | 5:16073852 | missense_variant | MODERATE | CEP126 | ENSGALG00000017190 |
|  | Both | 5:16074213 | missense_variant | MODERATE | CEP126 | ENSGALG00000017190 |
|  | Both | 5:16079301 | missense_variant | MODERATE | CEP126 | ENSGALG00000017190 |
|  | Both | 5:16079316 | missense_variant | MODERATE | CEP126 | ENSGALG00000017190 |
|  | Both | 7:15680133 | missense_variant | MODERATE | CEP126 | ENSGALG00000017190 |
|  | Both | 7:15680334 | missense_variant | MODERATE | CEP126 | ENSGALG00000017190 |
|  | Both | 7:15749910 | missense_variant | MODERATE | CEP126 | ENSGALG00000017190 |
|  | Both | 5:44209234 | missense_variant | MODERATE | CEP126 | ENSGALG00000017190 |
|  | Jarso | 5:44209234 | missense_variant | MODERATE | CEP126 | ENSGALG00000017190 |
|  | Jarso | 5:44209271 | missense_variant | MODERATE | CEP126 | ENSGALG00000017190 |
|  | Jarso | 5:44209271 | missense_variant | MODERATE | CEP126 | ENSGALG00000017190 |
|  | Jarso | 5:44209283 | missense_variant | MODERATE | CEP126 | ENSGALG00000017190 |
|  | Jarso | 5:44209283 | missense_variant | MODERATE | CEP126 | ENSGALG00000017190 |
|  | Jarso | 5:44209336 | missense_variant | MODERATE | CEP126 | ENSGALG00000017190 |
|  | Both | 5:44209336 | missense_variant | MODERATE | CEP126 | ENSGALG00000017190 |
|  | Both | 5:44209336 | missense_variant,  splice_region_variant | MODERATE | CEP126 | ENSGALG00000017190 |
|  | Both | 5:44274217 | missense_variant | MODERATE | CLCC1 | ENSGALG00000002106 |
|  | Both | 5:44274217 | missense_variant | MODERATE | CLCC1 | ENSGALG00000002106 |
|  | Both | 5:44274217 | missense_variant | MODERATE | CLCC1 | ENSGALG00000002106 |
|  | Both | 23:2221363 | missense_variant | MODERATE | CLCC1 | ENSGALG00000002106 |
|  | Horro | 23:2221363 | missense_variant | MODERATE | CLCC1 | ENSGALG00000002106 |
|  | Horro | 23:2222625 | missense_variant | MODERATE | CLCC1 | ENSGALG00000002106 |
|  | Horro | 23:2222625 | missense_variant | MODERATE | DCXR | ENSGALG00000002849 |
|  | Horro | 23:2228709 | missense_variant | MODERATE | DUS1L | ENSGALG00000034115 |
|  | Jarso | 23:2228808 | missense_variant | MODERATE | DUS1L | ENSGALG00000034115 |
|  | Jarso | 23:2222413 | missense_variant | MODERATE | FAM76A | ENSGALG00000040986 |
|  | Jarso | 23:2222413 | missense_variant | MODERATE | FAM76A | ENSGALG00000040986 |
|  | Both | 23:2222745 | missense_variant | MODERATE | FAM76A | ENSGALG00000040986 |
|  | Both | 23:2222745 | missense_variant | MODERATE | FAM76A | ENSGALG00000040986 |
|  | Both | 23:2228854 | missense_variant | MODERATE | FAM76A | ENSGALG00000040986 |
|  | Both | 1:68896887 | missense_variant | MODERATE | FAM76A | ENSGALG00000040986 |
|  | Jarso | 1:68896942 | missense_variant | MODERATE | FAM76A | ENSGALG00000040986 |
|  | Both | 1:93811602 | missense_variant | MODERATE | FAM76A | ENSGALG00000040986 |
|  | Both | 1:93811602 | missense_variant | MODERATE | FAM76A | ENSGALG00000040986 |
|  | Both | 1:93817233 | missense_variant | MODERATE | FASN | ENSGALG00000040896 |
|  | Both | 1:93817381 | missense_variant | MODERATE | FASN | ENSGALG00000040896 |
|  | Both | 1:93817355 | missense_variant | MODERATE | FASN | ENSGALG00000040896 |
|  | Both | 1:93890322 | missense_variant | MODERATE | FASN | ENSGALG00000040896 |
|  | Both | 1:93917563 | missense_variant | MODERATE | FASN | ENSGALG00000040896 |
|  | Both | 1:93917571 | missense_variant | MODERATE | FASN | ENSGALG00000040896 |
|  | Both | 1:93917615 | missense_variant | MODERATE | FASN | ENSGALG00000040896 |
|  | Both | 1:93922403 | missense_variant | MODERATE | FASN | ENSGALG00000040896 |
|  | Both | 1:93922403 | missense_variant | MODERATE | FASN | ENSGALG00000040896 |
|  | Both | 1:93922426 | missense_variant | MODERATE | FASN | ENSGALG00000040896 |
|  | Both | 1:93922426 | missense_variant | MODERATE | FASN | ENSGALG00000040896 |
|  | Both | 1:93922459 | missense_variant | MODERATE | FASN | ENSGALG00000040896 |
|  | Both | 1:93922459 | missense_variant | MODERATE | FASN | ENSGALG00000040896 |
|  | Both | 1:93922478 | missense_variant | MODERATE | FASN | ENSGALG00000040896 |
|  | Both | 1:93922478 | missense_variant | MODERATE | GJA5 | ENSGALG00000039452 |
|  | Both | 1:93922715 | missense_variant | MODERATE | GJA8 | ENSGALG00000015488 |
|  | Both | 1:93922715 | missense_variant | MODERATE | GPSM2 | ENSGALG00000002090 |
|  | Both | 1:93922715 | missense_variant | MODERATE | ID3 | ENSGALG00000035317 |
|  | Both | 1:93922715 | missense_variant | MODERATE | IGHMBP2 | ENSGALG00000004328 |
|  | Both | 1:93922762 | missense_variant | MODERATE | IGHMBP2 | ENSGALG00000004328 |
|  | Both | 1:93922762 | missense_variant | MODERATE | IGHMBP2 | ENSGALG00000004328 |
|  | Both | 1:93922762 | missense_variant | MODERATE | IGHMBP2 | ENSGALG00000004328 |
|  | Both | 1:93922762 | missense_variant | MODERATE | IGHMBP2 | ENSGALG00000004328 |
|  | Both | 1:93922762 | missense_variant | MODERATE | IGHMBP2 | ENSGALG00000004328 |
|  | Both | 1:93924313 | missense_variant | MODERATE | IGHMBP2 | ENSGALG00000004328 |
|  | Both | 1:93924313 | missense_variant | MODERATE | IGHMBP2 | ENSGALG00000004328 |
|  | Both | 1:93924313 | missense_variant | MODERATE | LONP2 | ENSGALG00000003942 |
|  | Both | 1:93924313 | missense_variant | MODERATE | LONP2 | ENSGALG00000003942 |
|  | Both | 1:93924313 | missense_variant | MODERATE | LONP2 | ENSGALG00000003942 |
|  | Both | 1:93924707 | missense_variant | MODERATE | LONP2 | ENSGALG00000003942 |
|  | Both | 1:93924707 | missense_variant | MODERATE | LRRC45 | ENSGALG00000002877 |
|  | Both | 1:93924707 | missense_variant | MODERATE | LRRC45 | ENSGALG00000002877 |
|  | Both | 1:93924707 | missense_variant | MODERATE | LRRC45 | ENSGALG00000002877 |
|  | Both | 1:93924707 | missense_variant | MODERATE | LRRC56 | ENSGALG00000041955 |
|  | Both | 1:93926162 | missense_variant | MODERATE | LRRC56 | ENSGALG00000041955 |
|  | Both | 1:93926162 | missense_variant | MODERATE | LRRC56 | ENSGALG00000041955 |
|  | Both | 1:93926162 | missense_variant | MODERATE | LRRC56 | ENSGALG00000041955 |
|  | Both | 1:93926162 | missense_variant | MODERATE | LRRC56 | ENSGALG00000041955 |
|  | Both | 1:93926162 | missense_variant | MODERATE | LRRC56 | ENSGALG00000041955 |
|  | Both | 1:93927893 | missense_variant | MODERATE | LRRC56 | ENSGALG00000041955 |
|  | Both | 1:93927893 | missense_variant | MODERATE | LRRC56 | ENSGALG00000041955 |
|  | Both | 1:93927893 | missense_variant | MODERATE | LRRC56 | ENSGALG00000041955 |
|  | Both | 1:93927893 | missense_variant | MODERATE | LRRC56 | ENSGALG00000041955 |
|  | Both | 1:93927893 | missense_variant | MODERATE | LRRC56 | ENSGALG00000041955 |
|  | Both | 1:184067969 | missense_variant,  splice_region_variant | MODERATE | LRRC56 | ENSGALG00000041955 |
|  | Both | 1:184105408 | missense_variant | MODERATE | MRPL21 | ENSGALG00000004362 |
|  | Both | 1:184107766 | missense_variant | MODERATE | MYADML2 | ENSGALG00000029882 |
|  | Both | 1:184108504 | missense_variant | MODERATE | MYADML2 | ENSGALG00000029882 |
|  | Both | 1:184108514 | missense_variant | MODERATE | OPRD1 | ENSGALG00000036782 |
|  | Both | 1:184110083 | missense_variant | MODERATE | PDZK1 | ENSGALG00000015492 |
|  | Both | 1:184114842 | missense_variant | MODERATE | PDZK1 | ENSGALG00000015492 |
|  | Both | 1:184114875 | missense_variant | MODERATE | PDZK1 | ENSGALG00000015492 |
|  | Both | 1:184115065 | missense_variant | MODERATE | PDZK1 | ENSGALG00000015492 |
|  | Both | 1:184115256 | missense_variant | MODERATE | PDZK1 | ENSGALG00000015492 |
|  | Both | 1:184115259 | missense_variant | MODERATE | PDZK1 | ENSGALG00000015492 |
|  | Both | 1:184115269 | missense_variant | MODERATE | PDZK1 | ENSGALG00000015492 |
|  | Both | 1:184115349 | missense_variant | MODERATE | PDZK1 | ENSGALG00000015492 |
|  | Both | 1:184115361 | missense_variant | MODERATE | PDZK1 | ENSGALG00000015492 |
|  | Both | 1:184115368 | missense_variant | MODERATE | PDZK1 | ENSGALG00000015492 |
|  | Both | 1:184115388 | missense_variant | MODERATE | PDZK1 | ENSGALG00000015492 |
|  | Both | 1:184115389 | missense_variant | MODERATE | PDZK1 | ENSGALG00000015492 |
|  | Both | 1:184115540 | missense_variant | MODERATE | PDZK1 | ENSGALG00000015492 |
|  | Both | 1:184115553 | missense_variant | MODERATE | PDZK1 | ENSGALG00000015492 |
|  | Both | 1:184115673 | missense_variant | MODERATE | PDZK1 | ENSGALG00000015492 |
|  | Both | 1:184115770 | missense_variant | MODERATE | PDZK1 | ENSGALG00000015492 |
|  | Both | 1:184116421 | missense_variant | MODERATE | PDZK1 | ENSGALG00000015492 |
|  | Horro | 1:184116519 | missense_variant | MODERATE | PDZK1 | ENSGALG00000015492 |
|  | Horro | 1:184116552 | missense_variant | MODERATE | PDZK1 | ENSGALG00000015492 |
|  | Horro | 1:184116648 | missense_variant | MODERATE | PDZK1 | ENSGALG00000015492 |
|  | Horro | 1:184130700 | missense_variant | MODERATE | PDZK1 | ENSGALG00000015492 |
|  | Horro | 1:184130769 | missense_variant | MODERATE | PDZK1 | ENSGALG00000015492 |
|  | Horro | 1:184115071 | missense_variant | MODERATE | PDZK1 | ENSGALG00000015492 |
|  | Horro | 1:184115358 | missense_variant | MODERATE | PDZK1 | ENSGALG00000015492 |
|  | Jarso | 1:184115739 | missense_variant | MODERATE | PDZK1 | ENSGALG00000015492 |
|  | Jarso | 1:184115966 | missense_variant | MODERATE | PDZK1 | ENSGALG00000015492 |
|  | Jarso | 1:184116396 | missense_variant | MODERATE | PDZK1 | ENSGALG00000015492 |
|  | Jarso | 1:184116781 | missense_variant | MODERATE | PDZK1 | ENSGALG00000015492 |
|  | Jarso | 1:184139595 | missense_variant | MODERATE | PDZK1 | ENSGALG00000015492 |
|  | Jarso | 1:184110161 | missense_variant | MODERATE | PDZK1 | ENSGALG00000015492 |
|  | Jarso | 1:184114878 | missense_variant | MODERATE | PDZK1 | ENSGALG00000015492 |
|  | Horro | 1:184114924 | missense_variant | MODERATE | PDZK1 | ENSGALG00000015492 |
|  | Both | 1:184115022 | missense_variant | MODERATE | PDZK1 | ENSGALG00000015492 |
|  | Both | 1:184115034 | missense_variant | MODERATE | PDZK1 | ENSGALG00000015492 |
|  | Jarso | 1:184115429 | missense_variant | MODERATE | PDZK1 | ENSGALG00000015492 |
|  | Jarso | 1:184116340 | missense_variant | MODERATE | PDZK1 | ENSGALG00000015492 |
|  | Both | 1:184156588 | missense_variant | MODERATE | PDZK1 | ENSGALG00000015492 |
|  | Both | 5:25721577 | missense_variant | MODERATE | PDZK1 | ENSGALG00000015492 |
|  | Both | 5:25728694 | missense_variant | MODERATE | PDZK1 | ENSGALG00000015492 |
|  | Jarso | 5:25728524 | missense_variant | MODERATE | PDZK1 | ENSGALG00000015492 |
|  | Jarso | 5:25728810 | missense_variant | MODERATE | PLA2G4F | ENSGALG00000043591 |
|  | Both | 5:25692175 | missense_variant | MODERATE | PLA2G4F | ENSGALG00000043591 |
|  | Both | 18:5059290 | missense_variant | MODERATE | PLA2G4F | ENSGALG00000043591 |
|  | Horro | 18:5059569 | missense_variant | MODERATE | PLA2G4F | ENSGALG00000043591 |
|  | Jarso | 18:5059416 | missense_variant | MODERATE | PLA2G4F | ENSGALG00000043591 |
|  | Jarso | 18:5059620 | missense_variant | MODERATE | RASSF7 | ENSGALG00000044251 |
|  | Both | 23:2307757 | missense_variant | MODERATE | RCC1 | ENSGALG00000030097 |
|  | Both | 23:1581296 | missense_variant,  splice_region_variant | MODERATE | RPA2 | ENSGALG00000000734 |
|  | Both | 5:44316083 | missense_variant | MODERATE | FASN | ENSGALG00000040896 |
|  | Both | 5:16180716 | missense_variant | MODERATE | FASN | ENSGALG00000040896 |
|  | Both | 5:16180727 | missense_variant,  splice_region_variant | MODERATE | FASN | ENSGALG00000040896 |
|  | Both | 18:4955226 | missense_variant,  splice_region_variant | MODERATE | FASN | ENSGALG00000040896 |
|  | Both | 18:4961065 | missense_variant | MODERATE | SMPDL3B | ENSGALG00000000737 |
|  | Both | 5:14928580 | missense_variant | MODERATE | SMPDL3B | ENSGALG00000000737 |
|  | Both | 23:2369007 | missense_variant | MODERATE | SMPDL3B | ENSGALG00000000737 |
|  | Both | 18:4890032 | missense_variant | MODERATE | STXBP3 | ENSGALG00000002001 |
|  | Both | 18:4890050 | missense_variant | MODERATE | STXBP3 | ENSGALG00000002001 |
|  | Both | 18:4890109 | missense_variant | MODERATE | STXBP3 | ENSGALG00000002001 |
|  | Both | 18:4890138 | missense_variant | MODERATE | STXBP3 | ENSGALG00000002001 |
|  | Both | 18:4893531 | missense_variant | MODERATE | SYT12 | ENSGALG00000004306 |
|  | Both | 18:4893534 | missense_variant | MODERATE | TCEA3 | ENSGALG00000050173 |
|  | Both | 18:4893569 | missense_variant | MODERATE | TCEA3 | ENSGALG00000050173 |
|  | Both | 18:4893599 | missense_variant | MODERATE | TCEA3 | ENSGALG00000050173 |
|  | Horro | 18:4893657 | missense_variant | MODERATE | TCEA3 | ENSGALG00000050173 |
|  | Jarso | 18:4895580 | missense_variant | MODERATE | TCEA3 | ENSGALG00000050173 |
|  | Jarso | 18:4895617 | missense_variant | MODERATE | TCEA3 | ENSGALG00000050173 |
|  | Both | 18:4897207 | missense_variant | MODERATE | THEMIS2 | ENSGALG00000000731 |
|  | Jarso | 18:4897256 | missense_variant | MODERATE | THEMIS2 | ENSGALG00000000731 |
|  | Both | 18:4897275 | missense_variant | MODERATE | THEMIS2 | ENSGALG00000000731 |
|  | Both | 18:4890965 | missense_variant | MODERATE | THEMIS2 | ENSGALG00000000731 |
|  | Both | 18:4890977 | missense_variant,  splice_region_variant | MODERATE | THEMIS2 | ENSGALG00000000731 |
|  | Both | 23:2316977 | missense_variant | MODERATE | SIGIRR | ENSGALG00000004267 |
|  | Jarso | 23:2318329 | missense_variant | MODERATE | TTC30B | ENSGALG00000009224 |
|  | Jarso | 23:2337943 | missense_variant | MODERATE | TTC30B | ENSGALG00000009224 |
|  | Jarso | 23:2337943 | missense_variant | MODERATE | TTC7B | ENSGALG00000010680 |
|  | Jarso | 23:2338023 | missense_variant | MODERATE | TTC7B | ENSGALG00000010680 |
|  | Both | 23:2338023 | missense_variant | MODERATE | TTC7B | ENSGALG00000010680 |
|  | Both | 23:2343544 | missense_variant | MODERATE | TTC7B | ENSGALG00000010680 |
|  | Both | 23:2343544 | missense_variant | MODERATE | TTC7B | ENSGALG00000010680 |
|  | Both | 23:2258247 | missense_variant | MODERATE | TTC7B | ENSGALG00000010680 |
|  | Jarso | 1:93798557 | missense_variant | MODERATE | WDR47 | ENSGALG00000002129 |
|  | Jarso | 18:5020825 | missense_variant | MODERATE | WDR47 | ENSGALG00000002129 |
|  | Jarso | 18:5020825 | missense_variant | MODERATE | XKR8 | ENSGALG00000030305 |
|  | Jarso | 18:5020825 | missense_variant | MODERATE | Novel gene; family with sequence similarity 102 member B | ENSGALG00000001951 |
|  | Jarso | 18:5044083 | missense_variant | MODERATE | Novel gene; family with sequence similarity 102 member B | ENSGALG00000001951 |
|  | Jarso | 18:5010214 | missense_variant | MODERATE | Novel gene; family with sequence similarity 102 member B | ENSGALG00000001951 |
|  | Both | 18:5010214 | missense_variant | MODERATE | Novel gene; orthologue of AKNAD1 | ENSGALG00000051777 |
|  | Both | 18:5010214 | missense_variant | MODERATE | Novel gene; coiled-coil domain containing 57 | ENSGALG00000035675 |
|  | Both | 18:5030288 | missense_variant | MODERATE | Novel gene; orthologue of CTSL/CTSV/CTSF | ENSGALG00000013409 |
|  | Both | 18:5030288 | missense_variant | MODERATE | CTSF | ENSGALG00000013409 |
|  | Both | 18:5030288 | missense_variant | MODERATE | CTSF | ENSGALG00000013409 |
|  | Both | 1:93833439 | missense_variant | MODERATE | CTSF | ENSGALG00000013409 |
|  | Both | 18:4931598 | missense_variant | MODERATE | CTSF | ENSGALG00000047941 |
|  | Both | 18:4931598 | missense_variant | MODERATE | CTSF | ENSGALG00000047941 |
|  | Both | 18:4931623 | missense_variant | MODERATE | ANGPTL5 | ENSGALG00000017191 |
|  | Both | 18:4931623 | missense_variant | MODERATE | CEP126 | ENSGALG00000017190 |
|  | Both | 18:4931663 | missense_variant | MODERATE | CEP126 | ENSGALG00000017190 |
|  | Both | 18:4931663 | missense_variant | MODERATE | CEP126 | ENSGALG00000017190 |
|  | Both | 18:4932095 | missense_variant | MODERATE | CEP126 | ENSGALG00000017190 |
|  | Both | 18:4932095 | missense_variant | MODERATE | CEP126 | ENSGALG00000017190 |
|  | Both | 18:4932098 | missense_variant | MODERATE | CEP126 | ENSGALG00000017190 |
|  | Horro | 18:4932098 | missense_variant | MODERATE | CEP126 | ENSGALG00000017190 |
|  | Horro | 18:4932993 | missense_variant | MODERATE | CLCC1 | ENSGALG00000002106 |
|  | Both | 18:4932993 | missense_variant | MODERATE | CLCC1 | ENSGALG00000002106 |
|  | Both | 18:4937277 | missense_variant | MODERATE | CLCC1 | ENSGALG00000002106 |
|  | Both | 18:4937277 | missense_variant | MODERATE | FAM76A | ENSGALG00000040986 |
|  | Both | 18:4932999 | missense_variant | MODERATE | SIGIRR | ENSGALG00000004267 |
|  | Both | 18:4932999 | missense_variant | MODERATE | SIGIRR | ENSGALG00000004267 |
|  | Both | 23:2244305 | missense_variant | MODERATE | SIGIRR | ENSGALG00000004267 |
|  | Both | 23:2244348 | missense_variant,  splice_region_variant | MODERATE | SIGIRR | ENSGALG00000004267 |
|  | Both | 23:2244425 | missense_variant | MODERATE | LRRC45 | ENSGALG00000002877 |
|  | Both | 23:2244426 | missense_variant | MODERATE | LRRC56 | ENSGALG00000041955 |
|  | Horro | 23:2244461 | missense_variant | MODERATE | LRRC56 | ENSGALG00000041955 |
|  | Both | 23:2244551 | missense_variant | MODERATE | LRRC56 | ENSGALG00000041955 |
|  | Both | 23:2244602 | missense_variant,  splice_region_variant | MODERATE | PTDSS2 | ENSGALG00000004300 |
|  | Both | 23:2244638 | missense_variant | MODERATE | RPS6KA5 | ENSGALG00000030580 |
|  | Both | 23:2244689 | missense_variant | MODERATE | SLC25A24 | ENSGALG00000001938 |
|  | Both | 23:2247375 | missense_variant | MODERATE | SLC25A24 | ENSGALG00000001938 |
|  | Both | 23:2371460 | missense_variant | MODERATE | SYT12 | ENSGALG00000004306 |
|  | Both | 5:16144313 | missense_variant | MODERATE | SYT12 | ENSGALG00000004306 |
|  | Both | 5:16144318 | missense_variant | MODERATE | TCEA3 | ENSGALG00000050173 |
|  | Both | 5:16145119 | missense_variant | MODERATE | TCEA3 | ENSGALG00000050173 |
|  | Both | 5:16145139 | missense_variant | MODERATE | TCEA3 | ENSGALG00000050173 |
|  | Both | 5:16145141 | missense_variant | MODERATE | TCEA3 | ENSGALG00000050173 |
|  | Both | 5:16145282 | missense_variant | MODERATE | TCEA3 | ENSGALG00000050173 |
|  | Horro | 5:16147496 | missense_variant | MODERATE | TCEA3 | ENSGALG00000050173 |
|  | Horro | 5:16147507 | missense_variant | MODERATE | TCEA3 | ENSGALG00000050173 |
|  | Horro | 5:16148066 | missense_variant | MODERATE | WDR47 | ENSGALG00000002129 |
|  | Jarso | 5:16148114 | missense_variant | MODERATE | WDR47 | ENSGALG00000002129 |
|  | Both | 5:16150477 | missense_variant | MODERATE | Novel gene; orthologue of EFCAB6 | ENSGALG00000014188 |
|  | Both | 5:16145056 | missense_variant | MODERATE | Novel gene; orthologue of EFCAB6 | ENSGALG00000014188 |
|  | Both | 5:16147577 | missense_variant | MODERATE | Novel gene; orthologue of EFCAB6 | ENSGALG00000052391 |
|  | Both | 5:16150401 | missense_variant | MODERATE | Novel gene; orthologue of EFCAB6 | ENSGALG00000052391 |
|  | Both | 5:16147642 | missense_variant | MODERATE | Novel gene; orthologue of muc5f | ENSGALG00000044418 |
|  | Both | 23:2298675 | missense_variant | MODERATE | Novel gene; orthologue of PHRF1 | ENSGALG00000006873 |
|  | Both | 23:2299234 | missense_variant | MODERATE | Novel gene; orthologue of PHRF1 | ENSGALG00000006873 |
|  | Both | 23:2299235 | missense_variant | MODERATE | Novel gene; orthologue of HSPA5 | ENSGALG00000033498 |
|  | Jarso | 5:25786777 | missense_variant | MODERATE | Novel gene; orthologue of HSPA5 | ENSGALG00000033498 |
|  | Jarso | 5:25790526 | missense_variant | MODERATE | Novel gene; SITS-binding protein-like | ENSGALG00000004393 |
|  | Jarso | 5:25792138 | missense_variant | MODERATE | Novel gene; phospholipase A2 group IVE-like 5 | ENSGALG00000044307 |
|  | Jarso | 5:25792529 | missense_variant | MODERATE | Novel gene; phospholipase A2 group IVE-like 5 | ENSGALG00000044307 |
|  | Both | 5:25792908 | missense_variant | MODERATE | Novel gene; phospholipase A2 group IVE-like 2 | ENSGALG00000026584 |
|  | Jarso | 5:25782301 | missense_variant | MODERATE | Novel gene; phospholipase A2 group IVE-like 2 | ENSGALG00000026584 |
|  | Jarso | 5:25792951 | missense_variant | MODERATE | Novel gene; family with sequence similarity 102 member B | ENSGALG00000001951 |
|  | Jarso | 5:25796361 | missense_variant | MODERATE | Novel gene; orthologue of FNDC7 | ENSGALG00000052265 |
|  | Both | 18:5003153 | missense_variant | MODERATE | Novel gene; orthologue of FNDC7 | ENSGALG00000052265 |
|  | Both | 5:16104758 | missense_variant | MODERATE | Novel gene; orthologue of AKNAD1 | ENSGALG00000051777 |
|  | Jarso | 5:16102119 | missense_variant | MODERATE | Novel gene; orthologue of AKNAD1 | ENSGALG00000051777 |
|  | Jarso | 5:16102169 | missense_variant | MODERATE | Novel gene; orthologue of AKNAD1 | ENSGALG00000051777 |
|  | Both | 5:16104151 | missense_variant | MODERATE | Novel gene; orthologue of AKNAD1 | ENSGALG00000051777 |
|  | Jarso | 5:25639704 | missense_variant | MODERATE | Novel gene; orthologue of AKNAD1 | ENSGALG00000051777 |
|  | Both | 5:25652448 | missense_variant | MODERATE | Novel gene; orthologue of AKNAD1 | ENSGALG00000051777 |
|  | Both | 5:25652454 | missense_variant | MODERATE | Novel gene; orthologue of AKNAD1 | ENSGALG00000051777 |
|  | Both | 5:25653793 | missense_variant | MODERATE | Novel gene; orthologue of AKNAD1 | ENSGALG00000051777 |
|  | Both | 5:14894041 | missense_variant | MODERATE | Novel gene; orthologue of AKNAD1 | ENSGALG00000051777 |
|  | Horro | 5:14894674 | missense_variant | MODERATE | Novel gene; coiled-coil domain containing 57 | ENSGALG00000035675 |
|  | Horro | 23:2226960 | missense_variant | MODERATE | Novel gene; coiled-coil domain containing 57 | ENSGALG00000035675 |
|  | Jarso | 23:2226996 | missense_variant | MODERATE | Novel gene; guanylate binding protein 1 | ENSGALG00000000720 |
|  | Both | 23:2227187 | missense_variant | MODERATE | Novel gene; guanylate binding protein 1 | ENSGALG00000000720 |
|  | Both | 23:2228018 | missense_variant | MODERATE | Novel gene; guanylate binding protein 1 | ENSGALG00000000720 |
|  | Both | 23:2226945 | missense_variant | MODERATE | Novel gene; guanylate binding protein 1 | ENSGALG00000000720 |
|  | Both | 23:2227884 | missense_variant | MODERATE | Novel gene; orthologue of CTSL/CTSV/CTSF | ENSGALG00000047941 |
|  | Both | 23:2226938 | missense_variant | MODERATE | Novel gene; orthologue of CTSL/CTSV/CTSF | ENSGALG00000013409 |
|  | Both | 23:2329476 | missense_variant | MODERATE | ACP6 | ENSGALG00000015485 |
|  | Horro | 23:2329479 | missense_variant | MODERATE | ASAP3 | ENSGALG00000036949 |
|  | Horro | 23:2329636 | missense_variant | MODERATE | ASAP3 | ENSGALG00000036949 |
|  | Horro | 23:2329680 | missense_variant | MODERATE | ASPSCR1 | ENSGALG00000039172 |
|  | Horro | 23:2330035 | missense_variant | MODERATE | ASPSCR1 | ENSGALG00000039172 |
|  | Horro | 23:2333235 | missense_variant | MODERATE | ASPSCR1 | ENSGALG00000039172 |
|  | Horro | 23:2329821 | missense_variant | MODERATE | ASPSCR1 | ENSGALG00000039172 |
|  | Horro | 23:2330080 | missense_variant | MODERATE | ASPSCR1 | ENSGALG00000039172 |
|  | Jarso | 23:2330994 | missense_variant | MODERATE | ASPSCR1 | ENSGALG00000039172 |
|  | Jarso | 23:2331554 | missense_variant | MODERATE | BCL9 | ENSGALG00000037655 |
|  | Jarso | 23:2332285 | missense_variant | MODERATE | CENPX | ENSGALG00000043607 |
|  | Both | 23:2332332 | missense_variant | MODERATE | CEP126 | ENSGALG00000017190 |
|  | Both | 23:2332975 | missense_variant | MODERATE | CEP126 | ENSGALG00000017190 |
|  | Both | 23:2329465 | missense_variant | MODERATE | CEP126 | ENSGALG00000017190 |
|  | Both | 23:2330002 | missense_variant | MODERATE | CEP126 | ENSGALG00000017190 |
|  | Both | 23:2330070 | missense_variant | MODERATE | CEP126 | ENSGALG00000017190 |
|  | Both | 5:16085646 | missense_variant | MODERATE | CEP126 | ENSGALG00000017190 |
|  | Both | 8:1699857 | missense_variant | MODERATE | CEP126 | ENSGALG00000017190 |
|  | Both | 8:1699920 | missense_variant | MODERATE | DNAJC8 | ENSGALG00000000790 |
|  | Both | 8:1700114 | missense_variant | MODERATE | DNAJC8 | ENSGALG00000000790 |
|  | Both | 8:1700117 | missense_variant | MODERATE | EYA3 | ENSGALG00000000777 |
|  | Both | 8:1701666 | missense_variant | MODERATE | EYA3 | ENSGALG00000000777 |
|  | Both | 8:1701711 | missense_variant | MODERATE | EYA3 | ENSGALG00000000777 |
|  | Both | 8:1701913 | missense_variant | MODERATE | HENMT1 | ENSGALG00000001959 |
|  | Both | 8:1701918 | missense_variant | MODERATE | HENMT1 | ENSGALG00000001959 |
|  | Both | 8:1701944 | missense_variant | MODERATE | IGHMBP2 | ENSGALG00000004328 |
|  | Both | 8:1702047 | missense_variant | MODERATE | IGHMBP2 | ENSGALG00000004328 |
|  | Both | 8:1702092 | missense_variant | MODERATE | IGHMBP2 | ENSGALG00000004328 |
|  | Both | 8:1702095 | missense_variant | MODERATE | IGHMBP2 | ENSGALG00000004328 |
|  | Both | 8:1702107 | missense_variant | MODERATE | IGHMBP2 | ENSGALG00000004328 |
|  | Horro | 8:1702131 | missense_variant | MODERATE | IGHMBP2 | ENSGALG00000004328 |
|  | Jarso | 8:1702136 | missense_variant | MODERATE | IGHMBP2 | ENSGALG00000004328 |
|  | Jarso | 8:1702142 | missense_variant,  splice_region_variant | MODERATE | IGHMBP2 | ENSGALG00000004328 |
|  | Jarso | 8:1702157 | missense_variant | MODERATE | LRRC45 | ENSGALG00000002877 |
|  | Jarso | 8:1702196 | missense_variant | MODERATE | LRRC45 | ENSGALG00000002877 |
|  | Jarso | 8:1700134 | missense_variant | MODERATE | LRRC45 | ENSGALG00000002877 |
|  | Jarso | 8:1700077 | missense_variant | MODERATE | LRRC45 | ENSGALG00000002877 |
|  | Jarso | 8:1701278 | missense_variant | MODERATE | LRRC56 | ENSGALG00000041955 |
|  | Jarso | 8:1701294 | missense_variant | MODERATE | MYADML2 | ENSGALG00000029882 |
|  | Jarso | 8:1701318 | missense_variant | MODERATE | MYADML2 | ENSGALG00000029882 |
|  | Both | 8:1701378 | missense_variant | MODERATE | NOTUM | ENSGALG00000002900 |
|  | Jarso | 8:1701401 | missense_variant | MODERATE | OPRD1 | ENSGALG00000036782 |
|  | Jarso | 8:1701932 | missense_variant | MODERATE | PLA2G4F | ENSGALG00000043591 |
|  | Both | 8:1701975 | missense_variant | MODERATE | PLA2G4F | ENSGALG00000043591 |
|  | Both | 8:1702040 | missense_variant | MODERATE | PLA2G4F | ENSGALG00000043591 |
|  | Both | 8:1662765 | missense_variant | MODERATE | PTAFR | ENSGALG00000000783 |
|  | Both | 8:1662747 | missense_variant | MODERATE | PTAFR | ENSGALG00000000783 |
|  | Jarso | 8:1662756 | missense_variant | MODERATE | RASSF7 | ENSGALG00000044251 |
|  | Jarso | 1:68899135 | missense_variant | MODERATE | RASSF7 | ENSGALG00000044251 |
|  | Both | 1:68899245 | missense_variant | MODERATE | RASSF7 | ENSGALG00000044251 |
|  | Both | 1:68900504 | missense_variant,  splice_region_variant | MODERATE | RASSF7 | ENSGALG00000044251 |
|  | Jarso | 1:68900514 | missense_variant | MODERATE | SESN2 | ENSGALG00000037603 |
|  | Horro | 1:68900637 | missense_variant | MODERATE | TOLLIP | ENSGALG00000006697 |
|  | Jarso | 1:68902532 | missense_variant | MODERATE | SMPDL3B | ENSGALG00000000737 |
|  | Both | 23:1567861 | missense_variant | MODERATE | TCEA3 | ENSGALG00000050173 |
|  | Both | 23:1573750 | missense_variant | MODERATE | TCEA3 | ENSGALG00000050173 |
|  | Horro | 5:1779168 | missense_variant | MODERATE | TCEA3 | ENSGALG00000050173 |
|  | Horro | 5:1721448 | missense_variant | MODERATE | THEMIS2 | ENSGALG00000000731 |
|  | Both | 5:1688418 | missense_variant | MODERATE | TOLLIP | ENSGALG00000006697 |
|  | Jarso | 1:184139652 | missense_variant | MODERATE | TTC7B | ENSGALG00000010680 |
|  | Both | 18:4893500 | missense_variant | MODERATE | TTC7B | ENSGALG00000010680 |
|  | Both | 18:4935730 | missense_variant | MODERATE | TTC7B | ENSGALG00000010680 |
|  | Both | 18:4935730 | missense_variant | MODERATE | TTC7B | ENSGALG00000010680 |
|  | Both | 5:16148034 | missense_variant | MODERATE | TTC7B | ENSGALG00000010680 |
|  | Both | 5:16102199 | missense_variant | MODERATE | TTC7B | ENSGALG00000010680 |
| BCS | Both | 26:677649 | start_lost | HIGH | SYT2 | ENSGALG00000026033 |
|  | Jarso | 26:677650 | start_lost | HIGH | SYT2 | ENSGALG00000026033 |
|  | Jarso | 26:677713 | stop_gained | HIGH | SYT2 | ENSGALG00000026033 |
|  | Jarso | 26:665578 | stop_lost | HIGH | NAV1 | ENSGALG00000037370 |
|  | Both | 26:617534 | missense_variant | MODERATE | RNPEP | ENSGALG00000000374 |
|  | Both | 26:617534 | missense_variant | MODERATE | RNPEP | ENSGALG00000000374 |
|  | Both | 26:617534 | missense_variant | MODERATE | RNPEP | ENSGALG00000000374 |
|  | Both | 26:633026 | missense_variant | MODERATE | RNPEP | ENSGALG00000000374 |
|  | Both | 26:633026 | missense_variant | MODERATE | RNPEP | ENSGALG00000000374 |
|  | Jarso | 26:632810 | missense_variant | MODERATE | RNPEP | ENSGALG00000000374 |
|  | Jarso | 26:632810 | missense_variant | MODERATE | RNPEP | ENSGALG00000000374 |
|  | Jarso | 26:632880 | missense_variant | MODERATE | RNPEP | ENSGALG00000000374 |
|  | Jarso | 26:632880 | missense_variant | MODERATE | RNPEP | ENSGALG00000000374 |
|  | Jarso | 26:632904 | missense_variant | MODERATE | RNPEP | ENSGALG00000000374 |
|  | Jarso | 26:632904 | missense_variant | MODERATE | RNPEP | ENSGALG00000000374 |
|  | Jarso | 26:632922 | missense_variant | MODERATE | RNPEP | ENSGALG00000000374 |
|  | Jarso | 26:632922 | missense_variant | MODERATE | RNPEP | ENSGALG00000000374 |
|  | Both | 9:6069006 | missense_variant | MODERATE | RBP2 | ENSGALG00000005327 |
|  | Both | 9:6039848 | missense_variant | MODERATE | COPB2 | ENSGALG00000005357 |
|  | Both | 9:6043968 | missense_variant | MODERATE | COPB2 | ENSGALG00000005357 |
|  | Jarso | 9:6041048 | missense_variant | MODERATE | COPB2 | ENSGALG00000005357 |
|  | Both | 9:6033616 | missense_variant | MODERATE | MRPS22 | ENSGALG00000005367 |
|  | Both | 9:6033836 | missense_variant | MODERATE | MRPS22 | ENSGALG00000005367 |
|  | Both | 9:6034150 | missense_variant | MODERATE | MRPS22 | ENSGALG00000005367 |
|  | Both | 9:6034847 | missense_variant | MODERATE | MRPS22 | ENSGALG00000005367 |
|  | Both | 9:6037054 | missense_variant | MODERATE | MRPS22 | ENSGALG00000005367 |
|  | Jarso | 9:6033676 | missense_variant | MODERATE | MRPS22 | ENSGALG00000005367 |
|  | Jarso | 9:6034840 | missense_variant | MODERATE | MRPS22 | ENSGALG00000005367 |
|  | Jarso | 9:6034894 | missense_variant | MODERATE | MRPS22 | ENSGALG00000005367 |
|  | Both | 7:9651852 | missense_variant | MODERATE | SLC39A10 | ENSGALG00000007777 |
|  | Both | 7:9652275 | missense_variant | MODERATE | SLC39A10 | ENSGALG00000007777 |
|  | Both | 7:9652299 | missense_variant | MODERATE | SLC39A10 | ENSGALG00000007777 |
|  | Both | 7:9652336 | missense_variant | MODERATE | SLC39A10 | ENSGALG00000007777 |
|  | Both | 7:9660505 | missense_variant | MODERATE | SLC39A10 | ENSGALG00000007777 |
|  | Both | 1:107903465 | missense_variant | MODERATE | SETD4 | ENSGALG00000016024 |
|  | Both | 1:107906269 | missense_variant | MODERATE | SETD4 | ENSGALG00000016024 |
|  | Both | 1:107907002 | missense_variant | MODERATE | SETD4 | ENSGALG00000016024 |
|  | Both | 1:107908089 | missense_variant | MODERATE | SETD4 | ENSGALG00000016024 |
|  | Both | 1:107911182 | missense_variant | MODERATE | SETD4 | ENSGALG00000016024 |
|  | Both | 1:107911647 | missense_variant | MODERATE | SETD4 | ENSGALG00000016024 |
|  | Jarso | 1:107906930 | missense_variant | MODERATE | SETD4 | ENSGALG00000016024 |
|  | Both | 1:107913678 | missense_variant | MODERATE | CBR3 | ENSGALG00000016027 |
|  | Both | 1:107913772 | missense_variant | MODERATE | CBR3 | ENSGALG00000016027 |
|  | Both | 1:107939730 | missense_variant | MODERATE | DOP1B | ENSGALG00000016036 |
|  | Both | 1:107939730 | missense_variant | MODERATE | DOP1B | ENSGALG00000016036 |
|  | Both | 1:107941630 | missense_variant | MODERATE | DOP1B | ENSGALG00000016036 |
|  | Both | 1:107941630 | missense_variant | MODERATE | DOP1B | ENSGALG00000016036 |
|  | Both | 1:107945658 | missense_variant | MODERATE | DOP1B | ENSGALG00000016036 |
|  | Both | 1:107945658 | missense_variant | MODERATE | DOP1B | ENSGALG00000016036 |
|  | Both | 1:107946787 | missense_variant | MODERATE | DOP1B | ENSGALG00000016036 |
|  | Both | 1:107946787 | missense_variant | MODERATE | DOP1B | ENSGALG00000016036 |
|  | Both | 1:107967464 | missense_variant | MODERATE | DOP1B | ENSGALG00000016036 |
|  | Both | 1:107967464 | missense_variant | MODERATE | DOP1B | ENSGALG00000016036 |
|  | Both | 1:107967620 | missense_variant | MODERATE | DOP1B | ENSGALG00000016036 |
|  | Both | 1:107967620 | missense_variant | MODERATE | DOP1B | ENSGALG00000016036 |
|  | Jarso | 1:107942910 | missense_variant | MODERATE | DOP1B | ENSGALG00000016036 |
|  | Jarso | 1:107942910 | missense_variant | MODERATE | DOP1B | ENSGALG00000016036 |
|  | Both | 1:108007473 | missense_variant | MODERATE | CHAF1B | ENSGALG00000016042 |
|  | Both | 1:108007473 | missense_variant | MODERATE | CHAF1B | ENSGALG00000016042 |
|  | Both | 1:108007473 | missense_variant | MODERATE | CHAF1B | ENSGALG00000016042 |
|  | Both | 1:108016271 | missense_variant | MODERATE | CHAF1B | ENSGALG00000016042 |
|  | Both | 1:108016271 | missense_variant | MODERATE | CHAF1B | ENSGALG00000016042 |
|  | Both | 1:108016271 | missense_variant | MODERATE | CHAF1B | ENSGALG00000016042 |
|  | Both | 1:108017970 | missense_variant | MODERATE | CHAF1B | ENSGALG00000016042 |
|  | Both | 1:108017970 | missense_variant | MODERATE | CHAF1B | ENSGALG00000016042 |
|  | Both | 1:108017970 | missense_variant | MODERATE | CHAF1B | ENSGALG00000016042 |
|  | Jarso | 1:108017988 | missense_variant | MODERATE | CHAF1B | ENSGALG00000016042 |
|  | Jarso | 1:108017988 | missense_variant | MODERATE | CHAF1B | ENSGALG00000016042 |
|  | Jarso | 1:108017988 | missense_variant | MODERATE | CHAF1B | ENSGALG00000016042 |
|  | Both | 26:786562 | missense_variant | MODERATE | PHLDA3 | ENSGALG00000019802 |
|  | Both | 26:677722 | missense_variant | MODERATE | SYT2 | ENSGALG00000026033 |
|  | Both | 26:677756 | missense_variant | MODERATE | SYT2 | ENSGALG00000026033 |
|  | Horro | 26:677682 | missense_variant | MODERATE | SYT2 | ENSGALG00000026033 |
|  | Both | 9:6115462 | missense_variant | MODERATE | NMNAT3 | ENSGALG00000026187 |
|  | Both | 9:6115462 | missense_variant | MODERATE | NMNAT3 | ENSGALG00000026187 |
|  | Both | 9:6122182 | missense_variant | MODERATE | NMNAT3 | ENSGALG00000026187 |
|  | Both | 9:6122182 | missense_variant | MODERATE | NMNAT3 | ENSGALG00000026187 |
|  | Both | 9:6122182 | missense_variant | MODERATE | NMNAT3 | ENSGALG00000026187 |
|  | Both | 9:6122326 | missense_variant | MODERATE | NMNAT3 | ENSGALG00000026187 |
|  | Both | 9:6122326 | missense_variant | MODERATE | NMNAT3 | ENSGALG00000026187 |
|  | Both | 9:6122326 | missense_variant | MODERATE | NMNAT3 | ENSGALG00000026187 |
|  | Both | 2:116365139 | missense_variant | MODERATE | Novel gene; solute carrier organic anion transporter family member 5A1 | ENSGALG00000029842 |
|  | Both | 2:96930271 | missense_variant | MODERATE | CEP192 | ENSGALG00000035625 |
|  | Both | 2:96936677 | missense_variant | MODERATE | CEP192 | ENSGALG00000035625 |
|  | Both | 2:96942985 | missense_variant | MODERATE | CEP192 | ENSGALG00000035625 |
|  | Both | 2:96947508 | missense_variant | MODERATE | CEP192 | ENSGALG00000035625 |
|  | Both | 2:96947521 | missense_variant | MODERATE | CEP192 | ENSGALG00000035625 |
|  | Horro | 2:96925016 | missense_variant | MODERATE | CEP192 | ENSGALG00000035625 |
|  | Horro | 2:96943652 | missense_variant | MODERATE | CEP192 | ENSGALG00000035625 |
|  | Horro | 2:96945511 | missense_variant | MODERATE | CEP192 | ENSGALG00000035625 |
|  | Both | 1:107995182 | missense_variant | MODERATE | MORC4 | ENSGALG00000036556 |
|  | Both | 1:107995182 | missense_variant | MODERATE | MORC4 | ENSGALG00000036556 |
|  | Both | 1:107995354 | missense_variant | MODERATE | MORC4 | ENSGALG00000036556 |
|  | Both | 1:107995354 | missense_variant | MODERATE | MORC4 | ENSGALG00000036556 |
|  | Both | 1:107995567 | missense_variant | MODERATE | MORC4 | ENSGALG00000036556 |
|  | Both | 1:107995567 | missense_variant | MODERATE | MORC4 | ENSGALG00000036556 |
|  | Jarso | 1:107996355 | missense_variant | MODERATE | MORC4 | ENSGALG00000036556 |
|  | Jarso | 1:107996355 | missense_variant | MODERATE | MORC4 | ENSGALG00000036556 |
|  | Both | 26:655548 | missense_variant | MODERATE | NAV1 | ENSGALG00000037370 |
|  | Both | 26:655548 | missense_variant | MODERATE | NAV1 | ENSGALG00000037370 |
|  | Both | 26:655548 | missense_variant | MODERATE | NAV1 | ENSGALG00000037370 |
|  | Both | 26:655548 | missense_variant | MODERATE | NAV1 | ENSGALG00000037370 |
|  | Both | 26:655548 | missense_variant | MODERATE | NAV1 | ENSGALG00000037370 |
|  | Both | 26:655548 | missense_variant | MODERATE | NAV1 | ENSGALG00000037370 |
|  | Both | 26:655548 | missense_variant | MODERATE | NAV1 | ENSGALG00000037370 |
|  | Both | 26:656577 | missense_variant | MODERATE | NAV1 | ENSGALG00000037370 |
|  | Both | 26:656577 | missense_variant | MODERATE | NAV1 | ENSGALG00000037370 |
|  | Both | 26:656577 | missense_variant | MODERATE | NAV1 | ENSGALG00000037370 |
|  | Both | 26:656577 | missense_variant | MODERATE | NAV1 | ENSGALG00000037370 |
|  | Both | 26:656577 | missense_variant | MODERATE | NAV1 | ENSGALG00000037370 |
|  | Both | 26:656577 | missense_variant | MODERATE | NAV1 | ENSGALG00000037370 |
|  | Both | 26:656577 | missense_variant | MODERATE | NAV1 | ENSGALG00000037370 |
|  | Both | 26:657147 | missense_variant | MODERATE | NAV1 | ENSGALG00000037370 |
|  | Both | 26:657147 | missense_variant | MODERATE | NAV1 | ENSGALG00000037370 |
|  | Both | 26:657147 | missense_variant | MODERATE | NAV1 | ENSGALG00000037370 |
|  | Both | 26:657147 | missense_variant | MODERATE | NAV1 | ENSGALG00000037370 |
|  | Both | 26:657147 | missense_variant | MODERATE | NAV1 | ENSGALG00000037370 |
|  | Both | 26:657147 | missense_variant | MODERATE | NAV1 | ENSGALG00000037370 |
|  | Both | 26:657147 | missense_variant | MODERATE | NAV1 | ENSGALG00000037370 |
|  | Both | 26:657315 | missense_variant | MODERATE | NAV1 | ENSGALG00000037370 |
|  | Both | 26:657315 | missense_variant | MODERATE | NAV1 | ENSGALG00000037370 |
|  | Both | 26:657315 | missense_variant | MODERATE | NAV1 | ENSGALG00000037370 |
|  | Both | 26:657315 | missense_variant | MODERATE | NAV1 | ENSGALG00000037370 |
|  | Both | 26:657315 | missense_variant | MODERATE | NAV1 | ENSGALG00000037370 |
|  | Both | 26:657315 | missense_variant | MODERATE | NAV1 | ENSGALG00000037370 |
|  | Both | 26:657315 | missense_variant | MODERATE | NAV1 | ENSGALG00000037370 |
|  | Both | 26:665309 | missense_variant | MODERATE | NAV1 | ENSGALG00000037370 |
|  | Both | 26:665553 | missense_variant | MODERATE | NAV1 | ENSGALG00000037370 |
|  | Horro | 26:665063 | missense_variant | MODERATE | NAV1 | ENSGALG00000037370 |
|  | Horro | 26:665388 | missense_variant | MODERATE | NAV1 | ENSGALG00000037370 |
|  | Both | 9:6482896 | missense_variant | MODERATE | CLSTN2 | ENSGALG00000037387 |
|  | Both | 7:9690766 | missense_variant | MODERATE | Novel gene; baculoviral IAP repeat-containing protein 5.1-like | ENSGALG00000037678 |
|  | Both | 7:21015867 | missense_variant | MODERATE | KCNH7 | ENSGALG00000038543 |
|  | Both | 7:21015867 | missense_variant | MODERATE | KCNH7 | ENSGALG00000038543 |
|  | Both | 7:21027524 | missense_variant | MODERATE | KCNH7 | ENSGALG00000038543 |
|  | Both | 7:21027524 | missense_variant | MODERATE | KCNH7 | ENSGALG00000038543 |
|  | Both | 7:21032617 | missense_variant | MODERATE | KCNH7 | ENSGALG00000038543 |
|  | Both | 7:21032617 | missense_variant | MODERATE | KCNH7 | ENSGALG00000038543 |
|  | Both | 2:116339973 | missense_variant | MODERATE | SULF1 | ENSGALG00000039553 |
|  | Both | 2:116339973 | missense_variant | MODERATE | SULF1 | ENSGALG00000039553 |
|  | Both | 2:116342942 | missense_variant | MODERATE | SULF1 | ENSGALG00000039553 |
|  | Both | 2:116342957 | missense_variant | MODERATE | SULF1 | ENSGALG00000039553 |
|  | Both | 7:21044218 | missense_variant | MODERATE | IFIH1 | ENSGALG00000041192 |
|  | Both | 7:21059842 | missense_variant | MODERATE | IFIH1 | ENSGALG00000041192 |
|  | Horro | 7:21047257 | missense_variant | MODERATE | IFIH1 | ENSGALG00000041192 |
|  | Jarso | 7:21044205 | missense_variant | MODERATE | IFIH1 | ENSGALG00000041192 |
|  | Jarso | 7:21044211 | missense_variant | MODERATE | IFIH1 | ENSGALG00000041192 |
|  | Jarso | 7:21044227 | missense_variant | MODERATE | IFIH1 | ENSGALG00000041192 |
|  | Jarso | 7:21044302 | missense_variant | MODERATE | IFIH1 | ENSGALG00000041192 |
|  | Jarso | 7:21055754 | missense_variant | MODERATE | IFIH1 | ENSGALG00000041192 |
|  | Jarso | 2:116352654 | missense_variant | MODERATE | Novel gene | ENSGALG00000055117 |
|  | Both | 9:6035643 | missense_variant, splice_region_variant | MODERATE | MRPS22 | ENSGALG00000005367 |
|  | Both | 7:9690112 | missense_variant, splice_region_variant | MODERATE | Novel gene; orthologue of DNAH7 | ENSGALG00000007841 |
|  | Jarso | 1:107903567 | missense_variant, splice_region_variant | MODERATE | SETD4 | ENSGALG00000016024 |
|  | Jarso | 1:107904784 | missense_variant, splice_region_variant | MODERATE | SETD4 | ENSGALG00000016024 |
|  | Horro | 2:96934153 | missense_variant, splice_region_variant | MODERATE | CEP192 | ENSGALG00000035625 |
| BW | Jarso | 4:87250774 | splice_acceptor_variant,non_coding_transcript_variant | HIGH | Novel lncRNA | ENSGALG00000052288 |
|  | Jarso | 4:87250774 | splice_acceptor_variant,non_coding_transcript_variant | HIGH | Novel lncRNA | ENSGALG00000052288 |
|  | Jarso | 4:87013070 | splice_donor_variant,non_coding_transcript_variant | HIGH | Novel lncRNA | ENSGALG00000052582 |
|  | Jarso | 4:87013070 | splice_donor_variant,non_coding_transcript_variant | HIGH | Novel lncRNA | ENSGALG00000052582 |
|  | Jarso | 4:87013070 | splice_donor_variant,non_coding_transcript_variant | HIGH | Novel lncRNA | ENSGALG00000052582 |
|  | Jarso | 4:87015629 | splice_donor_variant,non_coding_transcript_variant | HIGH | Novel lncRNA | ENSGALG00000052582 |
|  | Jarso | 4:87015629 | splice_donor_variant,non_coding_transcript_variant | HIGH | Novel lncRNA | ENSGALG00000052582 |
|  | Jarso | 4:87015629 | splice_donor_variant,non_coding_transcript_variant | HIGH | Novel lncRNA | ENSGALG00000052582 |
|  | Jarso | 4:87015630 | splice_donor_variant,non_coding_transcript_variant | HIGH | Novel lncRNA | ENSGALG00000052582 |
|  | Jarso | 4:87015630 | splice_donor_variant,non_coding_transcript_variant | HIGH | Novel lncRNA | ENSGALG00000052582 |
|  | Jarso | 4:87015630 | splice_donor_variant,non_coding_transcript_variant | HIGH | Novel lncRNA | ENSGALG00000052582 |
|  | Jarso | 4:8649433 | stop_gained | HIGH | CHM | ENSGALG00000006906 |
|  | Both | 5:15928612 | missense_variant | MODERATE | CRACR2B | ENSGALG00000006868 |
|  | Both | 5:15928692 | missense_variant | MODERATE | CRACR2B | ENSGALG00000006868 |
|  | Both | 5:15944963 | missense_variant | MODERATE | CRACR2B | ENSGALG00000006868 |
|  | Both | 5:15944963 | missense_variant | MODERATE | CRACR2B | ENSGALG00000006868 |
|  | Both | 5:15954385 | missense_variant | MODERATE | CRACR2B | ENSGALG00000006868 |
|  | Both | 5:15954568 | missense_variant | MODERATE | CRACR2B | ENSGALG00000006868 |
|  | Both | 4:8541293 | missense_variant | MODERATE | DACH2 | ENSGALG00000006886 |
|  | Both | 4:8541293 | missense_variant | MODERATE | DACH2 | ENSGALG00000006886 |
|  | Both | 4:8541293 | missense_variant | MODERATE | DACH2 | ENSGALG00000006886 |
|  | Both | 4:8578042 | missense_variant | MODERATE | CHM | ENSGALG00000006906 |
|  | Both | 4:8578042 | missense_variant | MODERATE | CHM | ENSGALG00000006906 |
|  | Both | 4:8578042 | missense_variant | MODERATE | CHM | ENSGALG00000006906 |
|  | Both | 4:8578058 | missense_variant | MODERATE | CHM | ENSGALG00000006906 |
|  | Both | 4:8578058 | missense_variant | MODERATE | CHM | ENSGALG00000006906 |
|  | Both | 4:8578058 | missense_variant | MODERATE | CHM | ENSGALG00000006906 |
|  | Both | 4:8578094 | missense_variant | MODERATE | CHM | ENSGALG00000006906 |
|  | Both | 4:8578094 | missense_variant | MODERATE | CHM | ENSGALG00000006906 |
|  | Both | 4:8578094 | missense_variant | MODERATE | CHM | ENSGALG00000006906 |
|  | Both | 4:8582511 | missense_variant | MODERATE | CHM | ENSGALG00000006906 |
|  | Both | 4:8582511 | missense_variant | MODERATE | CHM | ENSGALG00000006906 |
|  | Both | 4:8582511 | missense_variant | MODERATE | CHM | ENSGALG00000006906 |
|  | Both | 4:8582550 | missense_variant | MODERATE | CHM | ENSGALG00000006906 |
|  | Both | 4:8582550 | missense_variant | MODERATE | CHM | ENSGALG00000006906 |
|  | Both | 4:8582550 | missense_variant | MODERATE | CHM | ENSGALG00000006906 |
|  | Both | 4:8615376 | missense_variant | MODERATE | CHM | ENSGALG00000006906 |
|  | Both | 4:8615376 | missense_variant | MODERATE | CHM | ENSGALG00000006906 |
|  | Both | 4:8615376 | missense_variant | MODERATE | CHM | ENSGALG00000006906 |
|  | Both | 4:8663556 | missense_variant | MODERATE | POF1B | ENSGALG00000006919 |
|  | Both | 4:8669940 | missense_variant | MODERATE | POF1B | ENSGALG00000006919 |
|  | Both | 4:8719649 | missense_variant | MODERATE | APOOL | ENSGALG00000006978 |
|  | Both | 4:8719649 | missense_variant | MODERATE | APOOL | ENSGALG00000006978 |
|  | Both | 4:8724440 | missense_variant | MODERATE | APOOL | ENSGALG00000006978 |
|  | Both | 4:8724440 | missense_variant | MODERATE | APOOL | ENSGALG00000006978 |
|  | Both | 4:8724452 | missense_variant | MODERATE | APOOL | ENSGALG00000006978 |
|  | Both | 4:8724452 | missense_variant | MODERATE | APOOL | ENSGALG00000006978 |
|  | Both | 8:18880568 | missense_variant | MODERATE | ADGRL4 | ENSGALG00000008890 |
|  | Both | 8:18880568 | missense_variant | MODERATE | ADGRL4 | ENSGALG00000008890 |
|  | Both | 8:18880568 | missense_variant | MODERATE | ADGRL4 | ENSGALG00000008890 |
|  | Both | 8:18931672 | missense_variant | MODERATE | ADGRL4 | ENSGALG00000008890 |
|  | Both | 8:18931672 | missense_variant | MODERATE | ADGRL4 | ENSGALG00000008890 |
|  | Both | 8:18931672 | missense_variant | MODERATE | ADGRL4 | ENSGALG00000008890 |
|  | Both | 8:18931672 | missense_variant | MODERATE | ADGRL4 | ENSGALG00000008890 |
|  | Both | 8:18931672 | missense_variant | MODERATE | ADGRL4 | ENSGALG00000008890 |
|  | Both | 8:18931672 | missense_variant | MODERATE | ADGRL4 | ENSGALG00000008890 |
|  | Both | 8:18953693 | missense_variant | MODERATE | ADGRL4 | ENSGALG00000008890 |
|  | Both | 8:18953693 | missense_variant | MODERATE | ADGRL4 | ENSGALG00000008890 |
|  | Both | 8:18953693 | missense_variant | MODERATE | ADGRL4 | ENSGALG00000008890 |
|  | Both | 8:18953693 | missense_variant | MODERATE | ADGRL4 | ENSGALG00000008890 |
|  | Both | 8:18953693 | missense_variant | MODERATE | ADGRL4 | ENSGALG00000008890 |
|  | Both | 8:18953905 | missense_variant | MODERATE | ADGRL4 | ENSGALG00000008890 |
|  | Both | 8:18953905 | missense_variant | MODERATE | ADGRL4 | ENSGALG00000008890 |
|  | Both | 8:18953905 | missense_variant | MODERATE | ADGRL4 | ENSGALG00000008890 |
|  | Both | 8:18953905 | missense_variant | MODERATE | ADGRL4 | ENSGALG00000008890 |
|  | Both | 8:18953905 | missense_variant | MODERATE | ADGRL4 | ENSGALG00000008890 |
|  | Both | 8:18953913 | missense_variant | MODERATE | ADGRL4 | ENSGALG00000008890 |
|  | Both | 8:18953913 | missense_variant | MODERATE | ADGRL4 | ENSGALG00000008890 |
|  | Both | 8:18953913 | missense_variant | MODERATE | ADGRL4 | ENSGALG00000008890 |
|  | Both | 8:18953913 | missense_variant | MODERATE | ADGRL4 | ENSGALG00000008890 |
|  | Both | 8:18953913 | missense_variant | MODERATE | ADGRL4 | ENSGALG00000008890 |
|  | Both | 8:18953980 | missense_variant | MODERATE | ADGRL4 | ENSGALG00000008890 |
|  | Both | 8:18953980 | missense_variant | MODERATE | ADGRL4 | ENSGALG00000008890 |
|  | Both | 8:18953980 | missense_variant | MODERATE | ADGRL4 | ENSGALG00000008890 |
|  | Both | 8:18953980 | missense_variant | MODERATE | ADGRL4 | ENSGALG00000008890 |
|  | Both | 8:18953980 | missense_variant | MODERATE | ADGRL4 | ENSGALG00000008890 |
|  | Both | 5:16054283 | missense_variant | MODERATE | IRF7 | ENSGALG00000014297 |
|  | Both | 5:16054999 | missense_variant | MODERATE | IRF7 | ENSGALG00000014297 |
|  | Both | 5:16055184 | missense_variant | MODERATE | IRF7 | ENSGALG00000014297 |
|  | Both | 5:16055358 | missense_variant | MODERATE | IRF7 | ENSGALG00000014297 |
|  | Both | 5:15965792 | missense_variant | MODERATE | PNPLA2 | ENSGALG00000014569 |
|  | Both | 5:15968123 | missense_variant | MODERATE | PNPLA2 | ENSGALG00000014569 |
|  | Both | 4:87573809 | missense_variant | MODERATE | LRRTM1 | ENSGALG00000015967 |
|  | Both | 5:15989493 | missense_variant | MODERATE | RPLP2 | ENSGALG00000034811 |
|  | Both | 5:15989493 | missense_variant | MODERATE | RPLP2 | ENSGALG00000034811 |
|  | Both | 8:18990001 | missense_variant | MODERATE | VTG1 | ENSGALG00000039354 |
|  | Both | 8:18990034 | missense_variant | MODERATE | VTG1 | ENSGALG00000039354 |
|  | Both | 8:19008822 | missense_variant | MODERATE | VTG1 | ENSGALG00000039354 |
|  | Both | 8:19018122 | missense_variant | MODERATE | VTG1 | ENSGALG00000039354 |
|  | Both | 8:19018914 | missense_variant | MODERATE | VTG1 | ENSGALG00000039354 |
|  | Both | 8:19019286 | missense_variant | MODERATE | VTG1 | ENSGALG00000039354 |
|  | Both | 8:19020655 | missense_variant | MODERATE | VTG1 | ENSGALG00000039354 |
|  | Both | 8:19020723 | missense_variant | MODERATE | VTG1 | ENSGALG00000039354 |
|  | Both | 5:15995343 | missense_variant | MODERATE | Novel gene | ENSGALG00000040729 |
|  | Both | 5:15995409 | missense_variant | MODERATE | Novel gene | ENSGALG00000040729 |
|  | Both | 5:15995490 | missense_variant | MODERATE | Novel gene | ENSGALG00000040729 |
|  | Both | 5:15997310 | missense_variant | MODERATE | Novel gene | ENSGALG00000040729 |
|  | Both | 5:15997456 | missense_variant | MODERATE | Novel gene | ENSGALG00000040729 |
|  | Both | 4:8706076 | missense_variant | MODERATE | Novel gene; orthologue of 2010106E10Rik | ENSGALG00000042151 |
|  | Both | 4:8712338 | missense_variant | MODERATE | Novel gene; orthologue of 2010106E10Rik | ENSGALG00000042151 |
|  | Both | 5:16056998 | missense_variant | MODERATE | Novel gene; homologue of malignant fibrous histiocytoma-amplified sequence 1 | ENSGALG00000044313 |
|  | Both | 5:16059514 | missense_variant | MODERATE | Novel gene; homologue of malignant fibrous histiocytoma-amplified sequence 1 | ENSGALG00000044313 |
|  | Both | 5:16059514 | missense_variant | MODERATE | Novel gene; homologue of malignant fibrous histiocytoma-amplified sequence 1 | ENSGALG00000044313 |
|  | Both | 5:16059589 | missense_variant | MODERATE | Novel gene; homologue of malignant fibrous histiocytoma-amplified sequence 1 | ENSGALG00000044313 |
|  | Both | 5:16059589 | missense_variant | MODERATE | Novel gene; homologue of malignant fibrous histiocytoma-amplified sequence 1 | ENSGALG00000044313 |
|  | Both | 5:15923667 | missense_variant | MODERATE | Novel gene; orthologue of si:ch211-247i17.1 | ENSGALG00000049256 |
|  | Both | 5:15925392 | missense_variant | MODERATE | Novel gene; orthologue of si:ch211-247i17.1 | ENSGALG00000049256 |
|  | Both | 5:16004600 | missense_variant | MODERATE | Novel gene | ENSGALG00000054561 |
|  | Both | 5:16005483 | missense_variant | MODERATE | Novel gene | ENSGALG00000054561 |
|  | Both | 5:16006069 | missense_variant | MODERATE | Novel gene | ENSGALG00000054561 |
|  | Both | 5:16006069 | missense_variant | MODERATE | Novel gene | ENSGALG00000054561 |
|  | Both | 5:16006999 | missense_variant | MODERATE | Novel gene | ENSGALG00000054561 |
|  | Both | 5:16006999 | missense_variant | MODERATE | Novel gene | ENSGALG00000054561 |
|  | Both | 5:16007951 | missense_variant | MODERATE | Novel gene | ENSGALG00000054561 |
|  | Both | 5:16007951 | missense_variant | MODERATE | Novel gene | ENSGALG00000054561 |
|  | Both | 5:16008020 | missense_variant | MODERATE | Novel gene | ENSGALG00000054561 |
|  | Both | 5:16008020 | missense_variant | MODERATE | Novel gene | ENSGALG00000054561 |
|  | Both | 5:15995539 | missense_variant, splice_region_variant | MODERATE | Novel gene; orthologue of PIDD1 | ENSGALG00000040729 |
| CES | Jarso | 7:22129908 | splice_donor_variant | HIGH | Novel gene; rac GTPase-activating protein 1-like | ENSGALG00000011252 |
|  | Both | 19:9461689 | start_lost | HIGH | TNFAIP1 | ENSGALG00000005737 |
|  | Both | 7:22140065 | stop_gained | HIGH | DNPEP | ENSGALG00000011318 |
|  | Both | 23:1065499 | missense_variant | MODERATE | EDN2 | ENSGALG00000000667 |
|  | Horro | 23:1066556 | missense_variant | MODERATE | EDN2 | ENSGALG00000000667 |
|  | Both | 7:22136695 | missense_variant | MODERATE | GMPPA | ENSGALG00000002417 |
|  | Jarso | 7:22135239 | missense_variant | MODERATE | GMPPA | ENSGALG00000002417 |
|  | Horro | 14:6146635 | missense_variant | MODERATE | SOX8 | ENSGALG00000005263 |
|  | Horro | 14:6149162 | missense_variant | MODERATE | SOX8 | ENSGALG00000005263 |
|  | Jarso | 19:9406821 | missense_variant | MODERATE | NLK | ENSGALG00000005699 |
|  | Horro | 19:9445828 | missense_variant | MODERATE | TMEM97 | ENSGALG00000005702 |
|  | Both | 19:9502856 | missense_variant | MODERATE | TNFAIP1 | ENSGALG00000005737 |
|  | Both | 19:9502856 | missense_variant | MODERATE | TNFAIP1 | ENSGALG00000005737 |
|  | Both | 19:9502856 | missense_variant | MODERATE | TNFAIP1 | ENSGALG00000005737 |
|  | Both | 7:22067273 | missense_variant | MODERATE | OBSL1 | ENSGALG00000011242 |
|  | Both | 7:22067812 | missense_variant | MODERATE | OBSL1 | ENSGALG00000011242 |
|  | Both | 7:22068109 | missense_variant | MODERATE | OBSL1 | ENSGALG00000011242 |
|  | Both | 7:22068457 | missense_variant | MODERATE | OBSL1 | ENSGALG00000011242 |
|  | Both | 7:22068466 | missense_variant | MODERATE | OBSL1 | ENSGALG00000011242 |
|  | Jarso | 7:22070269 | missense_variant | MODERATE | OBSL1 | ENSGALG00000011242 |
|  | Both | 7:22089854 | missense_variant | MODERATE | ASIC4 | ENSGALG00000011250 |
|  | Both | 7:22104555 | missense_variant | MODERATE | ASIC4 | ENSGALG00000011250 |
|  | Both | 7:22104988 | missense_variant | MODERATE | ASIC4 | ENSGALG00000011250 |
|  | Jarso | 7:22104951 | missense_variant | MODERATE | ASIC4 | ENSGALG00000011250 |
|  | Both | 7:22129244 | missense_variant | MODERATE | Novel gene; rac GTPase-activating protein 1-like | ENSGALG00000011252 |
|  | Both | 7:22129275 | missense_variant | MODERATE | Novel gene; rac GTPase-activating protein 1-like | ENSGALG00000011252 |
|  | Both | 7:22129570 | missense_variant | MODERATE | Novel gene; rac GTPase-activating protein 1-like | ENSGALG00000011252 |
|  | Both | 7:22131528 | missense_variant | MODERATE | Novel gene; rac GTPase-activating protein 1-like | ENSGALG00000011252 |
|  | Both | 7:22131582 | missense_variant | MODERATE | Novel gene; rac GTPase-activating protein 1-like | ENSGALG00000011252 |
|  | Both | 7:22131594 | missense_variant | MODERATE | Novel gene; rac GTPase-activating protein 1-like | ENSGALG00000011252 |
|  | Both | 7:22131665 | missense_variant | MODERATE | Novel gene; rac GTPase-activating protein 1-like | ENSGALG00000011252 |
|  | Both | 7:22132233 | missense_variant | MODERATE | Novel gene; rac GTPase-activating protein 1-like | ENSGALG00000011252 |
|  | Both | 7:22132241 | missense_variant | MODERATE | Novel gene; rac GTPase-activating protein 1-like | ENSGALG00000011252 |
|  | Both | 7:22132408 | missense_variant | MODERATE | Novel gene; rac GTPase-activating protein 1-like | ENSGALG00000011252 |
|  | Jarso | 7:22129892 | missense_variant | MODERATE | Novel gene; rac GTPase-activating protein 1-like | ENSGALG00000011252 |
|  | Jarso | 7:22130954 | missense_variant | MODERATE | Novel gene; rac GTPase-activating protein 1-like | ENSGALG00000011252 |
|  | Jarso | 7:22132436 | missense_variant | MODERATE | Novel gene; rac GTPase-activating protein 1-like | ENSGALG00000011252 |
|  | Both | 7:22172410 | missense_variant | MODERATE | DES | ENSGALG00000011306 |
|  | Both | 7:22183728 | missense_variant | MODERATE | DNPEP | ENSGALG00000011318 |
|  | Both | 7:22183728 | missense_variant | MODERATE | DNPEP | ENSGALG00000011318 |
|  | Both | 7:22183743 | missense_variant | MODERATE | DNPEP | ENSGALG00000011318 |
|  | Both | 7:22183743 | missense_variant | MODERATE | DNPEP | ENSGALG00000011318 |
|  | Both | 7:22212269 | missense_variant | MODERATE | GLB1L | ENSGALG00000011328 |
|  | Both | 7:22212421 | missense_variant | MODERATE | GLB1L | ENSGALG00000011328 |
|  | Both | 7:22213418 | missense_variant | MODERATE | GLB1L | ENSGALG00000011328 |
|  | Both | 7:22213429 | missense_variant | MODERATE | GLB1L | ENSGALG00000011328 |
|  | Both | 7:22213493 | missense_variant | MODERATE | GLB1L | ENSGALG00000011328 |
|  | Both | 7:22214702 | missense_variant | MODERATE | GLB1L | ENSGALG00000011328 |
|  | Both | 7:22214763 | missense_variant | MODERATE | GLB1L | ENSGALG00000011328 |
|  | Both | 7:22214953 | missense_variant | MODERATE | GLB1L | ENSGALG00000011328 |
|  | Both | 7:22216126 | missense_variant | MODERATE | GLB1L | ENSGALG00000011328 |
|  | Horro | 7:22213478 | missense_variant | MODERATE | GLB1L | ENSGALG00000011328 |
|  | Both | 7:22218861 | missense_variant | MODERATE | ANKZF1 | ENSGALG00000011332 |
|  | Both | 7:22218861 | missense_variant | MODERATE | ANKZF1 | ENSGALG00000011332 |
|  | Both | 7:22220016 | missense_variant | MODERATE | ANKZF1 | ENSGALG00000011332 |
|  | Both | 7:22220016 | missense_variant | MODERATE | ANKZF1 | ENSGALG00000011332 |
|  | Both | 7:22220736 | missense_variant | MODERATE | ANKZF1 | ENSGALG00000011332 |
|  | Both | 7:22220736 | missense_variant | MODERATE | ANKZF1 | ENSGALG00000011332 |
|  | Both | 7:22220834 | missense_variant | MODERATE | ANKZF1 | ENSGALG00000011332 |
|  | Both | 7:22220834 | missense_variant | MODERATE | ANKZF1 | ENSGALG00000011332 |
|  | Both | 7:22221830 | missense_variant | MODERATE | ANKZF1 | ENSGALG00000011332 |
|  | Both | 7:22221830 | missense_variant | MODERATE | ANKZF1 | ENSGALG00000011332 |
|  | Both | 7:22221882 | missense_variant | MODERATE | ANKZF1 | ENSGALG00000011332 |
|  | Both | 7:22221882 | missense_variant | MODERATE | ANKZF1 | ENSGALG00000011332 |
|  | Horro | 7:22223252 | missense_variant | MODERATE | ANKZF1 | ENSGALG00000011332 |
|  | Horro | 7:22223252 | missense_variant | MODERATE | ANKZF1 | ENSGALG00000011332 |
|  | Both | 7:22230117 | missense_variant | MODERATE | ATG9A | ENSGALG00000011649 |
|  | Both | 1:7994754 | missense_variant | MODERATE | DCLRE1C | ENSGALG00000013926 |
|  | Both | 1:7994754 | missense_variant | MODERATE | DCLRE1C | ENSGALG00000013926 |
|  | Both | 1:8001069 | missense_variant | MODERATE | DCLRE1C | ENSGALG00000013926 |
|  | Both | 1:8001118 | missense_variant | MODERATE | DCLRE1C | ENSGALG00000013926 |
|  | Jarso | 1:7996385 | missense_variant | MODERATE | DCLRE1C | ENSGALG00000013926 |
|  | Jarso | 1:7996385 | missense_variant | MODERATE | DCLRE1C | ENSGALG00000013926 |
|  | Jarso | 1:159181580 | missense_variant | MODERATE | KLHL1 | ENSGALG00000016907 |
|  | Jarso | 1:159181580 | missense_variant | MODERATE | KLHL1 | ENSGALG00000016907 |
|  | Horro | 1:190150129 | missense_variant | MODERATE | ME3 | ENSGALG00000017246 |
|  | Horro | 1:190150129 | missense_variant | MODERATE | ME3 | ENSGALG00000017246 |
|  | Jarso | 1:190149989 | missense_variant | MODERATE | ME3 | ENSGALG00000017246 |
|  | Jarso | 1:190149989 | missense_variant | MODERATE | ME3 | ENSGALG00000017246 |
|  | Both | 1:74769560 | missense_variant | MODERATE | Novel gene; Rho guanine nucleotide exchange factor 5 | ENSGALG00000017276 |
|  | Both | 1:74783485 | missense_variant | MODERATE | Novel gene; Rho guanine nucleotide exchange factor 5 | ENSGALG00000017276 |
|  | Jarso | 1:74784868 | missense_variant | MODERATE | Novel gene; Rho guanine nucleotide exchange factor 5 | ENSGALG00000017276 |
|  | Both | 7:22239676 | missense_variant | MODERATE | Novel gene; AN1-type zinc finger protein 2B-like | ENSGALG00000029226 |
|  | Both | 7:22240080 | missense_variant | MODERATE | Novel gene; AN1-type zinc finger protein 2B-like | ENSGALG00000029226 |
|  | Jarso | 7:22241060 | missense_variant | MODERATE | Novel gene; AN1-type zinc finger protein 2B-like | ENSGALG00000029226 |
|  | Both | 7:22233205 | missense_variant | MODERATE | ABCB6 | ENSGALG00000029672 |
|  | Both | 7:22233552 | missense_variant | MODERATE | ABCB6 | ENSGALG00000029672 |
|  | Both | 7:22233552 | missense_variant | MODERATE | ABCB6 | ENSGALG00000029672 |
|  | Both | 7:22233606 | missense_variant | MODERATE | ABCB6 | ENSGALG00000029672 |
|  | Both | 7:22233606 | missense_variant | MODERATE | ABCB6 | ENSGALG00000029672 |
|  | Both | 7:22233651 | missense_variant | MODERATE | ABCB6 | ENSGALG00000029672 |
|  | Both | 7:22233651 | missense_variant | MODERATE | ABCB6 | ENSGALG00000029672 |
|  | Both | 7:22234316 | missense_variant | MODERATE | ABCB6 | ENSGALG00000029672 |
|  | Both | 7:22234316 | missense_variant | MODERATE | ABCB6 | ENSGALG00000029672 |
|  | Both | 7:22235192 | missense_variant | MODERATE | ABCB6 | ENSGALG00000029672 |
|  | Both | 7:22235192 | missense_variant | MODERATE | ABCB6 | ENSGALG00000029672 |
|  | Both | 7:22237393 | missense_variant | MODERATE | ABCB6 | ENSGALG00000029672 |
|  | Horro | 7:22234105 | missense_variant | MODERATE | ABCB6 | ENSGALG00000029672 |
|  | Horro | 7:22234105 | missense_variant | MODERATE | ABCB6 | ENSGALG00000029672 |
|  | Horro | 7:22234400 | missense_variant | MODERATE | ABCB6 | ENSGALG00000029672 |
|  | Horro | 7:22234400 | missense_variant | MODERATE | ABCB6 | ENSGALG00000029672 |
|  | Both | 3:949473 | missense_variant | MODERATE | FANCL | ENSGALG00000030022 |
|  | Jarso | 3:949391 | missense_variant | MODERATE | FANCL | ENSGALG00000030022 |
|  | Both | 1:8037747 | missense_variant | MODERATE | DMTF1 | ENSGALG00000030844 |
|  | Both | 1:8037747 | missense_variant | MODERATE | DMTF1 | ENSGALG00000030844 |
|  | Both | 1:8049605 | missense_variant | MODERATE | DMTF1 | ENSGALG00000030844 |
|  | Both | 1:8049605 | missense_variant | MODERATE | DMTF1 | ENSGALG00000030844 |
|  | Jarso | 1:8033336 | missense_variant | MODERATE | DMTF1 | ENSGALG00000030844 |
|  | Jarso | 1:8033336 | missense_variant | MODERATE | DMTF1 | ENSGALG00000030844 |
|  | Both | 2:46096571 | missense_variant | MODERATE | SFRP4 | ENSGALG00000031997 |
|  | Both | 3:937276 | missense_variant | MODERATE | VRK2 | ENSGALG00000032770 |
|  | Both | 3:937276 | missense_variant | MODERATE | VRK2 | ENSGALG00000032770 |
|  | Both | 3:937276 | missense_variant | MODERATE | VRK2 | ENSGALG00000032770 |
|  | Both | 3:944956 | missense_variant | MODERATE | VRK2 | ENSGALG00000032770 |
|  | Both | 7:22078454 | missense_variant | MODERATE | CHPF | ENSGALG00000032915 |
|  | Both | 7:22078454 | missense_variant | MODERATE | CHPF | ENSGALG00000032915 |
|  | Both | 7:22078742 | missense_variant | MODERATE | CHPF | ENSGALG00000032915 |
|  | Both | 7:22078742 | missense_variant | MODERATE | CHPF | ENSGALG00000032915 |
|  | Horro | 7:22078025 | missense_variant | MODERATE | CHPF | ENSGALG00000032915 |
|  | Horro | 7:22078025 | missense_variant | MODERATE | CHPF | ENSGALG00000032915 |
|  | Horro | 7:22078271 | missense_variant | MODERATE | CHPF | ENSGALG00000032915 |
|  | Horro | 7:22078271 | missense_variant | MODERATE | CHPF | ENSGALG00000032915 |
|  | Jarso | 7:22079904 | missense_variant | MODERATE | CHPF | ENSGALG00000032915 |
|  | Jarso | 7:22079904 | missense_variant | MODERATE | CHPF | ENSGALG00000032915 |
|  | Jarso | 2:45992650 | missense_variant | MODERATE | STARD3NL | ENSGALG00000033038 |
|  | Jarso | 2:45992650 | missense_variant | MODERATE | STARD3NL | ENSGALG00000033038 |
|  | Both | 7:22241947 | missense_variant | MODERATE | Novel gene; family with sequence similarity 134 member A | ENSGALG00000033452 |
|  | Horro | 7:22242532 | missense_variant | MODERATE | Novel gene; family with sequence similarity 134 member A | ENSGALG00000033452 |
|  | Jarso | 7:22241972 | missense_variant | MODERATE | Novel gene; family with sequence similarity 134 member A | ENSGALG00000033452 |
|  | Both | 7:22187212 | missense_variant | MODERATE | PTPRN | ENSGALG00000033811 |
|  | Both | 7:22187555 | missense_variant | MODERATE | PTPRN | ENSGALG00000033811 |
|  | Both | 7:22187556 | missense_variant | MODERATE | PTPRN | ENSGALG00000033811 |
|  | Both | 7:22187685 | missense_variant | MODERATE | PTPRN | ENSGALG00000033811 |
|  | Both | 7:22187687 | missense_variant | MODERATE | PTPRN | ENSGALG00000033811 |
|  | Both | 7:22187711 | missense_variant | MODERATE | PTPRN | ENSGALG00000033811 |
|  | Both | 7:22187768 | missense_variant | MODERATE | PTPRN | ENSGALG00000033811 |
|  | Both | 7:22188301 | missense_variant | MODERATE | PTPRN | ENSGALG00000033811 |
|  | Both | 7:22188402 | missense_variant | MODERATE | PTPRN | ENSGALG00000033811 |
|  | Both | 7:22191338 | missense_variant | MODERATE | PTPRN | ENSGALG00000033811 |
|  | Jarso | 7:22187050 | missense_variant | MODERATE | PTPRN | ENSGALG00000033811 |
|  | Both | 1:7977810 | missense_variant | MODERATE | Novel gene; heat shock protein family A (Hsp70) member 14 | ENSGALG00000035201 |
|  | Both | 1:7977810 | missense_variant | MODERATE | Novel gene; heat shock protein family A (Hsp70) member 14 | ENSGALG00000035201 |
|  | Both | 1:7978255 | missense_variant | MODERATE | Novel gene; heat shock protein family A (Hsp70) member 14 | ENSGALG00000035201 |
|  | Both | 1:7978255 | missense_variant | MODERATE | Novel gene; heat shock protein family A (Hsp70) member 14 | ENSGALG00000035201 |
|  | Both | 1:7980993 | missense_variant | MODERATE | Novel gene; heat shock protein family A (Hsp70) member 14 | ENSGALG00000035201 |
|  | Both | 1:7980993 | missense_variant | MODERATE | Novel gene; heat shock protein family A (Hsp70) member 14 | ENSGALG00000035201 |
|  | Horro | 1:7977845 | missense_variant | MODERATE | Novel gene; heat shock protein family A (Hsp70) member 14 | ENSGALG00000035201 |
|  | Horro | 1:7977845 | missense_variant | MODERATE | Novel gene; heat shock protein family A (Hsp70) member 14 | ENSGALG00000035201 |
|  | Jarso | 1:7977825 | missense_variant | MODERATE | Novel gene; heat shock protein family A (Hsp70) member 14 | ENSGALG00000035201 |
|  | Jarso | 1:7977825 | missense_variant | MODERATE | Novel gene; heat shock protein family A (Hsp70) member 14 | ENSGALG00000035201 |
|  | Both | 2:45934111 | missense_variant | MODERATE | TRANK1 | ENSGALG00000037629 |
|  | Both | 2:45934111 | missense_variant | MODERATE | TRANK1 | ENSGALG00000037629 |
|  | Both | 2:45935908 | missense_variant | MODERATE | TRANK1 | ENSGALG00000037629 |
|  | Jarso | 2:45935953 | missense_variant | MODERATE | TRANK1 | ENSGALG00000037629 |
|  | Jarso | 19:9353194 | missense_variant | MODERATE | NOS2 | ENSGALG00000038096 |
|  | Both | 2:45972907 | missense_variant | MODERATE | MLH1 | ENSGALG00000039169 |
|  | Both | 2:45973844 | missense_variant | MODERATE | MLH1 | ENSGALG00000039169 |
|  | Horro | 2:45962576 | missense_variant | MODERATE | MLH1 | ENSGALG00000039169 |
|  | Jarso | 2:45972692 | missense_variant | MODERATE | MLH1 | ENSGALG00000039169 |
|  | Both | 7:22140853 | missense_variant | MODERATE | Novel gene; SPEG complex locus | ENSGALG00000043198 |
|  | Both | 7:22140853 | missense_variant | MODERATE | Novel gene; SPEG complex locus | ENSGALG00000043198 |
|  | Both | 7:22140853 | missense_variant | MODERATE | Novel gene; SPEG complex locus | ENSGALG00000043198 |
|  | Both | 7:22140913 | missense_variant | MODERATE | Novel gene; SPEG complex locus | ENSGALG00000043198 |
|  | Both | 7:22140913 | missense_variant | MODERATE | Novel gene; SPEG complex locus | ENSGALG00000043198 |
|  | Both | 7:22140913 | missense_variant | MODERATE | Novel gene; SPEG complex locus | ENSGALG00000043198 |
|  | Both | 7:22141019 | missense_variant | MODERATE | Novel gene; SPEG complex locus | ENSGALG00000043198 |
|  | Both | 7:22141019 | missense_variant | MODERATE | Novel gene; SPEG complex locus | ENSGALG00000043198 |
|  | Both | 7:22141019 | missense_variant | MODERATE | Novel gene; SPEG complex locus | ENSGALG00000043198 |
|  | Both | 7:22141069 | missense_variant | MODERATE | Novel gene; SPEG complex locus | ENSGALG00000043198 |
|  | Both | 7:22141069 | missense_variant | MODERATE | Novel gene; SPEG complex locus | ENSGALG00000043198 |
|  | Both | 7:22141069 | missense_variant | MODERATE | Novel gene; SPEG complex locus | ENSGALG00000043198 |
|  | Both | 7:22141145 | missense_variant | MODERATE | Novel gene; SPEG complex locus | ENSGALG00000043198 |
|  | Both | 7:22141145 | missense_variant | MODERATE | Novel gene; SPEG complex locus | ENSGALG00000043198 |
|  | Both | 7:22141145 | missense_variant | MODERATE | Novel gene; SPEG complex locus | ENSGALG00000043198 |
|  | Both | 7:22141214 | missense_variant | MODERATE | Novel gene; SPEG complex locus | ENSGALG00000043198 |
|  | Both | 7:22141214 | missense_variant | MODERATE | Novel gene; SPEG complex locus | ENSGALG00000043198 |
|  | Both | 7:22141214 | missense_variant | MODERATE | Novel gene; SPEG complex locus | ENSGALG00000043198 |
|  | Both | 7:22141451 | missense_variant | MODERATE | Novel gene; SPEG complex locus | ENSGALG00000043198 |
|  | Both | 7:22141451 | missense_variant | MODERATE | Novel gene; SPEG complex locus | ENSGALG00000043198 |
|  | Both | 7:22141451 | missense_variant | MODERATE | Novel gene; SPEG complex locus | ENSGALG00000043198 |
|  | Both | 7:22143079 | missense_variant | MODERATE | Novel gene; SPEG complex locus | ENSGALG00000043198 |
|  | Both | 7:22143079 | missense_variant | MODERATE | Novel gene; SPEG complex locus | ENSGALG00000043198 |
|  | Both | 7:22143079 | missense_variant | MODERATE | Novel gene; SPEG complex locus | ENSGALG00000043198 |
|  | Both | 7:22143193 | missense_variant | MODERATE | Novel gene; SPEG complex locus | ENSGALG00000043198 |
|  | Both | 7:22143193 | missense_variant | MODERATE | Novel gene; SPEG complex locus | ENSGALG00000043198 |
|  | Both | 7:22143193 | missense_variant | MODERATE | Novel gene; SPEG complex locus | ENSGALG00000043198 |
|  | Both | 7:22144424 | missense_variant | MODERATE | Novel gene; SPEG complex locus | ENSGALG00000043198 |
|  | Both | 7:22144424 | missense_variant | MODERATE | Novel gene; SPEG complex locus | ENSGALG00000043198 |
|  | Both | 7:22144424 | missense_variant | MODERATE | Novel gene; SPEG complex locus | ENSGALG00000043198 |
|  | Both | 7:22144486 | missense_variant | MODERATE | Novel gene; SPEG complex locus | ENSGALG00000043198 |
|  | Both | 7:22144486 | missense_variant | MODERATE | Novel gene; SPEG complex locus | ENSGALG00000043198 |
|  | Both | 7:22144486 | missense_variant | MODERATE | Novel gene; SPEG complex locus | ENSGALG00000043198 |
|  | Both | 7:22144808 | missense_variant | MODERATE | Novel gene; SPEG complex locus | ENSGALG00000043198 |
|  | Both | 7:22144808 | missense_variant | MODERATE | Novel gene; SPEG complex locus | ENSGALG00000043198 |
|  | Both | 7:22144808 | missense_variant | MODERATE | Novel gene; SPEG complex locus | ENSGALG00000043198 |
|  | Both | 7:22148635 | missense_variant | MODERATE | Novel gene; SPEG complex locus | ENSGALG00000043198 |
|  | Both | 7:22148635 | missense_variant | MODERATE | Novel gene; SPEG complex locus | ENSGALG00000043198 |
|  | Both | 7:22148635 | missense_variant | MODERATE | Novel gene; SPEG complex locus | ENSGALG00000043198 |
|  | Both | 7:22157063 | missense_variant | MODERATE | Novel gene; SPEG complex locus | ENSGALG00000043198 |
|  | Both | 7:22157063 | missense_variant | MODERATE | Novel gene; SPEG complex locus | ENSGALG00000043198 |
|  | Both | 7:22157063 | missense_variant | MODERATE | Novel gene; SPEG complex locus | ENSGALG00000043198 |
|  | Both | 7:22157278 | missense_variant | MODERATE | Novel gene; SPEG complex locus | ENSGALG00000043198 |
|  | Both | 7:22157278 | missense_variant | MODERATE | Novel gene; SPEG complex locus | ENSGALG00000043198 |
|  | Both | 7:22157278 | missense_variant | MODERATE | Novel gene; SPEG complex locus | ENSGALG00000043198 |
|  | Both | 7:22158046 | missense_variant | MODERATE | Novel gene; SPEG complex locus | ENSGALG00000043198 |
|  | Both | 7:22158046 | missense_variant | MODERATE | Novel gene; SPEG complex locus | ENSGALG00000043198 |
|  | Both | 7:22158046 | missense_variant | MODERATE | Novel gene; SPEG complex locus | ENSGALG00000043198 |
|  | Both | 7:22159548 | missense_variant | MODERATE | Novel gene; SPEG complex locus | ENSGALG00000043198 |
|  | Both | 7:22159557 | missense_variant | MODERATE | Novel gene; SPEG complex locus | ENSGALG00000043198 |
|  | Jarso | 7:22142933 | missense_variant | MODERATE | Novel gene; SPEG complex locus | ENSGALG00000043198 |
|  | Jarso | 7:22142933 | missense_variant | MODERATE | Novel gene; SPEG complex locus | ENSGALG00000043198 |
|  | Jarso | 7:22142933 | missense_variant | MODERATE | Novel gene; SPEG complex locus | ENSGALG00000043198 |
|  | Both | 1:74756721 | missense_variant | MODERATE | Novel gene; NOBOX oogenesis homeobox | ENSGALG00000043916 |
|  | Both | 1:74756721 | missense_variant | MODERATE | Novel gene; NOBOX oogenesis homeobox | ENSGALG00000043916 |
|  | Both | 1:74756765 | missense_variant | MODERATE | Novel gene; NOBOX oogenesis homeobox | ENSGALG00000043916 |
|  | Both | 1:74756765 | missense_variant | MODERATE | Novel gene; NOBOX oogenesis homeobox | ENSGALG00000043916 |
|  | Jarso | 1:74755895 | missense_variant | MODERATE | Novel gene; NOBOX oogenesis homeobox | ENSGALG00000043916 |
|  | Jarso | 1:74755895 | missense_variant | MODERATE | Novel gene; NOBOX oogenesis homeobox | ENSGALG00000043916 |
|  | Horro | 14:6204545 | missense_variant | MODERATE | Novel gene; lipase maturation factor 1 | ENSGALG00000044187 |
|  | Both | 1:189998919 | missense_variant | MODERATE | TMEM135 | ENSGALG00000047269 |
|  | Both | 1:189999012 | missense_variant | MODERATE | TMEM135 | ENSGALG00000047269 |
|  | Both | 1:189999055 | missense_variant | MODERATE | TMEM135 | ENSGALG00000047269 |
|  | Both | 1:75650019 | missense_variant | MODERATE | MFAP5 | ENSGALG00000047320 |
|  | Horro | 4:71945884 | missense_variant | MODERATE |  | ENSGALG00000050883 |
|  | Both | 7:22082910 | missense_variant | MODERATE | LOC424199 | ENSGALG00000053241 |
|  | Both | 7:22083080 | missense_variant | MODERATE | LOC424199 | ENSGALG00000053241 |
|  | Both | 7:22083470 | missense_variant | MODERATE | LOC424199 | ENSGALG00000053241 |
|  | Both | 7:22084009 | missense_variant | MODERATE | LOC424199 | ENSGALG00000053241 |
|  | Horro | 7:22082795 | missense_variant | MODERATE | LOC424199 | ENSGALG00000053241 |
|  | Horro | 7:22083928 | missense_variant | MODERATE | LOC424199 | ENSGALG00000053241 |
|  | Jarso | 7:22083985 | missense_variant | MODERATE | LOC424199 | ENSGALG00000053241 |
|  | Jarso | 7:22084010 | missense_variant | MODERATE | LOC424199 | ENSGALG00000053241 |
|  | Jarso | 7:22084302 | missense_variant | MODERATE | LOC424199 | ENSGALG00000053241 |
|  | Jarso | 19:9406821 | missense_variant, splice_region_variant | MODERATE | NLK | ENSGALG00000005699 |
|  | Both | 1:8001681 | missense_variant, splice_region_variant | MODERATE | DCLRE1C | ENSGALG00000013926 |
| *Eimeria* | Jarso | 18:5812386 | missense_variant | MODERATE | TOM1L1 | ENSGALG00000003011 |
|  | Jarso | 18:5812394 | missense_variant | MODERATE | TOM1L1 | ENSGALG00000003011 |
|  | Jarso | 18:5812410 | missense_variant | MODERATE | TOM1L1 | ENSGALG00000003011 |
|  | Jarso | 18:5812430 | missense_variant | MODERATE | TOM1L1 | ENSGALG00000003011 |
|  | Jarso | 18:5812742 | missense_variant | MODERATE | TOM1L1 | ENSGALG00000003011 |
|  | Jarso | 18:5812755 | missense_variant | MODERATE | TOM1L1 | ENSGALG00000003011 |
|  | Jarso | 18:5812757 | missense_variant | MODERATE | TOM1L1 | ENSGALG00000003011 |
|  | Both | 18:5812449 | missense_variant | MODERATE | TOM1L1 | ENSGALG00000003011 |
|  | Both | 18:5812479 | missense_variant | MODERATE | TOM1L1 | ENSGALG00000003011 |
|  | Both | 18:5812658 | missense_variant | MODERATE | TOM1L1 | ENSGALG00000003011 |
|  | Both | 18:5812688 | missense_variant | MODERATE | TOM1L1 | ENSGALG00000003011 |
|  | Both | 18:5812688 | missense_variant | MODERATE | TOM1L1 | ENSGALG00000003011 |
|  | Both | 18:5830068 | missense_variant | MODERATE | TOM1L1 | ENSGALG00000003011 |
|  | Both | 18:5830068 | missense_variant | MODERATE | TOM1L1 | ENSGALG00000003011 |
|  | Jarso | 18:5844937 | missense_variant | MODERATE | STXBP4 | ENSGALG00000003033 |
|  | Jarso | 18:5844937 | missense_variant | MODERATE | STXBP4 | ENSGALG00000003033 |
|  | Jarso | 18:5844937 | missense_variant | MODERATE | STXBP4 | ENSGALG00000003033 |
|  | Jarso | 18:5851235 | missense_variant | MODERATE | STXBP4 | ENSGALG00000003033 |
|  | Jarso | 18:5851235 | missense_variant | MODERATE | STXBP4 | ENSGALG00000003033 |
|  | Jarso | 18:5851235 | missense_variant | MODERATE | STXBP4 | ENSGALG00000003033 |
|  | Both | 18:5841797 | missense_variant | MODERATE | STXBP4 | ENSGALG00000003033 |
|  | Both | 18:5848040 | missense_variant | MODERATE | STXBP4 | ENSGALG00000003033 |
|  | Both | 18:5848040 | missense_variant | MODERATE | STXBP4 | ENSGALG00000003033 |
|  | Both | 18:5848040 | missense_variant | MODERATE | STXBP4 | ENSGALG00000003033 |
|  | Both | 18:5848064 | missense_variant | MODERATE | STXBP4 | ENSGALG00000003033 |
|  | Both | 18:5848064 | missense_variant | MODERATE | STXBP4 | ENSGALG00000003033 |
|  | Both | 18:5848064 | missense_variant | MODERATE | STXBP4 | ENSGALG00000003033 |
|  | Both | 18:5852034 | missense_variant | MODERATE | STXBP4 | ENSGALG00000003033 |
|  | Both | 18:5852034 | missense_variant | MODERATE | STXBP4 | ENSGALG00000003033 |
|  | Both | 18:5852034 | missense_variant | MODERATE | STXBP4 | ENSGALG00000003033 |
|  | Both | 18:5854568 | missense_variant | MODERATE | STXBP4 | ENSGALG00000003033 |
|  | Both | 18:5854568 | missense_variant | MODERATE | STXBP4 | ENSGALG00000003033 |
|  | Both | 18:5854568 | missense_variant | MODERATE | STXBP4 | ENSGALG00000003033 |
|  | Both | 18:5856452 | missense_variant | MODERATE | STXBP4 | ENSGALG00000003033 |
|  | Both | 18:5856452 | missense_variant | MODERATE | STXBP4 | ENSGALG00000003033 |
|  | Both | 18:5856452 | missense_variant | MODERATE | STXBP4 | ENSGALG00000003033 |
|  | Both | 18:5857278 | missense_variant | MODERATE | STXBP4 | ENSGALG00000003033 |
|  | Both | 18:5857278 | missense_variant | MODERATE | STXBP4 | ENSGALG00000003033 |
|  | Both | 18:5857278 | missense_variant | MODERATE | STXBP4 | ENSGALG00000003033 |
|  | Both | 18:5857803 | missense_variant | MODERATE | STXBP4 | ENSGALG00000003033 |
|  | Both | 18:5857803 | missense_variant | MODERATE | STXBP4 | ENSGALG00000003033 |
|  | Both | 18:5857803 | missense_variant | MODERATE | STXBP4 | ENSGALG00000003033 |
|  | Both | 18:5836346 | missense_variant | MODERATE | COX11 | ENSGALG00000027190 |
|  | Both | 18:5836347 | missense_variant | MODERATE | COX11 | ENSGALG00000027190 |
|  | Jarso | 18:5851291 | missense_variant, splice_region_variant | MODERATE | STXBP4 | ENSGALG00000003033 |
|  | Jarso | 18:5851291 | missense_variant, splice_region_variant | MODERATE | STXBP4 | ENSGALG00000003033 |
|  | Jarso | 18:5851291 | missense_variant, splice_region_variant | MODERATE | STXBP4 | ENSGALG00000003033 |
| MDV | Horro | 11:19722411 | missense_variant | MODERATE | DHODH | ENSGALG00000000802 |
|  | Horro | 11:19813178 | missense_variant | MODERATE | PHLPP2 | ENSGALG00000000875 |
|  | Horro | 11:19872995 | missense_variant | MODERATE | KARS | ENSGALG00000000907 |
|  | Horro | 11:19872995 | missense_variant | MODERATE | KARS | ENSGALG00000000907 |
|  | Horro | 12:19681462 | missense_variant | MODERATE | OXTR | ENSGALG00000003138 |
|  | Horro | 12:19684407 | missense_variant | MODERATE | OXTR | ENSGALG00000003138 |
|  | Horro | 12:19644420 | missense_variant | MODERATE | LMCD1 | ENSGALG00000008349 |
|  | Horro | 12:19649740 | missense_variant | MODERATE | LMCD1 | ENSGALG00000008349 |
|  | Horro | 12:19697373 | missense_variant | MODERATE | RAD18 | ENSGALG00000008368 |
|  | Horro | 12:19697373 | missense_variant | MODERATE | RAD18 | ENSGALG00000008368 |
|  | Horro | 12:19710778 | missense_variant | MODERATE | RAD18 | ENSGALG00000008368 |
|  | Horro | 12:19710778 | missense_variant | MODERATE | RAD18 | ENSGALG00000008368 |
|  | Horro | 12:19710778 | missense_variant | MODERATE | RAD18 | ENSGALG00000008368 |
|  | Horro | 12:19710778 | missense_variant | MODERATE | RAD18 | ENSGALG00000008368 |
|  | Horro | 12:19710778 | missense_variant | MODERATE | RAD18 | ENSGALG00000008368 |
|  | Horro | 12:19710850 | missense_variant | MODERATE | RAD18 | ENSGALG00000008368 |
|  | Horro | 12:19710850 | missense_variant | MODERATE | RAD18 | ENSGALG00000008368 |
|  | Horro | 12:19710850 | missense_variant | MODERATE | RAD18 | ENSGALG00000008368 |
|  | Horro | 12:19710850 | missense_variant | MODERATE | RAD18 | ENSGALG00000008368 |
|  | Horro | 12:19710850 | missense_variant | MODERATE | RAD18 | ENSGALG00000008368 |
|  | Jarso | 3:35885737 | missense_variant | MODERATE | WDR64 | ENSGALG00000010760 |
|  | Jarso | 3:35971141 | missense_variant | MODERATE | PIGM | ENSGALG00000010768 |
|  | Both | 3:35971503 | missense_variant | MODERATE | PIGM | ENSGALG00000010768 |
|  | Horro | 11:19820041 | missense_variant, splice_region_variant | MODERATE | PHLPP2 | ENSGALG00000000875 |
| SG | Horro | 3:38888263 | missense_variant | MODERATE | MAP3K21 | ENSGALG00000011008 |
|  | Horro | 3:38888263 | missense_variant | MODERATE | MAP3K21 | ENSGALG00000011008 |
|  | Horro | 3:38889493 | missense_variant | MODERATE | MAP3K21 | ENSGALG00000011008 |
|  | Horro | 3:38889493 | missense_variant | MODERATE | MAP3K21 | ENSGALG00000011008 |
|  | Jarso | 3:38926477 | missense_variant | MODERATE | MAP3K21 | ENSGALG00000011008 |
|  | Jarso | 3:38926477 | missense_variant | MODERATE | MAP3K21 | ENSGALG00000011008 |
|  | Jarso | 3:38941646 | missense_variant | MODERATE | PCNX2 | ENSGALG00000011013 |
|  | Horro | 3:38941880 | missense_variant | MODERATE | PCNX2 | ENSGALG00000011013 |
|  | Jarso | 3:38942318 | missense_variant | MODERATE | PCNX2 | ENSGALG00000011013 |
